# Global burden of lower respiratory infections during the last three decades

**DOI:** 10.3389/fpubh.2022.1028525

**Published:** 2023-01-09

**Authors:** Saeid Safiri, Ata Mahmoodpoor, Ali-Asghar Kolahi, Seyed Aria Nejadghaderi, Mark J. M. Sullman, Mohammad Ali Mansournia, Khalil Ansarin, Gary S. Collins, Jay S. Kaufman, Morteza Abdollahi

**Affiliations:** ^1^Social Determinants of Health Research Center, Department of Community Medicine, Faculty of Medicine, Tabriz University of Medical Sciences, Tabriz, Iran; ^2^Tuberculosis and Lung Diseases Research Centre, Tabriz University of Medical Sciences, Tabriz, Iran; ^3^Department of Anesthesiology, Faculty of Medicine, Tabriz University of Medical Sciences, Tabriz, Iran; ^4^Social Determinants of Health Research Center, Shahid Beheshti University of Medical Sciences, Tehran, Iran; ^5^Research Centre for Integrative Medicine in Aging, Aging Research Institute, Tabriz University of Medical Sciences, Tabriz, Iran; ^6^Systematic Review and Meta-analysis Expert Group (SRMEG), Universal Scientific Education and Research Network (USERN), Tehran, Iran; ^7^Department of Life and Health Sciences, University of Nicosia, Nicosia, Cyprus; ^8^Department of Social Sciences, University of Nicosia, Nicosia, Cyprus; ^9^Department of Epidemiology and Biostatistics, School of Public Health, Tehran University of Medical Sciences, Tehran, Iran; ^10^Rahat Breath and Sleep Research Center, Tabriz University of Medical Sciences, Tabriz, Iran; ^11^NDORMS, Centre for Statistics in Medicine, Botnar Research Centre, University of Oxford, Oxford, United Kingdom; ^12^NIHR Oxford Biomedical Research Centre, Oxford University Hospitals NHS Foundation Trust, Oxford, United Kingdom; ^13^Department of Epidemiology, Biostatistics and Occupational Health, Faculty of Medicine, McGill University, Montreal, QC, Canada

**Keywords:** lower respiratory infection, risk factor, incidence, mortality, respiratory infection, epidemiology

## Abstract

**Background:**

Lower respiratory infections (LRIs) cause a substantial mortality, morbidity and economic burden. The present study reported the global, regional and national burden of LRIs and their attributable risk factors in 204 countries and territories, between 1990 and 2019, by age, sex, etiology, and Socio-demographic Index (SDI).

**Methods:**

Using publicly available data from the Global Burden of Disease (GBD) study 2019, we reported the incidence, deaths and disability-adjusted life-years (DALYs), due to LRIs. Estimates were presented as counts and age-standardized rates per 100,000 population with their associated uncertainty intervals (UIs).

**Results:**

Globally, in 2019 there were 488.9 million (95% UI: 457.6 to 522.6) incident cases and 2.4 million (2.3–2.7) deaths due to LRIs. The global age-standardized incidence and death rates for LRIs were 6,295 (5,887.4–6,737.3) and 34.3 (31.1–37.9) per 100,000 in 2019, which represents a 23.9% (22.5–25.4) and 48.5% (42.9–54.0) decrease, respectively since 1990. In 2019, Guinea [12,390.4 (11,495.5–13,332.8)], Chad [12,208.1 (11,289.3–13,202.5)] and India [11,862.1 (11,087.0–12,749.0)] had the three highest age-standardized incidence rates of LRI. Equatorial Guinea [−52.7% (95% UI: −55.8 to −49.3)], Chile [−50.2% (95% UI: −53.4 to −47.0)] and Albania [−48.6% (95% UI: −51.7 to −45.3)] showed the largest decreases from 1990 to 2019. In 2019, a decrease in the incidence rate of LRI was observed at the global level up to the 25–29 age group, then the incidence rates increased with age. The burden of LRIs decreased with increasing SDI at both the regional and national levels. Globally, child wasting (33.1%), household air pollution from solid fuels (24.9%) and a lack of access to handwashing facilities (14.4%) made the largest contributions to the LRI burden in 2019.

**Conclusions:**

Although the burden of LRIs decreased over the period 1990–2019, LRIs still contribute to a large number of incident cases, deaths and DALYs. Preventative programs with a focus on reducing exposure to attributable risk factors should be implemented, especially in less developed countries.

## Introduction

Respiratory tract infections, especially lower respiratory infections (LRIs), are a leading cause of death and disability, particularly in children younger than 5 years old (1). The etiology of LRIs can be viral (e.g., respiratory syncytial virus (RSV), parainfluenza virus, influenza virus, adenovirus and coronavirus) or bacterial [e.g., *Streptococcus pneumonia* and *Haemophilus influenzae* type b (Hib)] (2, 3). Moreover, *Streptococcus pneumonia* is the principal aetiological agent of LRIs and is responsible for around half of the deaths (3). LRIs also impose a substantial economic burden, with LRIs due to RSV costing an average of €3,452 and €299 for inpatient and outpatient treatment, respectively, which can be higher if there are any underlying diseases or follow-up is required (4).

Pleural effusion, heart failure, anemia, pneumothorax, bronchiectasis and hemoptysis are all potential complications of LRIs (5, 6). In 2016, LRIs accounted for 2.38 million deaths worldwide (3), with Sub-Saharan Africa (66.4 deaths per 100 000 people) having the highest death rate (3). LRIs are negatively associated with the level of development, with the lowest rates of mortality and morbidity being found in developed countries (1, 3).

The burden of LRIs have been previously reported using the Global Burden of Diseases (GBD) 2015 and 2016 data (1, 3). These papers primarily concentrated on the burden of LRIs by etiological agents, while the burden of LRIs attributable to potential risk factors have not been previously reported, which is important from a preventative perspective (1, 3). Furthermore, a recently published article reported the incidence and deaths attributable to LRIs at the global, regional and national levels, with a focus on age and sex patterns (7). This study also reported the population-attributable fractions for each risk factor (7), but did not report the LRI attributable burden by sociodemographic index (SDI) or the disability-adjusted life-years (DALYs) (7). Furthermore, another study using GBD 2019 data reported the global number of deaths, mortality rates and their temporal trends, by estimating the annual percent change over the period 1990–2019 (8). However, they did not report the incidence or DALYs due to LRIs and only reported the attributable burden of some etiologies and risk factors (8). Therefore, we reported the incidence, deaths and DALYs associated with LRIs, along with their attributable risk factors by age, sex, etiology and SDI in 204 countries and territories from 1990 to 2019.

## Methods

### Overview

The Global Burden of Disease (GBD) is a large international study which is updated annually and managed by the Institute of Health Metrics and Evaluation (IHME). GBD 2019 is the most recent iteration, in which the level and trends associated with 369 diseases and injuries and 87 risk factors were estimated for 204 countries and territories from 1990 to 2019. A detailed description of the GBD 2019 methodology can be found in previous articles (9, 10) and their estimates are available at https://vizhub.healthdata.org/gbd-compare/ and http://ghdx.healthdata.org/gbd-results-tool.

### Case definition and data sources

We provide a summary of the GBD methodology for LRIs. The case definitions for LRIs were clinician-diagnosed pneumonia or bronchiolitis. LRIs included diseases coded A48.1, J09-J22, J85.1, P23-P23.9, and U04 in the International Classification of Diseases (ICD) version 10 and 073.0–073.6, 079.82, 466–469, 480–489, 513.0, and 770.0 in ICD version 9 (9). The LRI etiology modeling was undertaken separately from the overall LRI incidence and prevalence. The etiologies consisted of *influenza, respiratory syncytial virus (RSV), Streptococcus pneumoniae*, and *Haemophilus influenzae* type *b (Hib)* which are types of LRI where the etiology is the causal pathogen in the infection (9).

The data used in GBD 2019 includes and updates GBD 2017, with more recent data obtained from the systematic review, newly identified surveys, as well as new claims and inpatient data. Two primary types of input data were used by IHME for LRIs. Firstly, LRI incidence and prevalence data, were extracted from a systematic literature review, data from hospital inpatient and outpatients, US claims data, and population-representative surveys. Secondly, data were obtained regarding the etiologies of LRI (9).

Population attributable fractions for influenza and RSV were obtained from a systematic review of the proportion of LRI cases that were positive for each pathogen. Furthermore, information about Hib and *Streptococcus pneumoniae* (pneumococcal pneumonia) were obtained via a systematic review of the literature on vaccine efficacy and effectiveness. The search strategies used for both the incidence and prevalence of LRI cases and the etiology for influenza and RSV are reported elsewhere (9). The inclusion criteria were: a minimum sample size of 100 participants, a study duration of at least 1 year and having case definitions of lower respiratory infections (i.e., pneumonia or bronchiolitis). One-hundred and twenty-one studies were identified, but only two met the inclusion criteria and were included. Studies which only reported pandemic-related H1N1 influenza and those with a case definition of influenza-like illness were excluded. If the ages of the study participants were not reported, age ranges were assigned based on the prevalence-weighted mean age of LRI in the appropriate year/sex/location (9).

A systematic review was also conducted by IHME, up to May 2017, on the effectiveness of the Hib and pneumococcal conjugate (PCV) vaccines, against pneumonia, which was confirmed by x-ray, and pneumococcal and Hib disease. For the PCV studies, where available IHME extracted the distribution of pneumococcal pneumonia serotypes and the serotypes included in the PCV used in that study, but no additional studies were identified. Observational and case-control studies for Hib were excluded, because the vaccine efficacy estimates were implausibly high. In addition, Hib trial data were solely for children <5 years, meaning that Hib could not be modeled for those older than 5 years old. Furthermore, the PCV trial data were also frequently limited to those in younger age groups. In order to identify the contribution of pneumococcal pneumonia in older age groups, PCV efficacy studies using a before-after approach were also included (9).

Non-fatal burden of LRI was estimated using self-reported prevalence of LRI symptoms identified in population-representative surveys, including the Demographic and Health Survey and the Multiple Indicator Cluster Survey. Survey data was extracted by one-year age group and by sex as far as possible. In addition, fortnightly prevalence was transformed into point prevalence with an adjustment formula (9). Four definitions were used to identify the prevalence of LRI using the following symptoms: (1) the *gold standard* was cough with difficulty breathing and symptoms in the chest, as well as a fever, but we also accepted (2) Cough and difficulty breathing with symptoms in the chest, but no fever, (3) Cough with difficulty breathing, with a fever, and (4) cough with difficulty breathing, but no fever. In order to allow comparability, the first definition was considered to be the reference definition, and the prevalence using the other case definitions were adjusted accordingly using survey crosswalk coefficients from a meta-regression. Seasonality of the survey data was also taken into account. In addition, the LRI modeling also included hospital inpatient and outpatient data, as well as US claims data. Prior to modeling, the data were adjusted for multiple admissions, multiple diagnoses, for outpatient claims, and their definitions were adjusted to our reference case definition. The claims data for GBD 2019 included Marketscan (US), as well as data from Taiwan, Poland, and Russia. These data were first converted into prevalence data, then compared to the LRI reference definition using a meta-regression model. Finally, the new sources were added to the data sources included in the previous GBD iteration, resulting in a total of 1,152 data sources from 162 countries being used to estimate the non-fatal burden of LRIs (9).

A Cause of Death Ensemble model (CODEm) was used to estimate LRI mortality separately for males and females, for children under 5 years and those older than five due to starkly different patterns. The CODEm included all data from available vital registration systems, surveillance systems, and verbal autopsies, with outliers checked and excluded by country or region. ICD9-coded mortality data in Sri Lanka (1982, 1987–1992), ICD9-coded neonatal mortality data in Guatemala (1980, 1981, 1984,2000–2004), and medically coded cause of death data and Civil Registration System data in many Indian states (1986–2013) were excluded. The etiologies of LRI were also updated using a systematic review (9).

### Disease modeling

DisMod-MR, a Bayesian meta-regression modeling framework, was used to model the non-fatal burden of LRI. This process produced estimates of the incidence, prevalence, and remission by age, sex, geographic location, and year. The recovery time was an average of 10 days (range: 5–15 days), which corresponds with a remission 36.5 days. The models also included country-level covariates (9).

The LRI fatal burden was modeled using CODEm with various country-level covariates. Due to large differences in the patterns, separate LRI CODEm models were made for those under 5 years old and those aged 5–95+ years old. As with all GBD mortality models LRI mortality models were single-cause, meaning that the sum of all of the mortality models must be the same as the all-cause mortality envelope. LRI mortality estimates, and other causes of mortality, were corrected by rescaling them in accordance with the uncertainty surrounding the cause-specific mortality rate. This process, which is called CoDCorrect, ensures internal consistency among the causes of death (9).

LRI etiologies were calculated using two distinct counterfactual modeling strategies to estimate the population attributable fractions (PAFs), which have previously been reported in detail (9). The PAF presents the relative reduction in LRI mortality if there was no exposure to a particular etiology. As LRIs can be caused by multiple pathogens, which may also co-infect, PAFs can overlap and may add up to more than 100%. Different strategies were utilized to estimate viral (influenza and RSV) and bacterial (*Streptococcus pneumoniae* and Hib) etiologies. Furthermore, etiologies for neonatal deaths were not attributed due to a lack of quality data. The uncertainty estimates for the PAF were calculated using 1,000 draws for each parameter using normal distributions in log space. A detailed description of the methodology for estimating the PAFs, as well as modeling the non-fatal and fatal burden of the etiologies, can be found elsewhere (9).

### Compilation of results

The GBD 2013 European Disability Weights Measurement Study was used to determine the disability weights (DWs) for all sequelae (11). Supplementary Table S1 presents the severity levels, lay descriptions, and associated DWs for each LRI outcome. The proportion in each severity category, moderate (85%) and severe (15%), were calculated using the results of a meta-analysis study, which were then multiplied by the severity-specific DWs to produce the years lived with disability (YLDs) (9).

The years of life lost (YLLs) were estimated by multiplying the number of deaths in an age group with the remaining life expectancy in that age group, using the GBD standard life table. The sum of the YLLs and YLDs were used to estimate the DALYs. Uncertainty was measured by sampling 1,000 draws at each computational step, combining uncertainty from multiple sources, including input data, corrections of measurement error and estimates of residual non-sampling error. Uncertainty intervals (UIs) were defined as the 25th and 975th values of the ordered draws. The relationship between the burden of LRI (i.e., DALYs) and the SDI for 21 regions and 204 countries and territories was investigated using Smoothing Splines models (12). The level of development was estimated using SDI (Social Development Index) which is a composite measure which included income per capita, education level among the population aged 15 years and older, and total fertility rate under the age of 25. The SDI ranges from 0 (less developed) to 1 (most developed). R software, version 3.5.2, was used to map the age-standardized incidence, deaths and DALY rates.

### Risk factors

Only risk factors which have substantial evidence for causing LRI were included in this study (10). The percentage of DALYs due to LRI that were attributable to child wasting (13), household air pollution from solid fuels, a lack of access to handwashing facilities (14), ambient particulate matter (15), low birth weight (LBW) (16), short gestation (16), child stunting (13), secondhand smoke (17), child underweight (13), smoking (16), high temperature (10), non-exclusive breastfeeding (18), low temperature (10) and alcohol use (19) were reported. The definitions of these risk factors, and their relative risk for LRI, are reported elsewhere (10).

## Results

### Global level

Globally, there were 488.9 million (95% UI: 457.6 to 522.6) incident cases of LRI in 2019, with an age-standardized incidence of 6,295.0 (5,887.4–6,737.3) per 100,000 population, which represents a 23.9% (22.5–25.4) decrease since 1990. LRI accounted for 2.4 million (2.3–2.7) deaths in 2019, with an age-standardized rate of 34.3 (31.1–37.9), which is 48.5% (42.9–54.0) lower than in 1990 (Table 1). In 2019, the number of DALYs due to LRI worldwide was 97.2 million (84.9 to 113.1), with an age-standardized rate of 1,386.1 (1,203.5–1,622.8) DALYs per 100,000 population, a decrease of 62.5% (54.9 to 69.0) since 1990 (Table 1).

**Table 1:** Incident cases, deaths and DALYs for lower respiratory infection in 2019 and percentage change in the age- standardised rates (ASRs) per 100,000, by GBD region, from 1990 to 2019 (generated from data available from http://ghdx.healthdata.org/gbd-results-tool).

	**Incidence (95% UI)**			**Deaths (95% UI)**			**DALYs (95% UI)**		
	**No. (95% UI)**	**ASRs per 100,000 (95% UI)**	**Percentage change in ASRs between 1990 and 2019**	**No. (95% UI)**	**ASRs per 100,000 (95% UI)**	**Percentage change in ASRs between 1990 and 2019**	**No. (95% UI)**	**ASRs per 100,000 (95% UI)**	**Percentage change in ASRs between 1990 and 2019**
Global	488902504 (457572987, 522635542)	6,295 (5,887.4, 6,737.3)	−23.9 (−25.4, −22.5)	2493,200 (2268,183, 2736,184)	34.3 (31.1, 37.9)	−48.5 (−54, −42.9)	97189708 (84871207, 113082891)	1,386.1 (1,203.5, 1,622.8)	−62.5 (−69, −54.9)
High–income Asia Pacific	5700982 (5328085, 6083720)	2,344.8 (2,101.7, 2,597.7)	−27.8 (−30, −25.4)	137989 (110299, 154096)	22.1 (18.3, 24.4)	−47.5 (−52.7, −44.2)	1499688 (1268092, 1638181)	305.3 (267.8, 327.1)	−53.9 (−57.4, −51.6)
High–income North America	14868900 (13950171, 15845711)	3,757.7 (3,471.9, 4,059.7)	−12.9 (−15.2, −10.2)	91060 (79893, 97147)	13.2 (11.7, 14)	−39.6 (−41.5, −37.8)	1339935 (1240713, 1397495)	239.7 (225.9, 248.3)	−40.7 (−42.3, −39.1)
Western Europe	16395485 (15264664, 17666073)	2,453.1 (2,259.8, 2,678.5)	−28.4 (−30, −26.8)	159075 (136430, 171243)	13.7 (11.9, 14.7)	−37.8 (−40.8, −35.2)	1802175 (1609136, 1909902)	192.7 (176, 202.7)	−45.4 (−47.9, −43.4)
Australasia	847915 (786727, 917942)	2,303.3 (2,113.4, 2,524.6)	−24.2 (−27.3, −20.8)	5164 (4271, 5780)	8.9 (7.5, 9.9)	−36.1 (−41.7, −30.9)	59638 (52027, 65001)	127.6 (113.6, 138.1)	−47.3 (−51.7, −43.3)
Andean Latin America	5400792 (5034520, 5784693)	9,264.7 (8,642.5, 9,952.4)	−29.6 (−32.8, −26.5)	30185 (24350, 36816)	55.6 (44.9, 67.5)	−49.8 (−59.2, −39.3)	784845 (624452, 977491)	1,326.9 (1,060.4, 1654)	−72.4 (−78.9, −65)
Tropical Latin America	19310825 (18046876, 20623868)	8,460.9 (7,903.8, 9,068.5)	−24.2 (−26.4, −22.1)	90114 (79540, 96039)	40.8 (35.8, 43.5)	−36.4 (−42.9, −31.2)	2033803 (1884705, 2176918)	956.6 (876.5, 1039.6)	−64.8 (−70.6, −59.8)
Central Latin America	13417558 (12537543, 14385830)	5,726.2 (5,340.9, 6,136.2)	−35.1 (−36.7, −33.6)	51851 (44387, 60174)	23 (19.7, 26.8)	−51.8 (−58.8, −44.1)	1642711 (1352551, 2011184)	717.4 (586.1, 882)	−64 (−71, −55.1)
Southern Latin America	3432983 (3186597, 3703188)	4,743.1 (4,394.3, 5,122)	−13 (−16.7, −9.2)	42564 (37971, 45844)	50.2 (45, 54.1)	31.1 (22.4, 40.1)	692430 (638457, 742965)	883.9 (816.9, 950.3)	−10.7 (−17.4, −3.3)
Caribbean	3118752 (2920790, 3345178)	6,395 (5,975.2, 6,872.2)	−25.1 (−27.4, −22.9)	19182 (16476, 22128)	39.3 (33.6, 45.6)	−31.3 (−41.7, −19.9)	621803 (508359, 753074)	1,418.8 (1,149.9, 1728.7)	−45.2 (−57.9, −29.6)
Central Europe	5132184 (4799948, 5523102)	3624.9 (3328.6, 3964.6)	−31.8 (−33.7, −30.1)	30679 (27109, 33943)	15.5 (13.7, 17.1)	−45.8 (−51.9, −39.8)	599841 (528609, 667838)	403.1 (354.3, 453.5)	−67.6 (−72.2, −62.9)
Eastern Europe	13294778 (12428403, 14225691)	5,232.6 (4,834.9, 5,662.9)	−14.2 (−16.3, −11.8)	44838 (40209, 49911)	15.6 (14, 17.2)	1 (−9.5, 11.9)	1423084 (1271089, 1588785)	581.2 (519.6, 643.4)	−24.4 (−33.1, −15.7)
Central Asia	4453344 (4193895, 4743352)	5,436.3 (5,133.8, 5,780.7)	−24.7 (−27.4, −22.3)	24430 (21283, 28467)	31.1 (27.5, 35.6)	−52.6 (−59.1, −44.9)	1507867 (1262441, 1821204)	1,668.9 (1405, 2008.9)	−66.5 (−72.5, −59.1)
North Africa and Middle East	34197034 (31709280, 36805894)	6,510.2 (6,063.6, 6,997.8)	−28.9 (−30.8, −26.8)	107742 (94479, 122048)	26.4 (23.2, 29.6)	−51.9 (−59.2, −45.2)	4716300 (3993317, 5473257)	888.5 (761.1, 1019.9)	−71.4 (−77.8, −65.1)
South Asia	174927531 (163067794, 187409925)	10,998.7 (10,265.9, 11,794.2)	−21.7 (−23.5, −19.9)	548077 (486877, 615225)	42.4 (37.8, 47.8)	−50.4 (−56.7, −43.3)	25525882 (22096613, 29761535)	1,652.7 (1,444.1, 1917)	−64.9 (−71.1, −57.1)
Southeast Asia	41981067 (39209801, 44943426)	6,822.8 (6,382.8, 7,328.4)	−22.4 (−24.5, −20)	219724 (180485, 245478)	45.1 (36.6, 50.4)	−35.6 (−47.3, −25)	6925100 (6023294, 7827538)	1,246.9 (1,080.2, 1414)	−65.4 (−72.5, −57.3)
East Asia	59443625 (55161107, 63906555)	3,890.1 (3,564, 4,198.1)	−44.3 (−46.6, −42.3)	205330 (180262, 238102)	14.6 (12.9, 16.8)	−76.2 (−79.5, −69.3)	4372837 (3904840, 4951861)	355.3 (316.7, 404.3)	−89.6 (−91.4, −86.8)
Oceania	890935 (810411, 978441)	7,737.6 (7,170.1, 8,360.6)	−19.7 (−22.2, −17.1)	7771 (5889, 10105)	69.8 (55.7, 91.7)	−28 (−41.4, −11.9)	546681 (406465, 721967)	3,353.4 (2,528.9, 4,353)	−32.2 (−48.4, −11.6)
Western Sub–Saharan Africa	30715183 (28374576, 33283614)	9,378.5 (8,770.2, 10,023.3)	−19.5 (−21, −18)	353643 (289548, 424330)	106.3 (91.4, 122.4)	−32.2 (−42.2, −20.7)	23847090 (18927582, 29481882)	4,198.1 (3,432.3, 5,041.9)	−47.2 (−58.6, −32)
Eastern Sub–Saharan Africa	25993854 (24041397, 28307585)	8,453.3 (7,929.8, 9,020.5)	−30.8 (−32.1, −29.4)	210094 (180426, 248234)	95.7 (85.2, 106.9)	−44.5 (−51.3, −37)	11897920 (9658892, 14747300)	2,908.2 (2,509.2, 3,423.6)	−63 (−69.9, −54.3)
Central Sub–Saharan Africa	8867737 (8139554, 9680999)	9,459.2 (8,762.8, 10,213.9)	−33.1 (−35.7, −30.4)	66199 (52715, 81678)	107.7 (85.6, 139.1)	−38.7 (−49.4, −26.2)	3388970 (2622033, 4286609)	2,964.6 (2,364.4, 3,664.5)	−60 (−69, −49.1)
Southern Sub–Saharan Africa	6511041 (6081425, 6958181)	9,769.2 (9,146.1, 10,445.3)	−12.8 (−14.9, −10.7)	47489 (42559, 52794)	85.4 (77.1, 93.7)	−16.7 (−25.2, −8.1)	1961107 (1693462, 2282721)	2,779 (2,435.9, 3,193.4)	−36.1 (−44.9, −24.8)

### Regional level

In 2019, the age-standardized incidence of LRIs were highest in South Asia [10,998.7 (95% UI: 10,265.9–11,794.2)], Southern Sub-Saharan Africa [9,769.2 (95% UI: 9,146.1–10,445.3)] and Central Sub-Saharan Africa [9,459.2 (95% UI: 8,762.8–10,213.9)]. Australasia [2,303.3 (95% UI: 2,113.4–2,524.6)], High-income Asia Pacific [2,344.8 (95% UI: 2,101.7–2,597.7)] and Western Europe [2,453.1 (95% UI: 2,259.8–2,678.5)] had the lowest age-standardized rates (Table 1). Central Sub-Saharan Africa [107.7 (95% UI: 85.6–139.1)], Western Sub-Saharan Africa [106.3 (95% UI: 91.4–122.5)] and Eastern Sub-Saharan Africa [95.7 (95% UI: 85.2–106.9)] had the highest age-standardized death rates from LRIs. In contrast, these rates were lowest for Australasia [8.9 (95% UI: 7.5–9.9)], High-income North America [13.2 (95% UI: 11.7–14.0)] and Western Europe [13.7 (95% UI: 11.9–14.7)] (Table 1). Western Sub-Saharan Africa [4,198.1 (95% UI: 3,432.3–5,041.9)], Oceania [3,353.4 (95% UI: 2,528.9–4,353.0)], and Central Sub-Saharan Africa [2,964.6 (95% UI: 2,364.4–3,664.5)] had the highest age-standardized DALY rates from LRIs in 2019. In contrast, Australasia [127.6 (95% UI: 113.6–138.1)], Western Europe [192.7 (95% UI: 176.0–202.7)] and High-income North America [239.7 (95% UI: 225.9–248.3)] had the lowest age-standardized DALY rates (Table 1). The age-standardized incidence, deaths and DALY rates of LRI in 2019, for all GBD regions, are presented in Supplementary Figures S1–S3, respectively.

Most regions saw decreases in the age-standardized incidence, deaths and DALY rates of LRIs over the last 30 years. East Asia [−44.3% (95% UI: −46.6 to −42.3)], Central Latin America [−35.1% (95% UI: −36.7 to −33.6)] and Central Sub–Saharan Africa [−33.1% (95% UI: −35.7 to −30.4)] experienced the largest decreases over the duration of this study (Table 1). In the same period, decreases in the age–standardized death rates from LRIs were observed in East Asia [−76.2% (95% UI: −79.5 to −69.3)], Central Asia [−52.6% (95% UI: −59.1 to −44.9)] and North Africa and the Middle East [−51.9% (95% UI: −59.2 to −45.2)]. In contrast, Southern Latin America [31.1% (95% UI: 22.4 to 40.1)] was the only region in which an increase in the age–standardized death rate was seen (Table 1). In addition, East Asia [−89.6% (95% UI: −91.4 to −86.8)], Andean Latin America [−72.4% (95% UI: −78.9 to −65.0)] and North Africa and the Middle East [−71.4% (95% UI: −77.8 to −65.1)] had the largest decreases in the age–standardized DALY rates of LRIs from 1990 to 2019 (Table 1). The percentage change, from 1990 to 2019, in the age–standardized incidence, death and DALY rates for LRIs are presented in Supplementary Figures S4–S6, respectively.

The number of incident cases of LRIs increased from 414.3 million (95% UI: 383.5 to 449.1) in 1990 to 488.9 million (95% UI: 457.6 to 522.6) in 2019, with South Asia, East Asia and Southeast Asia having the largest number of incident cases in 2019 (Supplementary Figure S7; Supplementary Table S2). In contrast, the number of deaths due to LRIs decreased from 3.3 million (95% UI: 3.0–3.7) in 1990 to 2.5 million (95% UI: 2.3–2.7) in 2019, with South Asia, Western Sub-Saharan Africa and Southeast Asia having the highest number of deaths due to LRIs in 2019 (Supplementary Figure S8; Supplementary Table S3).

### National level

**Figure 1:**
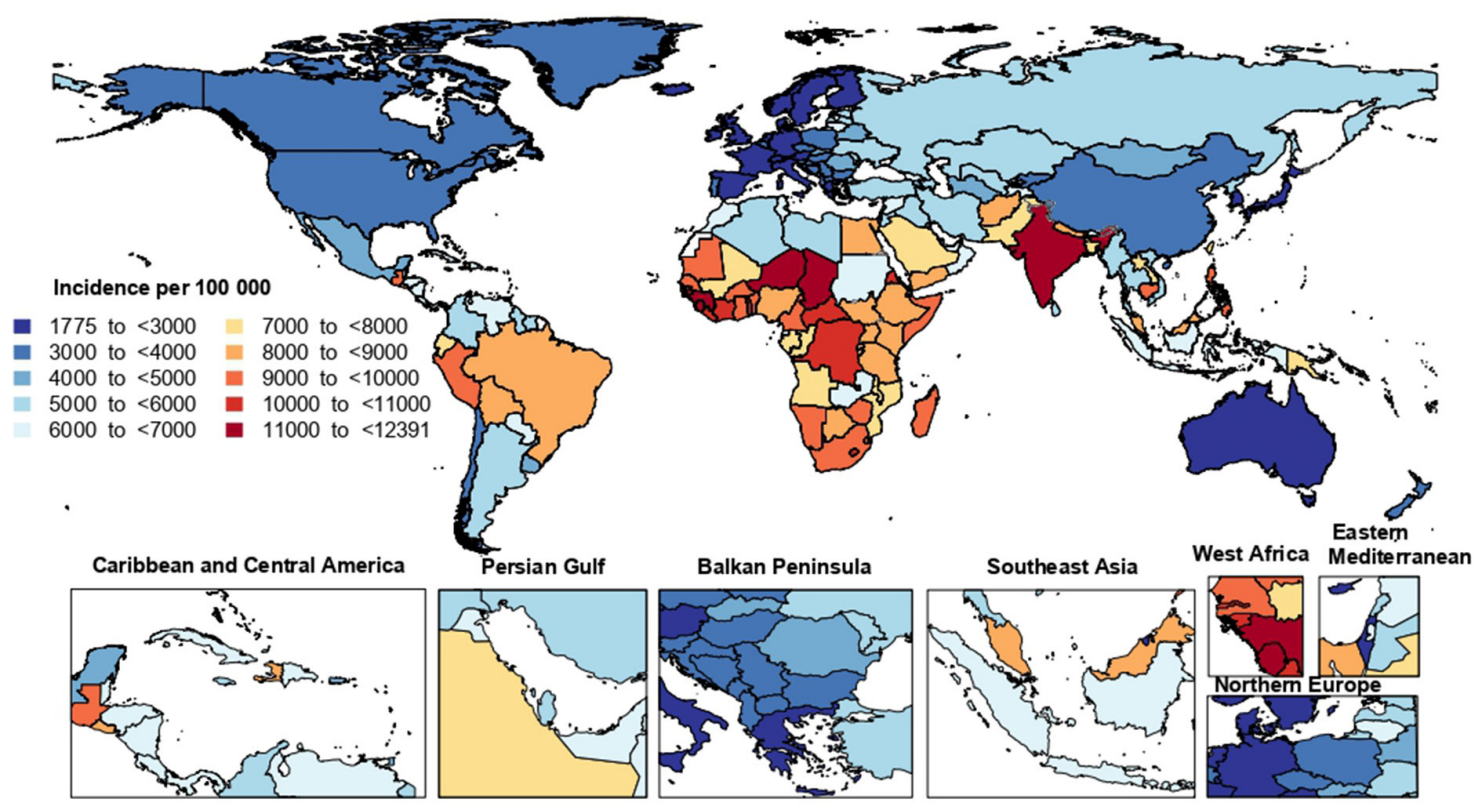
Age-standardized incidence rate of lower respiratory infections (per 100,000 population) in 2019, by country (generated from data available from http://ghdx.healthdata.org/gbd-results-tool).

In 2019, the national age-standardized incidence of LRIs ranged from 1,775.9 to 12,390.4 cases per 100,000 population. In 2019, Guinea [12,390.4 (95% UI: 11,495.5–13,332.8)], Chad [12,208.1 (95% UI: 11,289.3–13,202.5)] and India [11,862.1 (95% UI: 11,087.0–12,749.0)] had the three highest age-standardized incidence rates of LRIs. In contrast, Switzerland [1,775.9 (95% UI: 1,607.0–1,952.0)], Cyprus [1,983.2 (95% UI: 1,803.8–2,183.7)] and the Republic of Korea [2,036.4 (95% UI: 1,799.4–2,292.8)] had the lowest rates (Figure 1; Supplementary Table S2).

**Figure 2:**
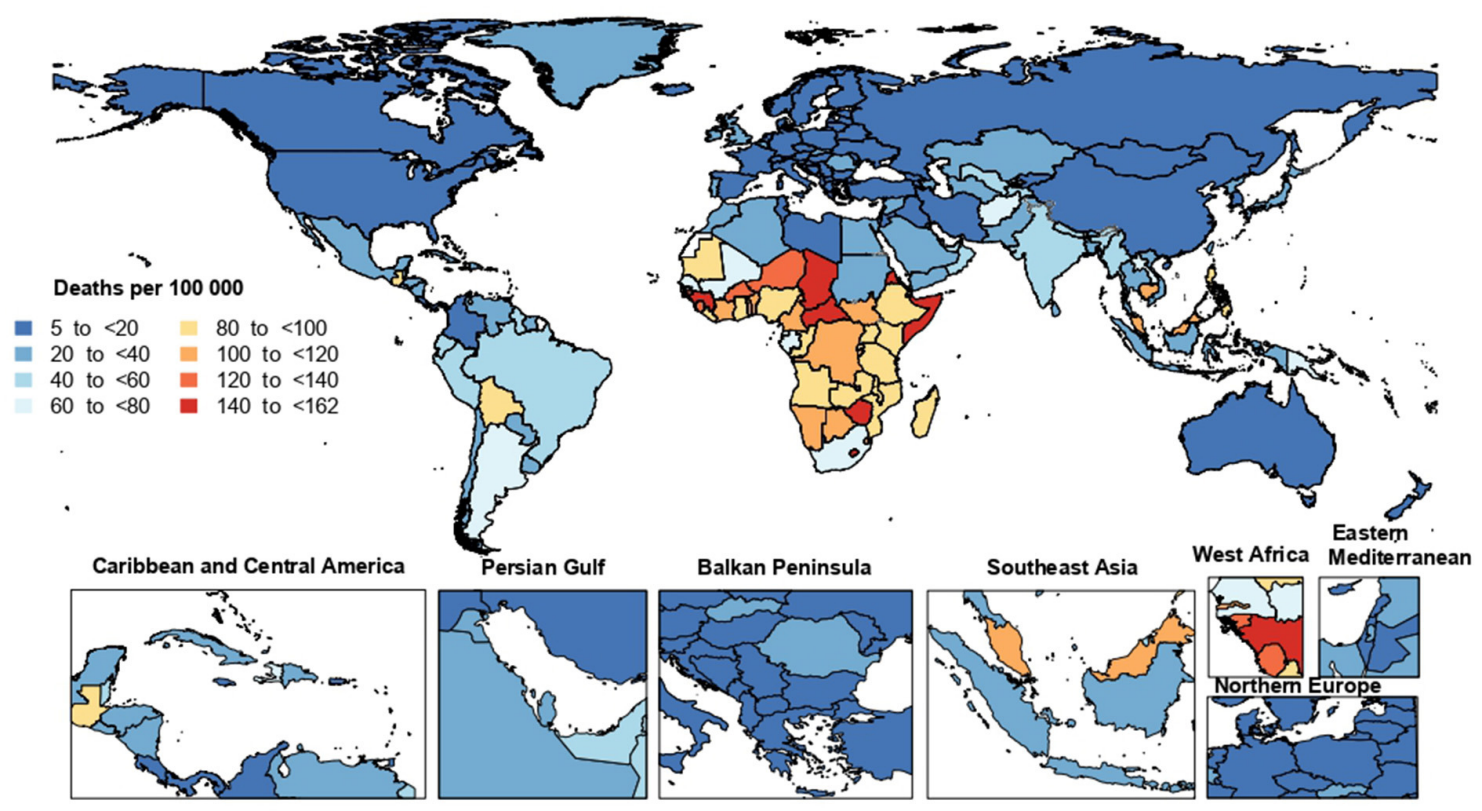
Age-standardized death rate from lower respiratory infections (per 100,000) population in 2019, by country (generated from data available from http://ghdx.healthdata.org/gbd-results-tool).

The national age-standardized death rates of LRI in 2019 varied from 5.2 to 161.5 cases per 100,000 population. The highest rates were observed in the Central African Republic [161.5 (95% UI: 122.4–213.4)], Zimbabwe [153.9 (95% UI: 117.4–186.7)] and Guinea [152.8 (95% UI: 120.1–191.7)], with lowest rates being found in Finland [5.2 (95% UI: 4.5–5.8)], Austria [5.6 (95% UI: 4.8–6.1)] and Croatia [6.5 (95% UI: 5.3–8.0)] (Figure 2; Supplementary Table S3).

In 2019, the national age-standardized DALY rates of LRI ranged from 79.1 to 6,145.4 cases per 100,000 population. The highest rates were observed in the Central African Republic [6,145.4 (95% UI: 4,444.0–8,428.8)], Chad [5,716.6 (95% UI: 4,534.0–7,167.4)] and Somalia [5,596.5 (95% UI: 4,096.1–7,508.7)]. Conversely, the lowest rates were seen in Finland [79.1 (95% UI: 71.7–86.5)], Austria [88.5 (95% UI: 80.4–96.0)] and Italy [98.7 (95% UI: 89.5–104.1)] (Supplementary Figure S9; Supplementary Table S4).

There were substantial differences between countries for the percentage change in the age-standardized incidence from 1990 to 2019, with Equatorial Guinea [−52.7% (95% UI: −55.8 to −49.3)], Chile [−50.2% (95% UI: −53.4 to −47.0)] and Albania [−48.6% (95% UI: −51.7 to −45.3)] showing the largest decreases during the measurement period. In contrast, Argentina [11.5% (95% UI: 5.4 to 18.1)] was the only country that showed an increase in the age–standardized incidence during the study duration (Supplementary Table S2).

Finland [−86.4% (95% UI: −87.7 to −85)], Mongolia [−83.1% (95% UI: −86.9 to −75.5)] and Albania [−81.3% (95% UI: −85.4 to −67.4)] showed the largest decreases in the age–standardized death rates from LRIs during the measurement period. In contrast, Argentina [120.5% (95% UI: 104.0 to 137.5)], Greece [34.9% (95% UI: 22.3 to 48.1)], Ukraine [30.0% (95% UI: 8.4 to 56.6)] and Kuwait [21.4% (95% UI: 1.2 to 44.6)] were the four countries which had an increase in the age–standardized death rates over the last 30 years (Supplementary Table S3).

China [−90.1% (95% UI: −91.8 to −87.3)], Mongolia [−87.9% (95% UI: −91.1 to −82.8)] and Turkey [−87.5% (95% UI: −91.2 to −83.1)] showed the largest decreases in the age-standardized DALY rates of LRIs during the measurement period. In contrast, Argentina [42.4% (95% UI: 30.4 to 56.3)] was the only country in which an increase in the age-standardized DALY rate was observed during the same period (Supplementary Table S4).

### Age and sex patterns

**Figure 3:**
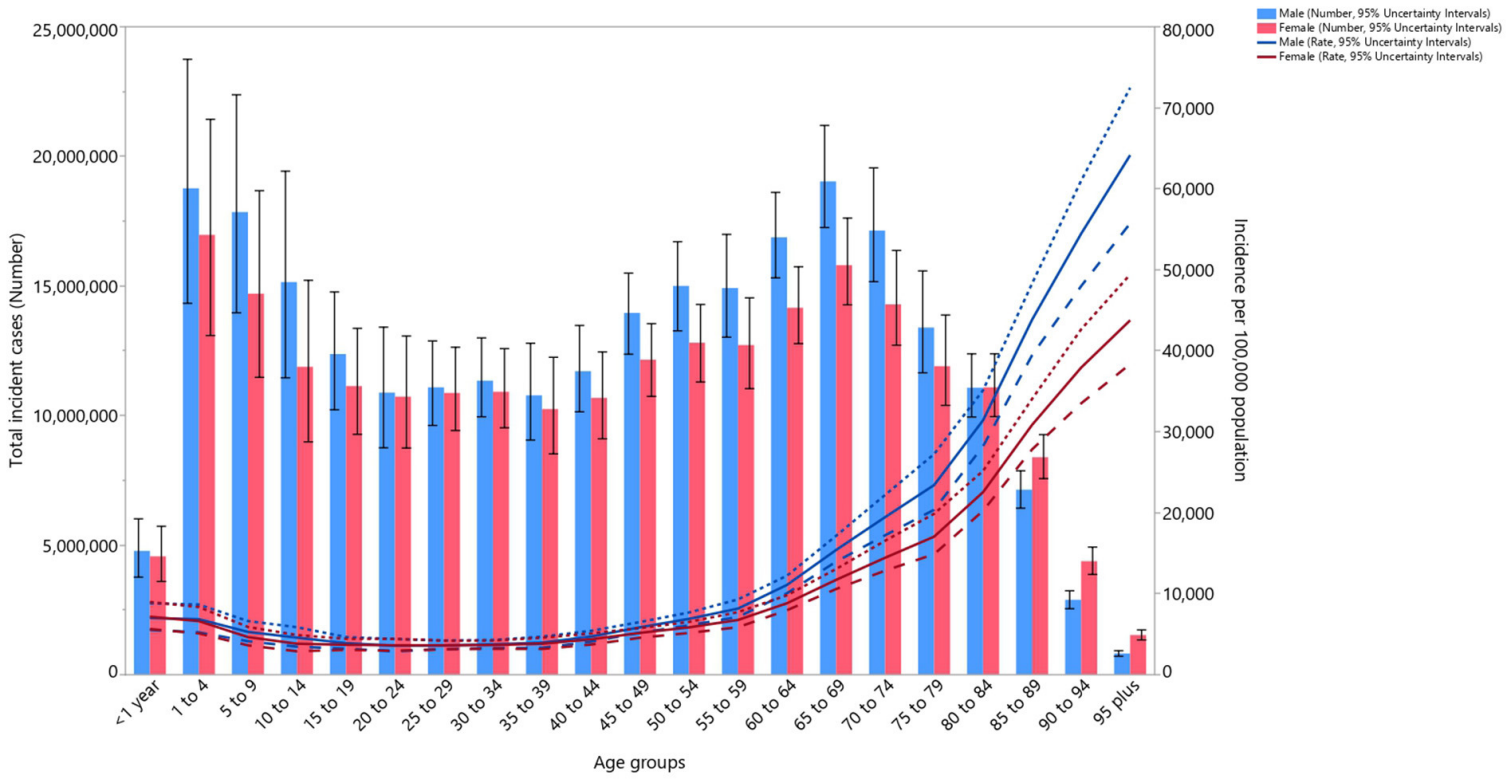
Global number of incident cases and incidence rate of lower respiratory infections (per 100,000 population) by age and sex in 2019; Dotted and dashed lines indicate 95% upper and lower uncertainty intervals, respectively (generated from data available from http://ghdx.healthdata.org/gbd-results-tool).

In 2019, globally there was a decreasing trend for LRI incidence rate up to the 25–29 years age group, and then the incidence rates increased with age. In addition, the number of incident cases reached its highest in the 65–69 and 1–4 years age groups for males and the same age groups for females (Figure 3). In 2019, the global death rate of LRI was slightly higher in males, peaking in those aged 95^+^ years. The number of deaths reached its highest in the <1-year age group, after which there was a decline with increasing age up to the 15–19 years age group and then increasing up to the 80–84 age group (Supplementary Figure S10). The global DALY rate of LRI was slightly higher in males and peaked in the <1-year age groups and then decreased with age up to the 15–19 age group before again increasing up to the 80–84 age group. Similarly, the number of DALYs peaked in the <1-year age group and then decreased with increasing age, before increasing in the older age groups (Supplementary Figure S11).

### Association with the Socio-demographic Index

**Figure 4:**
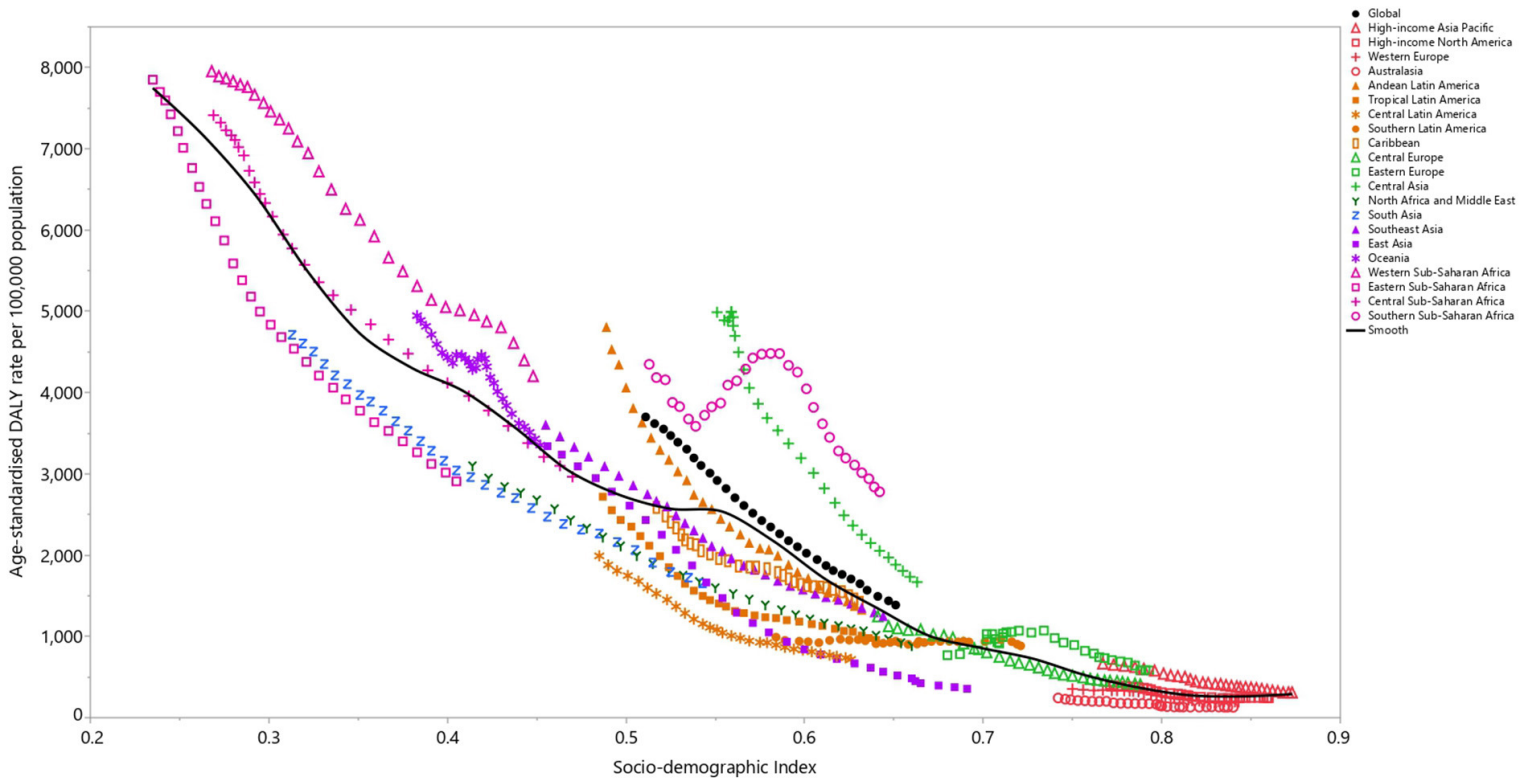
Age-standardized DALY rates of lower respiratory infections for the 21 Global Burden of Disease regions by Socio-demographic Index, 1990–2019; Expected values based on Socio-demographic Index and disease rates in all locations are shown as the black line. Thirty points are plotted for each GBD region and show the observed age-standardized DALY rates from 1990 to 2019 for that region. DALY, disability adjusted life years (generated from data available from http://ghdx.healthdata.org/gbd-results-tool).

At the regional level there was a negative association between the SDI and the age-standardized DALY rate of LRI, suggesting that the burden of LRIs decreased with increasing SDI. South Asia, North Africa and the Middle East, Tropical Latin America, Central Latin America, Australasia and Western Europe all had lower than expected DALY rates, from 1990 to 2019, based upon their level of socio-demographic development (as measured by the SDI). In contrast, Western Sub-Saharan Africa, Southern Sub-Saharan Africa, Central Asia, and Oceania had higher than expected burdens during this period (Figure 4).

At a country-level, in 2019, the burden of LRIs decreased sharply with increasing socio-economic development. Countries and territories, such as the Central African Republic, Chad, Guinea, Lesotho, Zimbabwe, Nigeria, Eswatini, Botswana, Palau and Malaysia had much higher than expected burdens, whereas countries and territories such as Mali, Liberia, Nepal, Yemen, Bhutan, Sudan and the Maldives had much lower than expected burdens (Supplementary Figure S12).

### LRI etiologies

**Figure 5:**
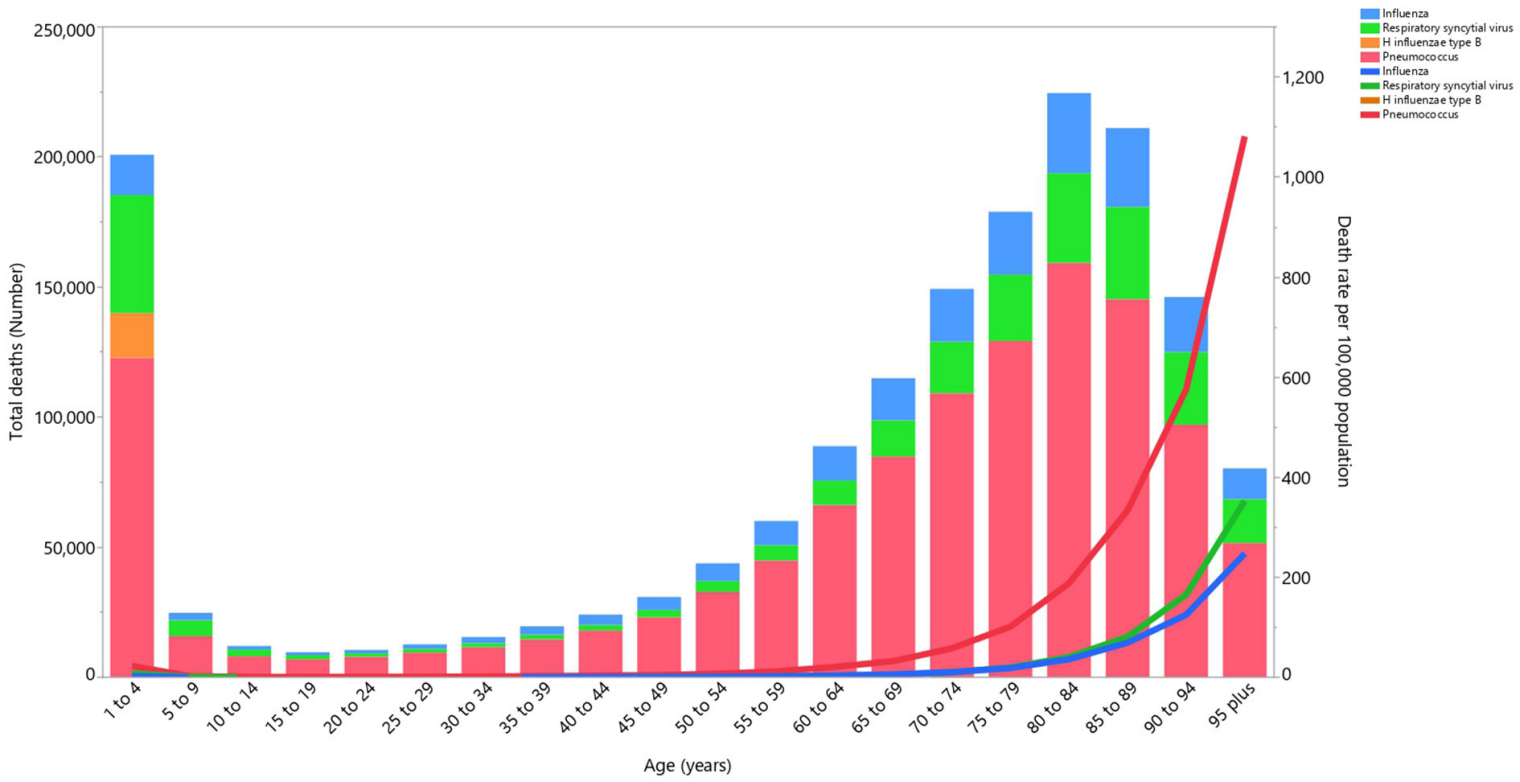
Global number of deaths and death rate due to lower respiratory infections (per 100,000 population) by etiology and age in 2019 (generated from data available from http://ghdx.healthdata.org/gbd-results-tool).

Although the proportion of LRI deaths attributable to the individual etiologies differed by age group, globally *Streptococcus pneumoniae* (*Pneumococcus*) accounted for most of the deaths across all age groups and the number of LRI deaths attributable to *Pneumococcus* were highest in the 80–84, 85–89 and 75–79 age groups, respectively (Figure 5). In addition, the death rate of LRIs attributable to all etiologies was highest in the 1–4 age group and then decreased up to the 45–49 age group, before increasing up to the oldest (95^+^) age group (Figure 5). Globally *Streptococcus pneumoniae* (*Pneumococcus*) accounted for most of the DALYs due to LRIs across all age groups and its contribution was highest in the 1–4 years age group (Supplementary Figure S13). In addition, the DALY rate of LRIs attributable to the four aforementioned etiologies showed an increase in the early age groups and then an increase was observed up to the oldest (95^+^) age groups (Supplementary Figure S13).

### Risk factors

**Figure 6:**
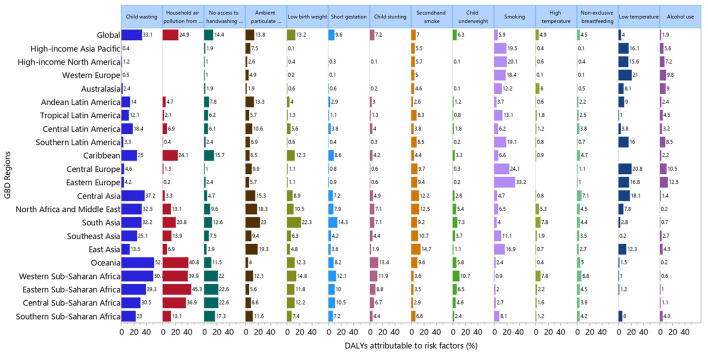
Percentage of DALYs due to lower respiratory infections attributable to risk factors for 21 GBD regions in 2019. DALY, disability adjusted life years (generated from data available from http://ghdx.healthdata.org/gbd-results-tool).

Although the proportion of DALYs attributable to the individual risk factors differed across the GBD regions, globally child wasting [33.1%], household air pollution from solid fuels [24.9%] and a lack of access to handwashing facilities [14.4%] had the three highest proportions of attributable DALYs (Figure 6). For males, child wasting [30.8%], household air pollution from solid fuels [24.0%] and a lack of access to handwashing facilities [14.2%] had the highest proportions of attributable DALYs. Similarly, the three main contributors for females were: child wasting [35.6%], household air pollution from solid fuels [26.0%] and a lack of access to handwashing facilities [14.7%] (Supplementary Figures S14, S15).

The proportion of DALYs attributable to the individual risk factors also varied by age group, with particularly large differences being observed for household air pollution from solid fuels and a lack of access to handwashing facilities. The highest proportions of attributable DALYs were in the 1–4 age group for household air pollution from solid fuels (34.7%) and a lack of access to handwashing facilities (18.4%) (Supplementary Figure S16). Similarly, for males and females, the proportion of DALYs attributable to household air pollution from solid fuels and a lack of access to handwashing facilities were highest in the 1–4 years age group (Supplementary Figures S17, S18).

## Discussion

GBD 2019 estimates indicate that the burden of LRIs globally and in all regions decreased over the period 1990–2019, except for an increase of 31 and 1% in the death rates in Southern Latin America and Eastern Europe, respectively. The Sub-Saharan African regions were among the countries with the largest incidence and death rates. Lower socioeconomic status, age <10 and >60 years and being male were associated with a higher burden of LRIs. Moreover, risk factors with the greatest attributable DALYs, like child wasting, were more common in the Sub-Saharan Africa, Oceania and Caribbean regions.

Based on the epidemiological transition theory, improvements in public health programs and practice and the development of novel preventative diagnostic and therapeutic tools for infectious diseases, which were initiated in the late 19th century, led to a steep decrease in the burden of infectious diseases by the mid-20th century (20). In accordance with these changes, our findings show a decline in the incidence, mortality and DALYs of LRIs over the last 30 years. Moreover, another study using GBD 2019 data reported that globally there was an average annual decrease of 2.39% in the age-standardized death rate attributable to LRIs over the period 1990–2019 (8). In addition, decreases in the incidence and deaths attributable to LRIs were observed at the global level for both sexes, and in almost all age groups, over the period 1990–2019 (7). The Sub-Saharan African regions were among the top five regions with the highest age-standardized burden in 2019. In line with our findings, a study by Seidu and colleagues on 13,495 women and children from 28 African countries found an acute LRI prevalence of 25.3% (21). This article also suggested that employed mothers (adjusted odds ratio (aOR): 0.77; 95% CI: 0.64–0.94) and improved toilet facilities (aOR: 0.72; 95% CI: 0.64–0.97), treatment for intestinal parasites and health education and promotion could be helpful in reducing acute LRIs (21). Similar to our findings showing that *Streptococcus pneumoniae* was the leading LRI etiology, in 2017 *S. pneumoniae* caused the highest number of LRIs in Africa (22). Therefore, expanding the coverage of vaccines, such as pneumococcal conjugate vaccines, could prevent a large number of deaths (22).

Lower socioeconomic status and a lack of social security are associated with higher incidence rates of most infectious diseases, such as acute respiratory infections and meningitis (23–25). In addition, an article by Kang et al. showed that in 2019 countries with the lowest SDIs had the heaviest LRI burden (8). Furthermore, there was a lower vaccine coverage for *S. pneumoniae* and *Haemophilus influenzae* type b vaccines, which are among the most common etiologies of LRIs in lower SDI countries, especially in Africa and South Asia (26). Taken together, these factors may have caused the higher burden of LRIs in low SDI countries, as shown in previous research (1, 3).

The age-standardized DALY and death rates were highest in children under-five years old and adults aged 70 and above, respectively. The higher susceptibility of children to LRIs is due to immune activation and the production of several cytokines, like tumor necrosis factor alpha (TNF-α) and interleukin-(IL-5), which lead to a higher burden in this age group (27). On the other hand, the higher frequency of underlying conditions in the elderly results in more deaths among older adults. Consequently, due to the consequences of LRIs on the health status of children, early diagnosis, effective treatment and a focus on preventive programs are needed (28).

The results of our research showed that child wasting, household air pollution from solid fuels and a lack of access to handwashing facilities made substantial contributions to the burden of LRIs in Africa, Oceania, Asia and Caribbean in 2019. In contrast, smoking, low temperature and alcohol use made considerable contributions to the burden of LRIs in Europe, Australasia, Latin America, High-income Asia Pacific and High-income North America. In accordance with our results, a systematic review revealed that low birth weight (OR: 3.18; 95% CI: 1.02–9.90), lack of exclusive breastfeeding (OR: 2.34; 95% CI: 1.42–3.88) and exposure to indoor air pollution (OR: 1.57 95% CI: 1.06–2.31) were associated with higher odds of severe acute LRIs in children (29). Moreover, another meta-analysis on mortality risk factors for acute LRIs in low- and middle-income countries identified other risk factors, such as being younger than 2 months old (OR: 5.22; 95% CI: 1.70–16.03), chronic underlying diseases (OR: 4.76; 95% CI: 3.27– 6.93), severe malnutrition (OR: 4.27; 95% CI: 3.47–5.25), young maternal age (OR: 1.84; 95% CI: 1.03–3.31), low maternal education (OR: 1.43; 95% CI: 1.13–1.82), second-hand smoke exposure (OR: 1.52; 95% CI: 1.20–1.93) and indoor air pollution (OR: 3.02; 95% CI: 2.11–4.31) (30). Generally, improving primary health care interventions and management (e.g., expanding vaccine coverage, providing vitamin A supplements and appropriate antenatal practices), antenatal education programs, encouraging breast feeding, socioeconomic and environmental modifications (e.g., improving access to handwashing facilities, decreasing the ambient and household air pollution and increasing incomes) and behavioral risk factor interventions (e.g., smoking cessation campaigns and reducing alcohol consumption) could be some recommended preventive strategies and measures for reducing the burden of LRIs (24, 30).

### Strengths and limitations of the study

The present study provides the most recent and up-to-date information on the burden of LRIs and their attributable risk factors at the global, regional and national levels. A consistent and reproducible approach was used in GBD 2019 to estimate the burden of LRIs due to influenza and RSV and to make inpatient, non-PCR, and single etiology studies more similar to the reference definition. In addition, in previous GBD studies, the IHME used a single study of the odds ratio for influenza and RSV in children younger than 5 years old and applied that to all ages. With a recently published article on the odds for these pathogens in adults over 65 years, IHME was able to have different values by age and produced more accurate LRI estimates by etiology. Nevertheless, we acknowledge that our study has some limitations. Firstly, there was sparse data in some locations, especially in Sub-Saharan Africa, which will affect the interpretation of our results. Secondly, a clinician-diagnosis of LRIs was included in this study without any further laboratory or imaging assessment, which might result in a high degree of variance and recall bias. Thirdly, our study is also limited by reporting the severity of LRIs which are associated with the mortality and morbidity, although the effects of severity were adjusted in the models. Fourthly, variations in the vaccine coverage and utilized treatments could interfere with the estimated burden of LRIs. In order to reduce this issue, the model was adjusted to account for vaccine coverage and expected vaccine performance. Finally, the general limitations of the GBD 2019 project, which have been mentioned elsewhere, are also applicable to the present study (9, 10).

## Conclusions

The mortality and morbidity associated with LRIs has decreased globally and in almost all regions between 1990 and 2019, mostly as a result of health promotion and improvements in health-related behaviors. However, LRIs are responsible for a high incidence and large number of deaths, particularly among under 5-year-olds and older adults. Evidence from this study suggests that child wasting, household air pollution from solid fuels and a lack of access to handwashing facilities were prominent contributors to the burden of LRIs. Therefore, health authorities and policy makers should consider implementing measures, such as improving handwashing facilities, especially in Sub-Saharan Africa. Nevertheless, further studies are needed to assess the healthcare interventions and programs to control LRIs and other potential risk factors.

## Author's note

This study is based on publicly available data and solely reflects the opinion of its authors and not that of the Institute for Health Metrics and Evaluation.

## Data availability statement

Publicly available datasets were analyzed in this study. This data can be found here: http://ghdx.healthdata.org/gbd-results-tool.

## Ethics statement

The present study was reviewed and approved by Ethics Committee of Shahid Beheshti University of Medical Sciences, Tehran, Iran (IR.SBMU.RETECH.REC.1400.537).

## Author contributions

SS, A-AK, and MA designed the study. SS and MAM analyzed the data and performed the statistical analyses. SS, AM, SAN, MJMS, GSC, KA, and JSK drafted the initial manuscript. All authors reviewed the drafted manuscript for critical content and final version of the manuscript.

## Supplementary material

Figure S1 The age-standardised incidence of lower respiratory infections in 2019 for the 21 Global Burden of Disease regions by sex (generated from data available from http://ghdx.healthdata.org/gbd-results-tool).

Figure S2 The age-standardised death rates of lower respiratory infections in 2019 for the 21 Global Burden of Disease regions by sex (generated from data available from http://ghdx.healthdata.org/gbd-results-tool).

Figure S3 The age-standardised DALY rates of lower respiratory infections in 2019 for the 21 Global Burden of Disease regions by sex DALY=disability adjusted life years (generated from data available from http://ghdx.healthdata.org/gbd-results-tool).

Figure S4 The percentage change in the age-standardised incidence rate of lower respiratory infections from 1990 to 2019 for the 21 Global Burden of Disease regions by sex (generated from data available from http://ghdx.healthdata.org/gbd-results-tool).

Figure S5 The percentage change in the age-standardised death rates of lower respiratory infections from 1990 to 2019 for the 21 Global Burden of Disease regions by sex (generated from data available from http://ghdx.healthdata.org/gbd-results-tool).

Figure S6 The percentage change in the age-standardised DALY rates of lower respiratory infections from 1990 to 2019 for the 21 Global Burden of Disease regions by sex. DALY=disability adjusted life years (generated from data available from http://ghdx.healthdata.org/gbd-results-tool).

Figure S7 Number of incident cases of lower respiratory infections from 1990 to 2019 for the 21 Global Burden of Disease regions (generated from data available from http://ghdx.healthdata.org/gbd-results-tool).

Figure S8 Number of deaths due to lower respiratory infections from 1990 to 2019 for the 21 Global Burden of Disease regions (generated from data available from http://ghdx.healthdata.org/gbd-results-tool).

Figure S9 Age-standardised DALY rate of lower respiratory infections (per 100,000 population) in 2019, by country. DALY=disability adjusted life years (generated from data available from http://ghdx.healthdata.org/gbd-results-tool).

Figure S10 Global number of deaths and the death rate from lower respiratory infections (per 100,000 population) in 2019, by age and sex; Dotted and dashed lines indicate 95% upper and lower uncertainty intervals, respectively (generated from data available from http://ghdx.healthdata.org/gbd-results-tool).

Figure S11 Global number of DALYs and the DALY rate from lower respiratory infections (per 100,000 population) in 2019, by age and sex; Dotted and dashed lines indicate 95% upper and lower uncertainty intervals, respectively. DALY=disability adjusted life years (generated from data available from http://ghdx.healthdata.org/gbd-results-tool).

Figure S12 Age-standardised DALY rates of lower respiratory infections for 204 countries and territories in 2019, by SDI; Expected values based on the Socio-demographic Index and disease rates in all locations are shown as the black line. Each point shows the observed age-standardised DALY rate for each country in 2019. DALY=disability adjusted life years. SDI, Socio-demographic Index (generated from data available from http://ghdx.healthdata.org/gbd-results-tool).

Figure S13 Global number of DALY and the DALY rate due to lower respiratory infections (per 100,000 population) in 2019, by aetiology and age. DALY=disability adjusted life years (generated from data available from http://ghdx.healthdata.org/gbd-results-tool).

Figure S14 Percentage of DALYs due to lower respiratory infections attributable to risk factors for the 21 GBD regions in 2019, for males. DALY=disability adjusted life years (generated from data available from http://ghdx.healthdata.org/gbd-results-tool).

Figure S15 Percentage of DALYs due to lower respiratory infections attributable to risk factors for the 21 GBD regions in 2019, for females. DALY=disability adjusted life years (generated from data available from http://ghdx.healthdata.org/gbd-results-tool).

Figure S16 Percentage of DALYs due to lower respiratory infections attributable to each risk factor in 2019, by age. DALY=disability adjusted life years (generated from data available from http://ghdx.healthdata.org/gbd-results-tool).

Figure S17 Percentage of DALYs due to lower respiratory infections attributable to each risk factor by age, for males, in 2019. DALY=disability adjusted life years (generated from data available from http://ghdx.healthdata.org/gbd-results-tool).

Figure S18 Percentage of DALYs due to lower respiratory infection attributable to each risk factor by age, for females, in 2019. DALY, disability adjusted life years (generated from data available from http://ghdx.healthdata.org/gbd-results-tool).

Table S1 Sequelae for lower respiratory infections and their corresponding disability weights in the GBD 2019 study.

Table S2 Incident cases of lower respiratory infections in 1990 and 2019 and the percentage change in the age-standardised rates (ASRs) per 100,000, by location (generated from data available from http://ghdx.healthdata.org/gbd-results-tool).

Table S3 Deaths from lower respiratory infections in 1990 and 2019 and the percentage change in the age-standardised rates (ASRs) per 100,000, by location (generated from data available from http://ghdx.healthdata.org/gbd-results-tool).

Table S4 DALYs from lower respiratory infections in 1990 and 2019 and the percentage change in the age-standardised rates (ASRs) per 100,000, by location (generated from data available from http://ghdx.healthdata.org/gbd-results-tool).
