# Supplementary figures and images for: Global burden of lower respiratory infections during the last three decades
Source: Front Public Health. 2023 Jan 9;10:1028525. doi: 10.3389/fpubh.2022.1028525 (PMC9869262; doi:10.3389/fpubh.2022.1028525)

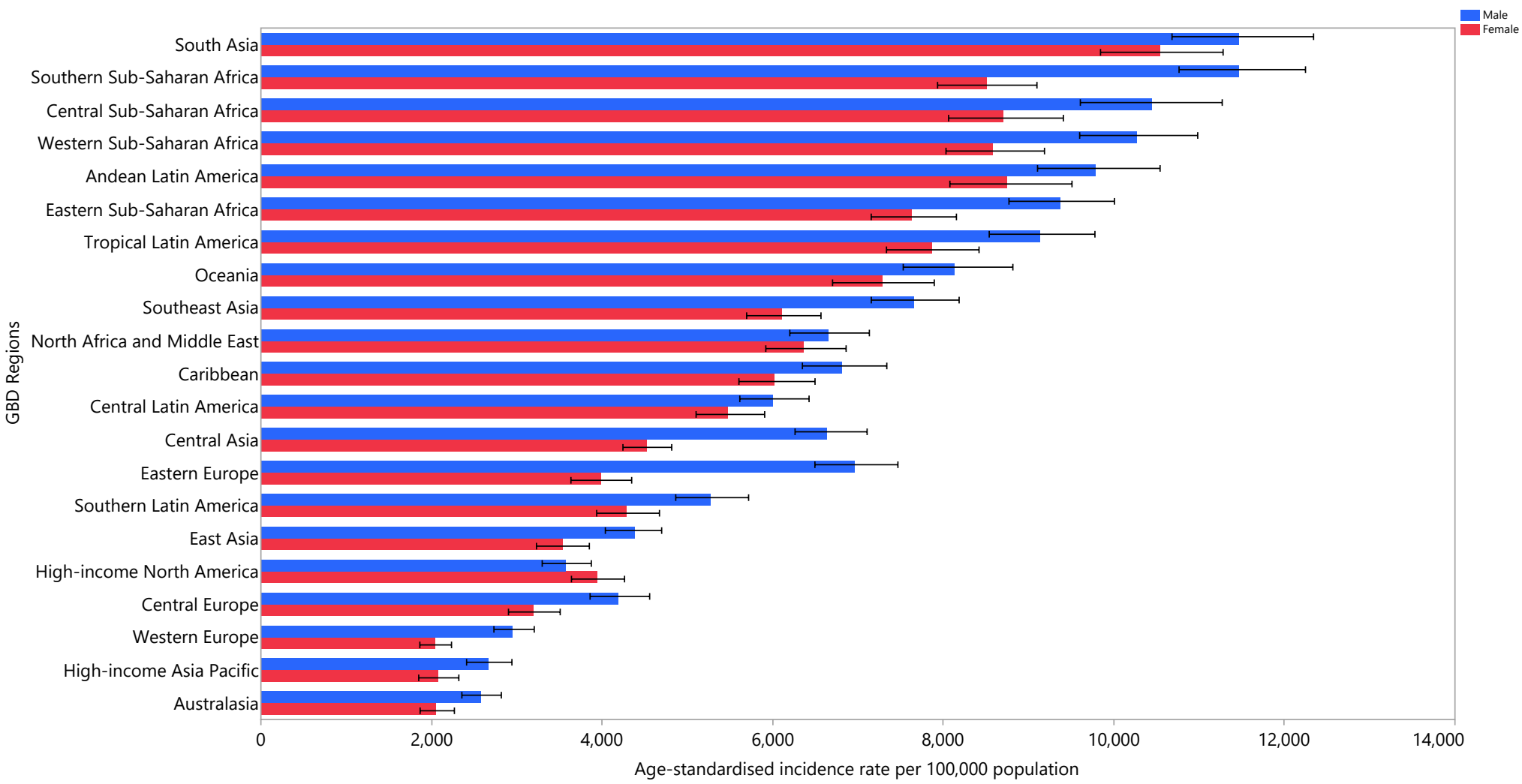

Supplement: Figure S1 — The age-standardised incidence of lower respiratory infections in 2019 for the 21 Global Burden of Disease regions by sex (generated from data available from http://ghdx.healthdata.org/gbd-results-tool). [file Image_1.PDF]

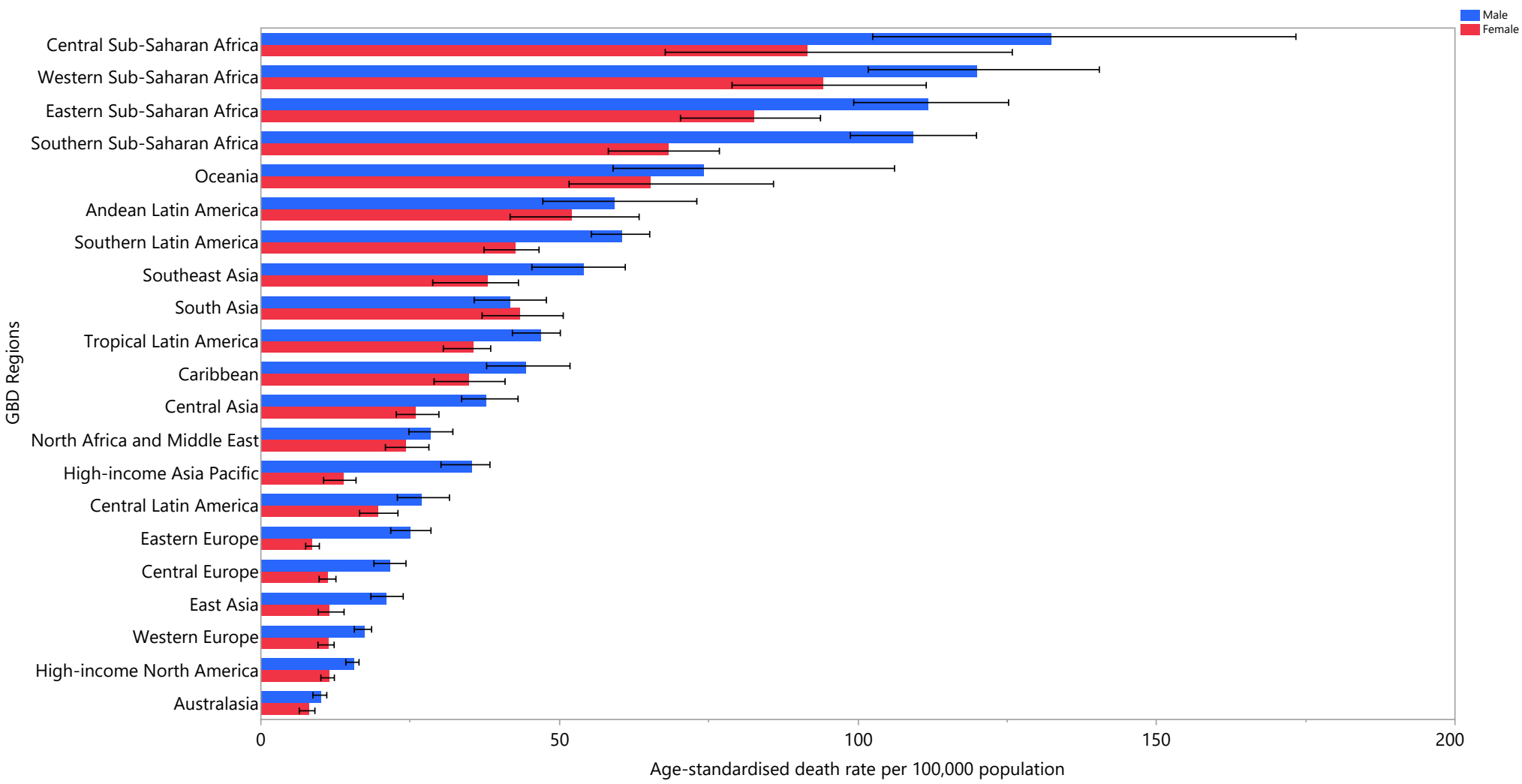

Supplement: Figure S2 — The age-standardised death rates of lower respiratory infections in 2019 for the 21 Global Burden of Disease regions by sex (generated from data available from http://ghdx.healthdata.org/gbd-results-tool). [file Image_2.PDF]

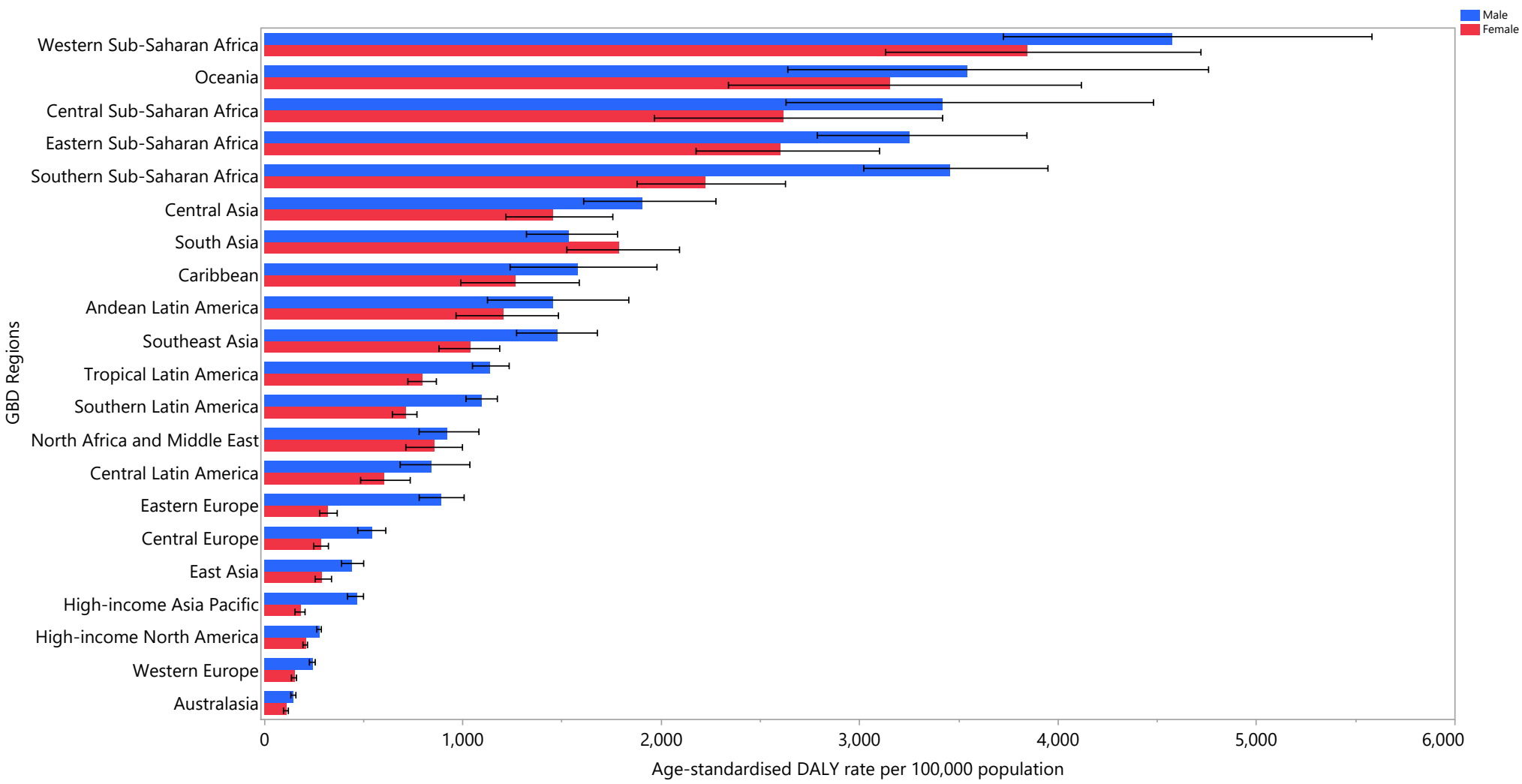

Supplement: Figure S3 — The age-standardised DALY rates of lower respiratory infections in 2019 for the 21 Global Burden of Disease regions by sex DALY=disability adjusted life years (generated from data available from http://ghdx.healthdata.org/gbd-results-tool). [file Image_3.PDF]

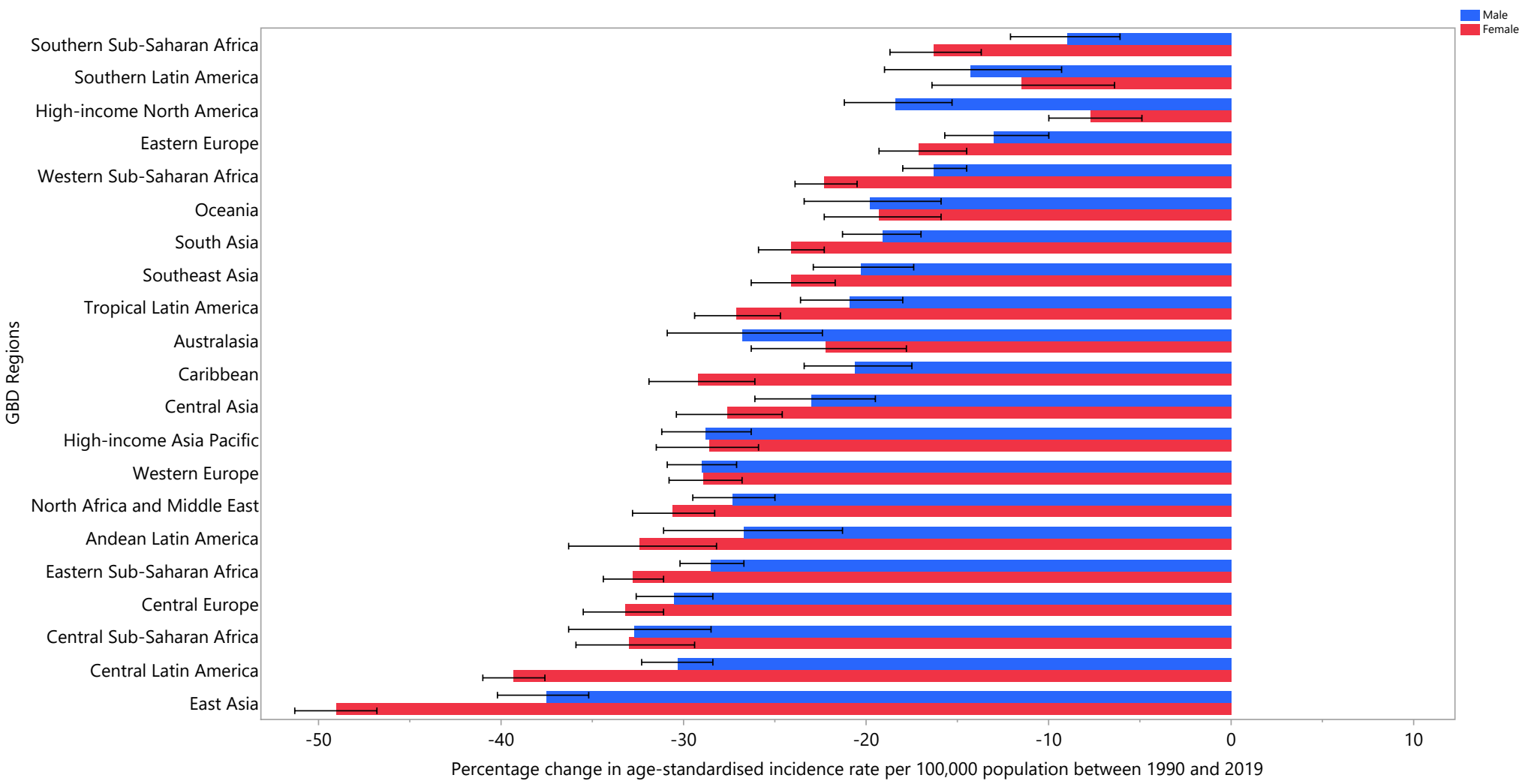

Supplement: Figure S4 — The percentage change in the age-standardised incidence rate of lower respiratory infections from 1990 to 2019 for the 21 Global Burden of Disease regions by sex (generated from data available from http://ghdx.healthdata.org/gbd-results-tool). [file Image_4.PDF]

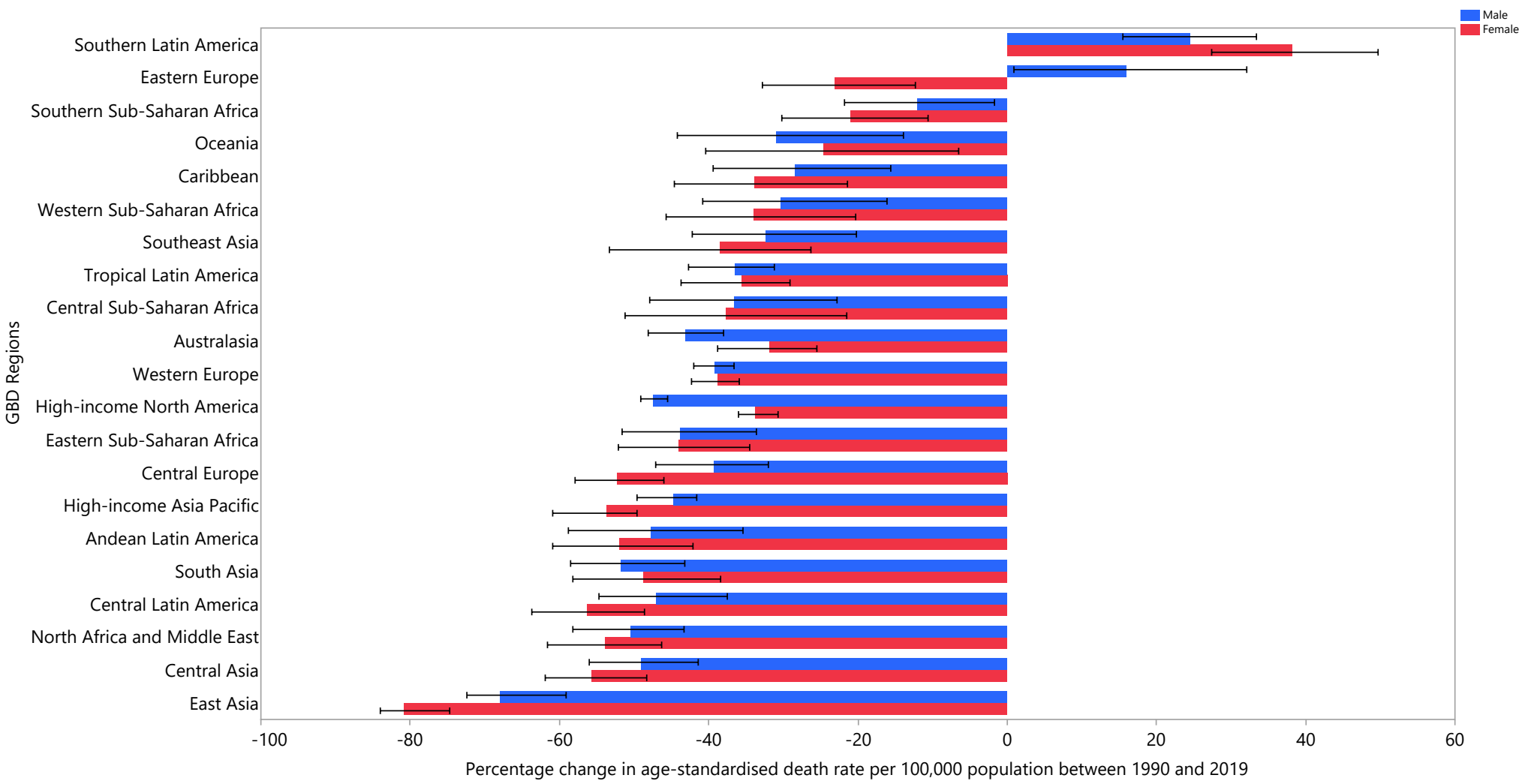

Supplement: Figure S5 — The percentage change in the age-standardised death rates of lower respiratory infections from 1990 to 2019 for the 21 Global Burden of Disease regions by sex (generated from data available from http://ghdx.healthdata.org/gbd-results-tool). [file Image_5.PDF]

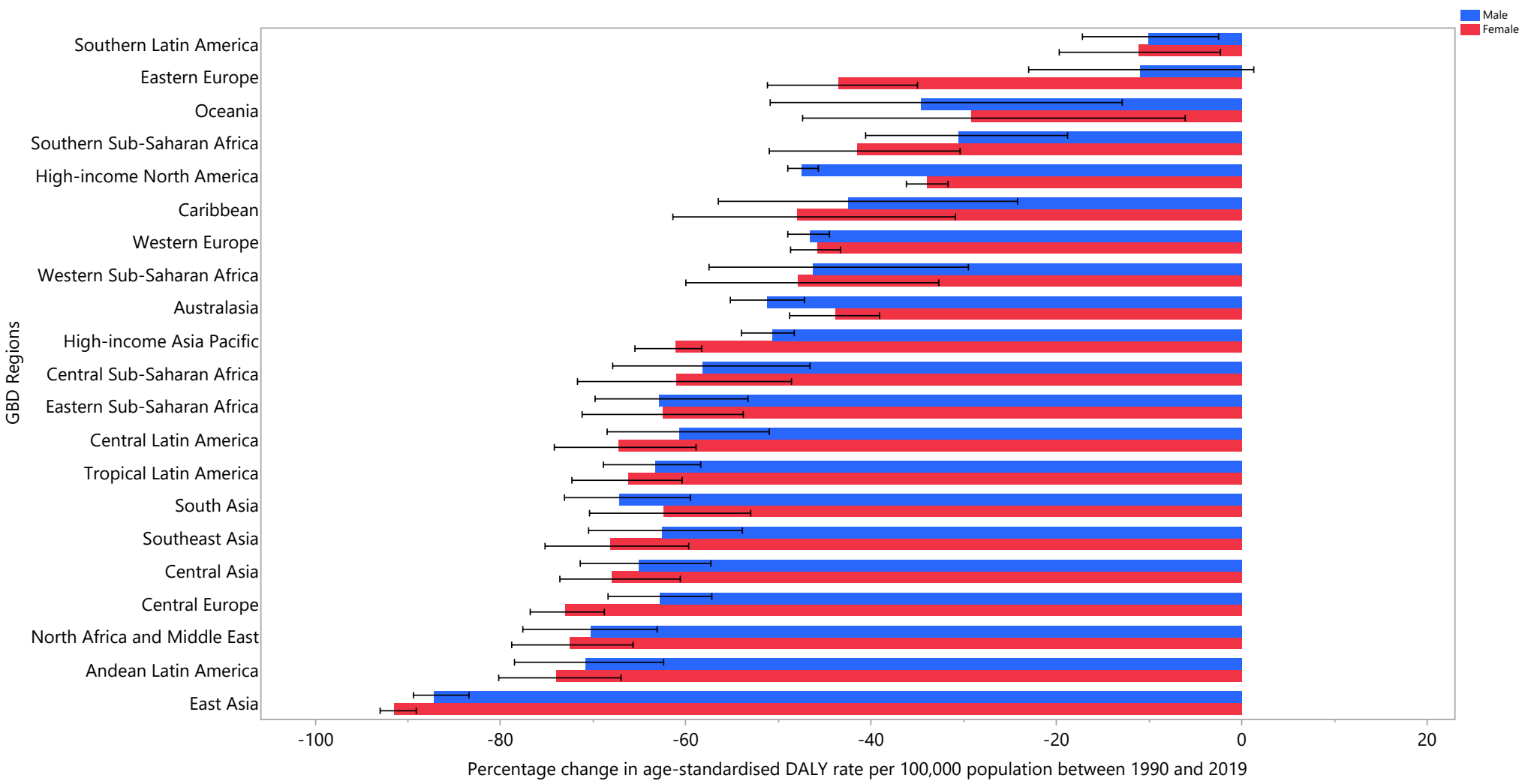

Supplement: Figure S6 — The percentage change in the age-standardised DALY rates of lower respiratory infections from 1990 to 2019 for the 21 Global Burden of Disease regions by sex. DALY=disability adjusted life years (generated from data available from http://ghdx.healthdata.org/gbd-results-tool). [file Image_6.PDF]

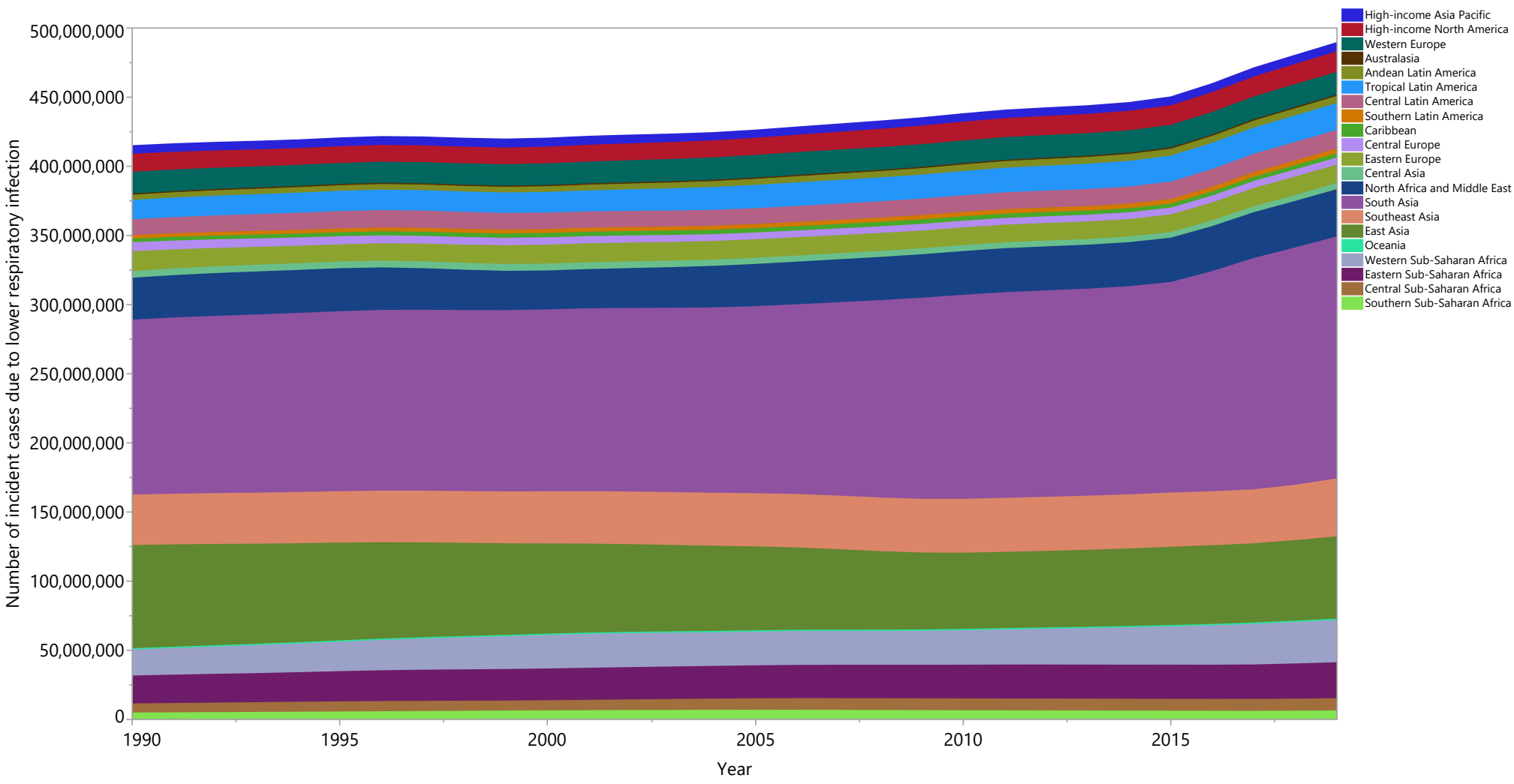

Supplement: Figure S7 — Number of incident cases of lower respiratory infections from 1990 to 2019 for the 21 Global Burden of Disease regions (generated from data available from http://ghdx.healthdata.org/gbd-results-tool). [file Image_7.PDF]

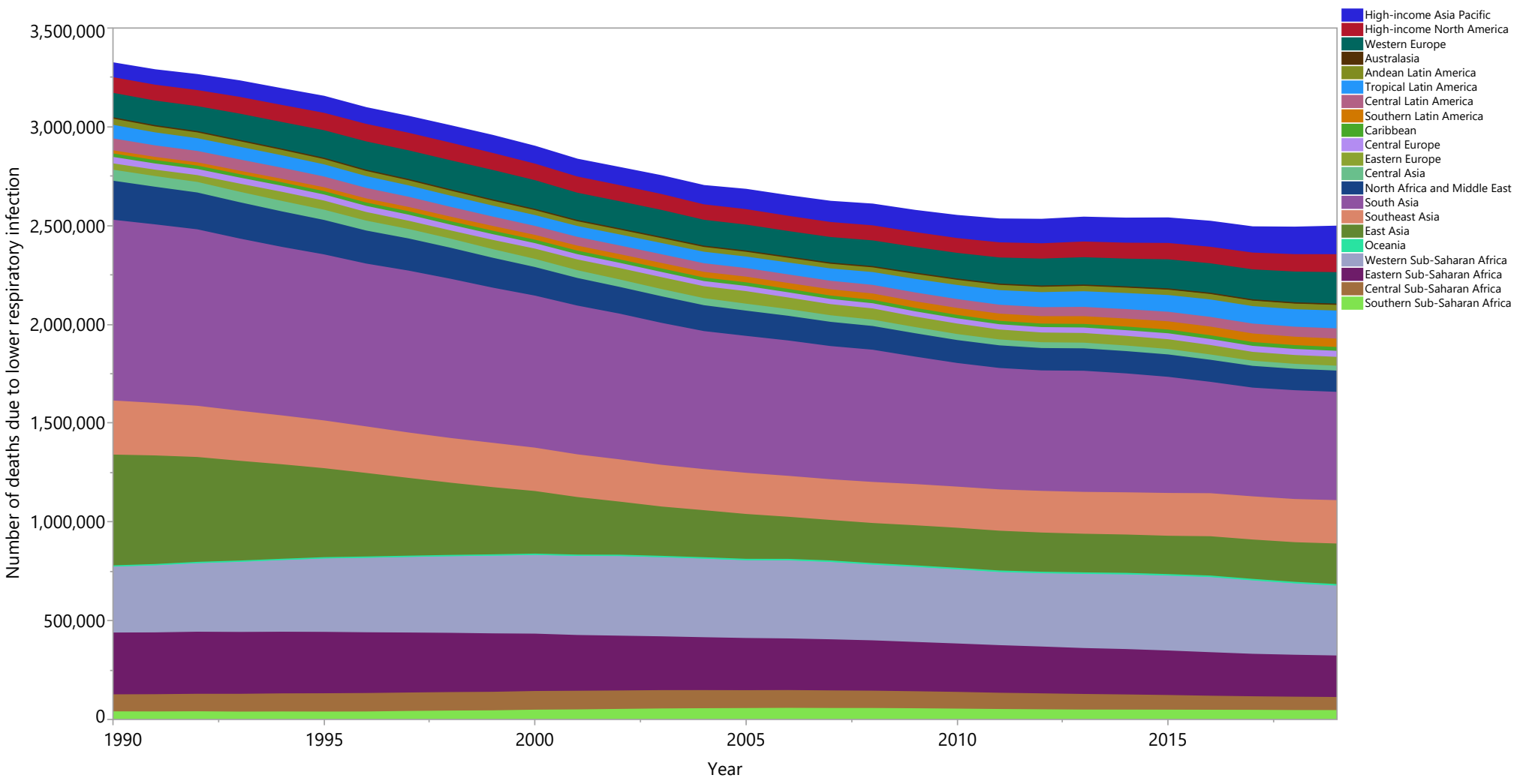

Supplement: Figure S8 — Number of deaths due to lower respiratory infections from 1990 to 2019 for the 21 Global Burden of Disease regions (generated from data available from http://ghdx.healthdata.org/gbd-results-tool). [file Image_8.PDF]

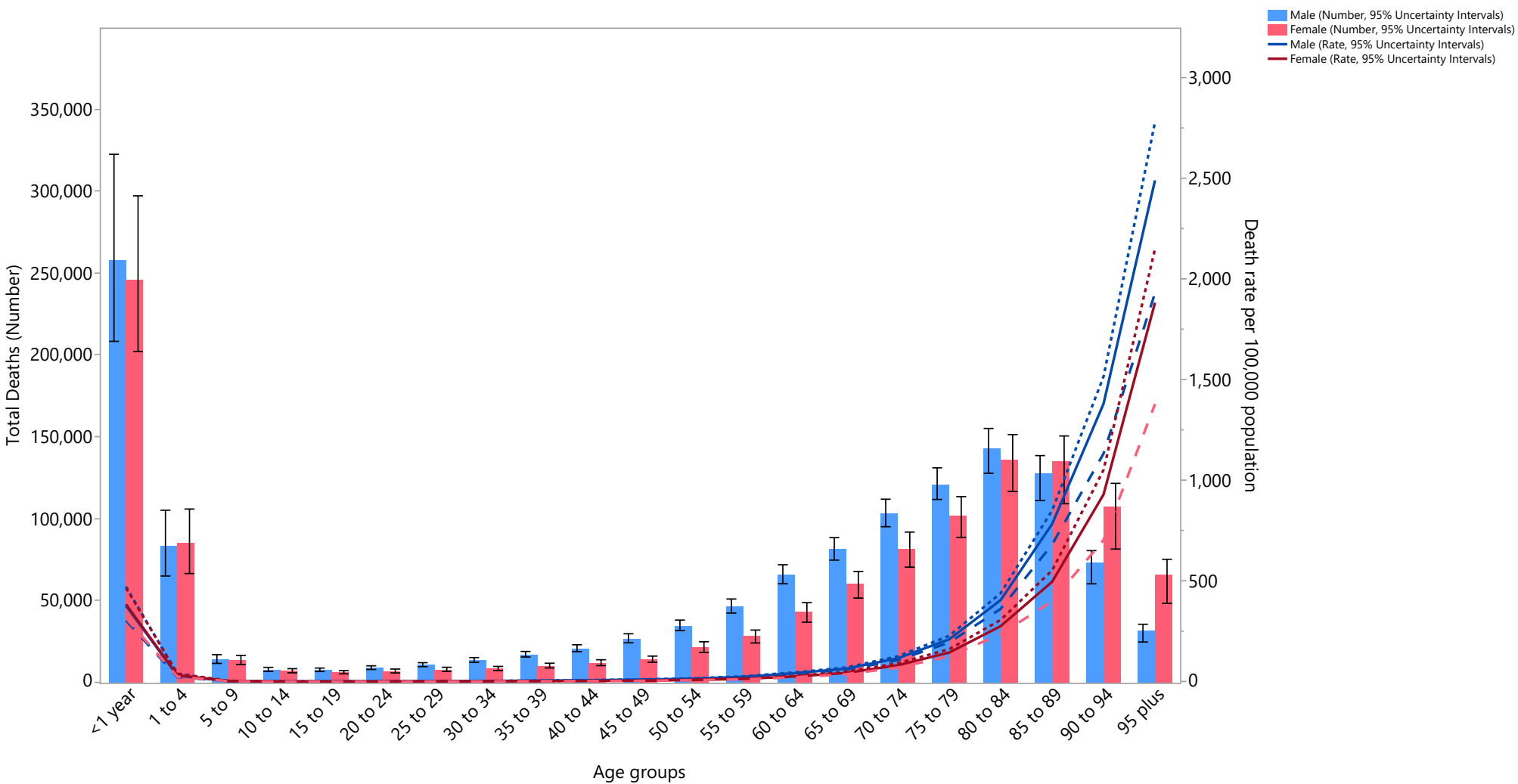

Supplement: Figure S10 — Global number of deaths and the death rate from lower respiratory infections (per 100,000 population) in 2019, by age and sex; Dotted and dashed lines indicate 95% upper and lower uncertainty intervals, respectively (generated from data available from http://ghdx.healthdata.org/gbd-results-tool). [file Image_10.PDF]

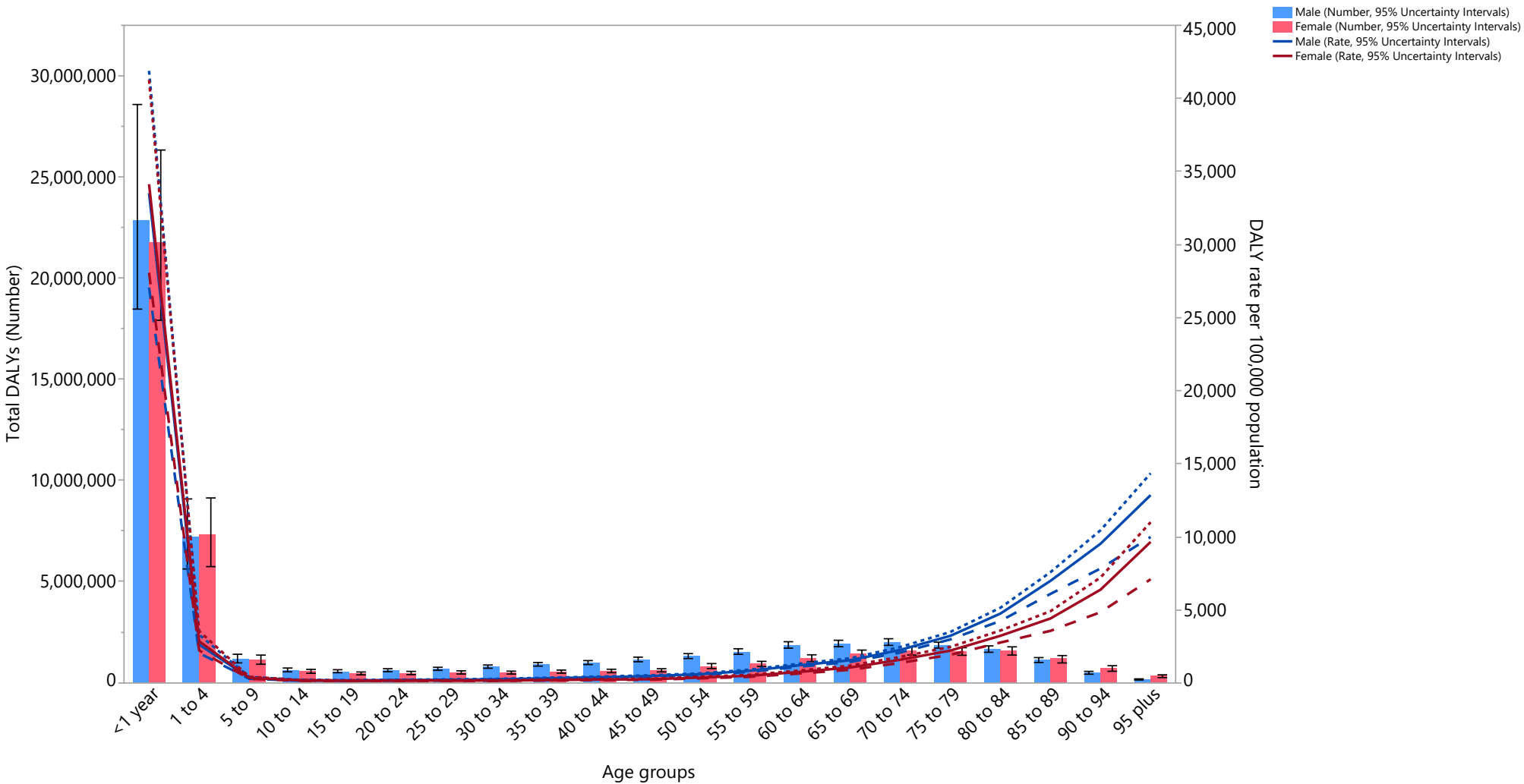

Supplement: Figure S11 — Global number of DALYs and the DALY rate from lower respiratory infections (per 100,000 population) in 2019, by age and sex; Dotted and dashed lines indicate 95% upper and lower uncertainty intervals, respectively. DALY=disability adjusted life years (generated from data available from http://ghdx.healthdata.org/gbd-results-tool). [file Image_11.PDF]

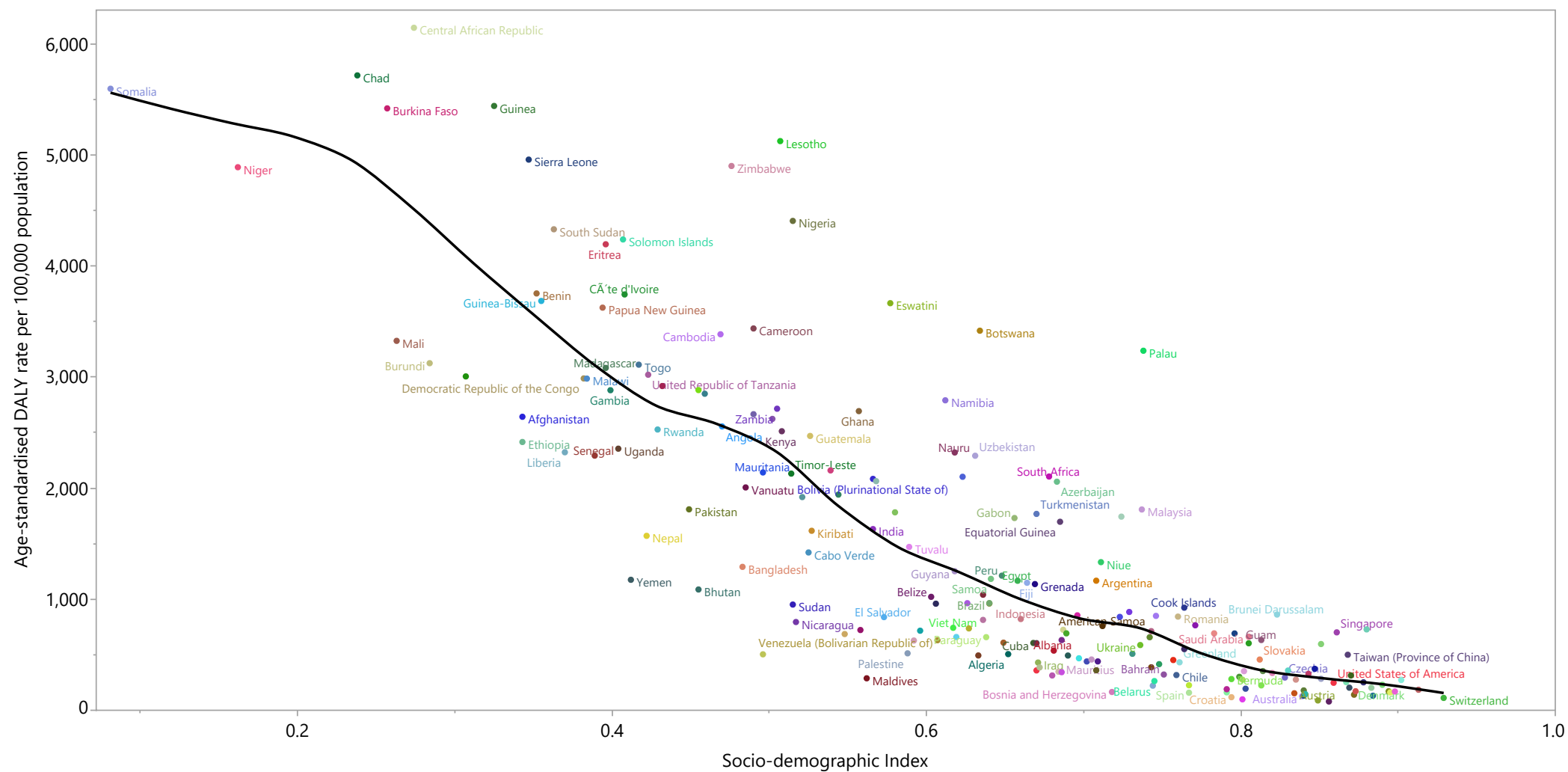

Supplement: Figure S12 — Age-standardised DALY rates of lower respiratory infections for 204 countries and territories in 2019, by SDI; Expected values based on the Socio-demographic Index and disease rates in all locations are shown as the black line. Each point shows the observed age-standardised DALY rate for each country in 2019. DALY=disability adjusted life years. SDI, Socio-demographic Index (generated from data available from http://ghdx.healthdata.org/gbd-results-tool). [file Image_12.PDF]

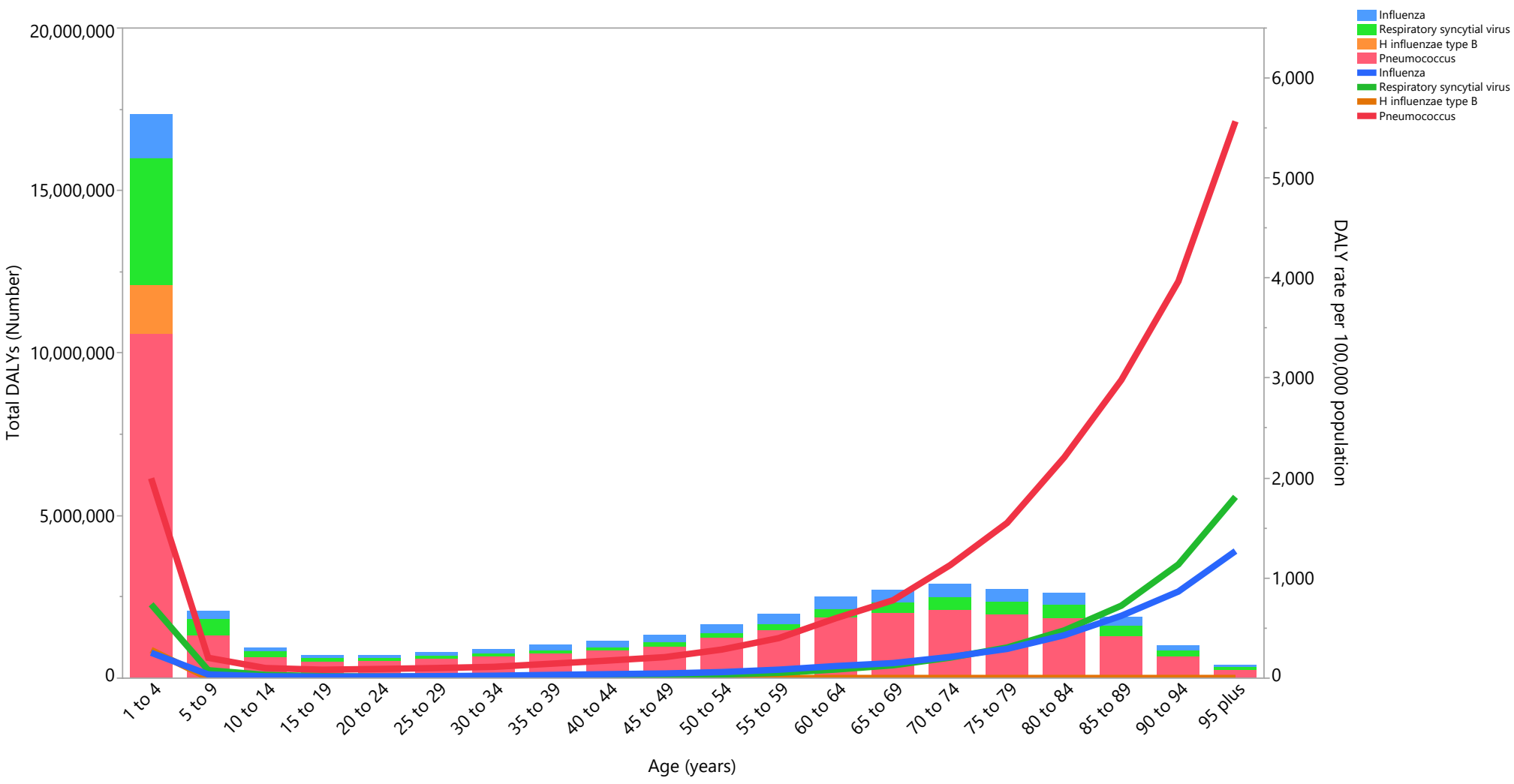

Supplement: Figure S13 — Global number of DALY and the DALY rate due to lower respiratory infections (per 100,000 population) in 2019, by aetiology and age. DALY=disability adjusted life years (generated from data available from http://ghdx.healthdata.org/gbd-results-tool). [file Image_13.PDF]

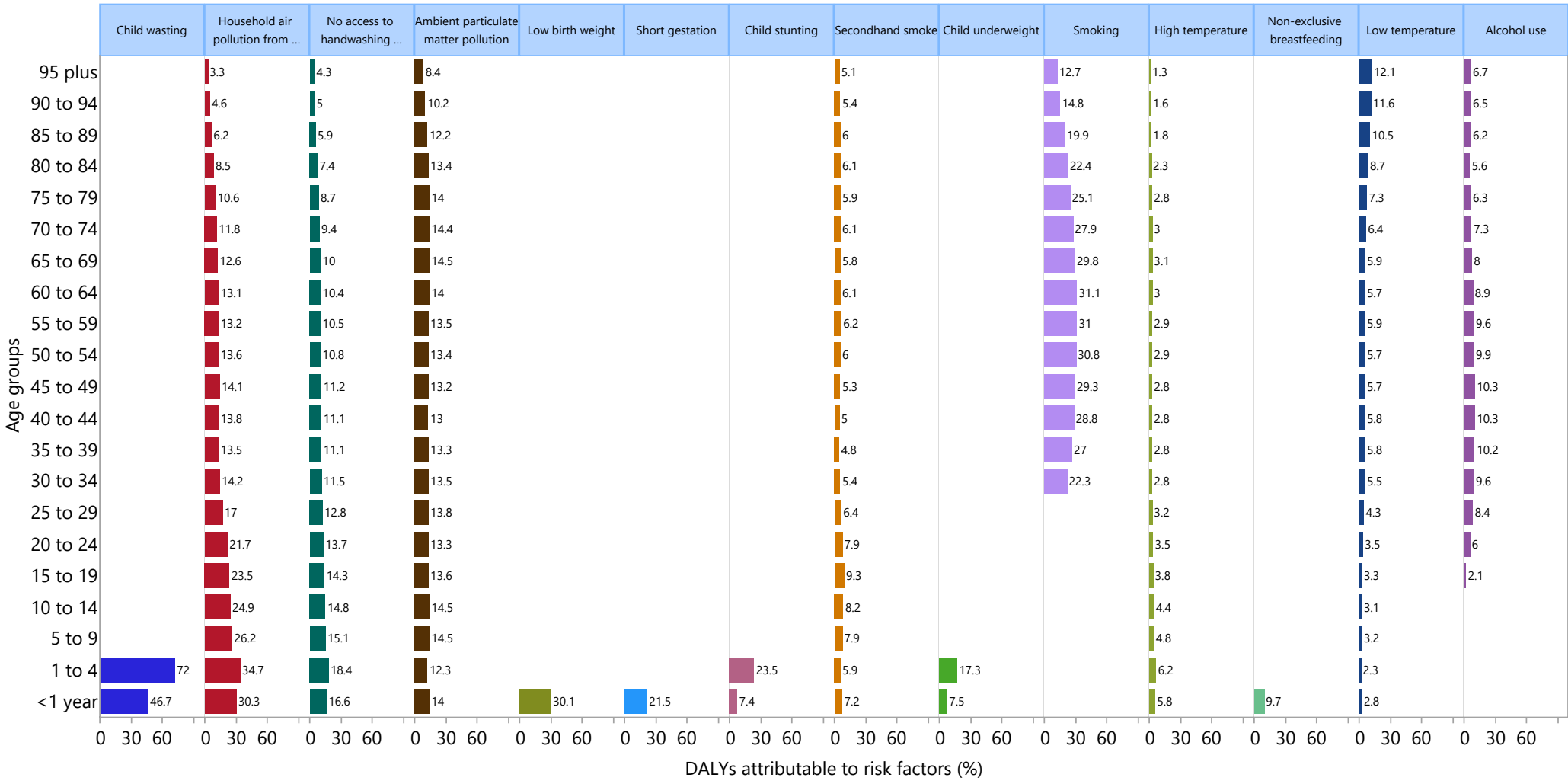

Supplement: Figure S14 — Percentage of DALYs due to lower respiratory infections attributable to risk factors for the 21 GBD regions in 2019, for males. DALY=disability adjusted life years (generated from data available from http://ghdx.healthdata.org/gbd-results-tool). [file Image_14.PDF]

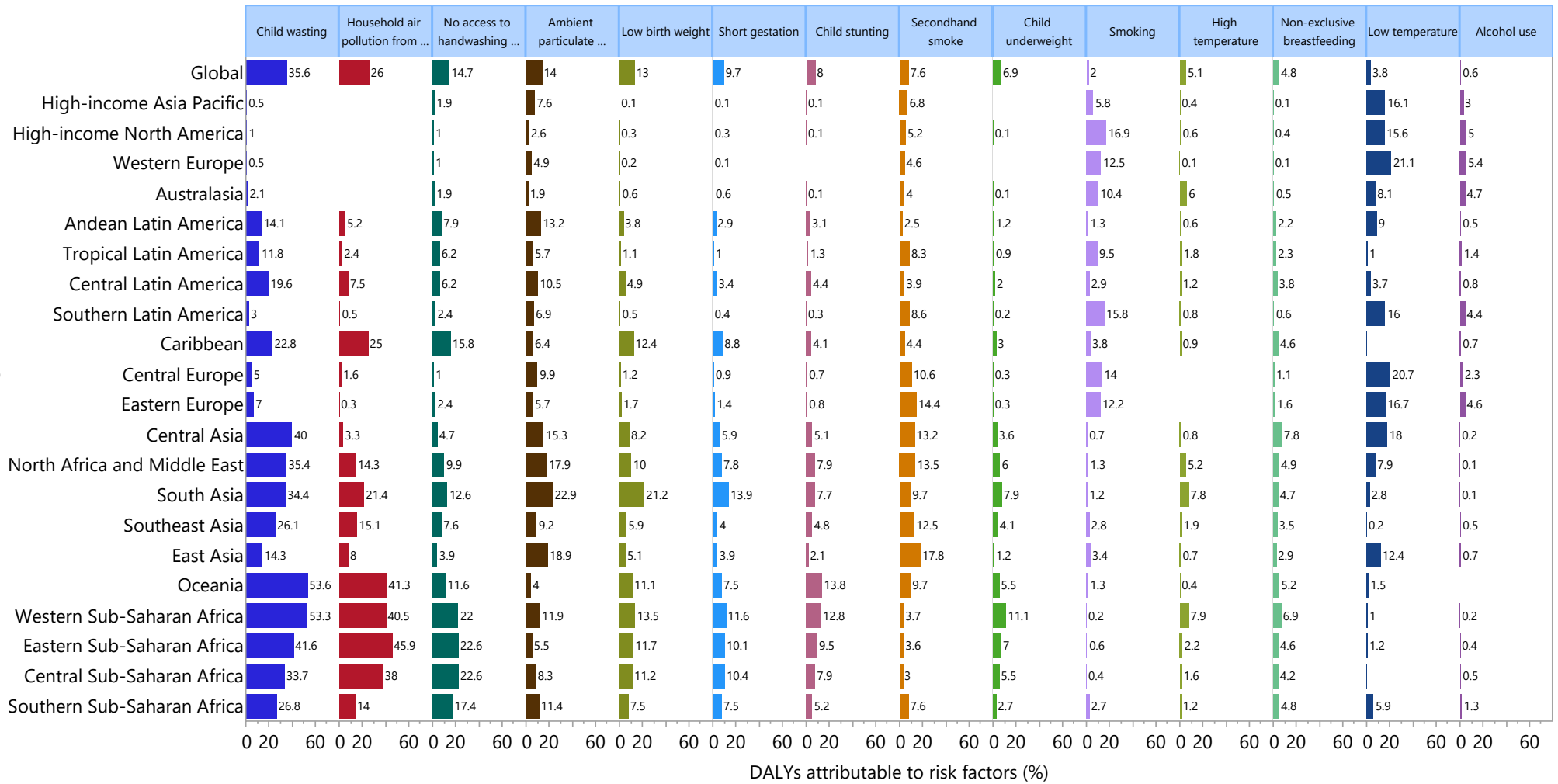

Supplement: Figure S15 — Percentage of DALYs due to lower respiratory infections attributable to risk factors for the 21 GBD regions in 2019, for females. DALY=disability adjusted life years (generated from data available from http://ghdx.healthdata.org/gbd-results-tool). [file Image_15.PDF]

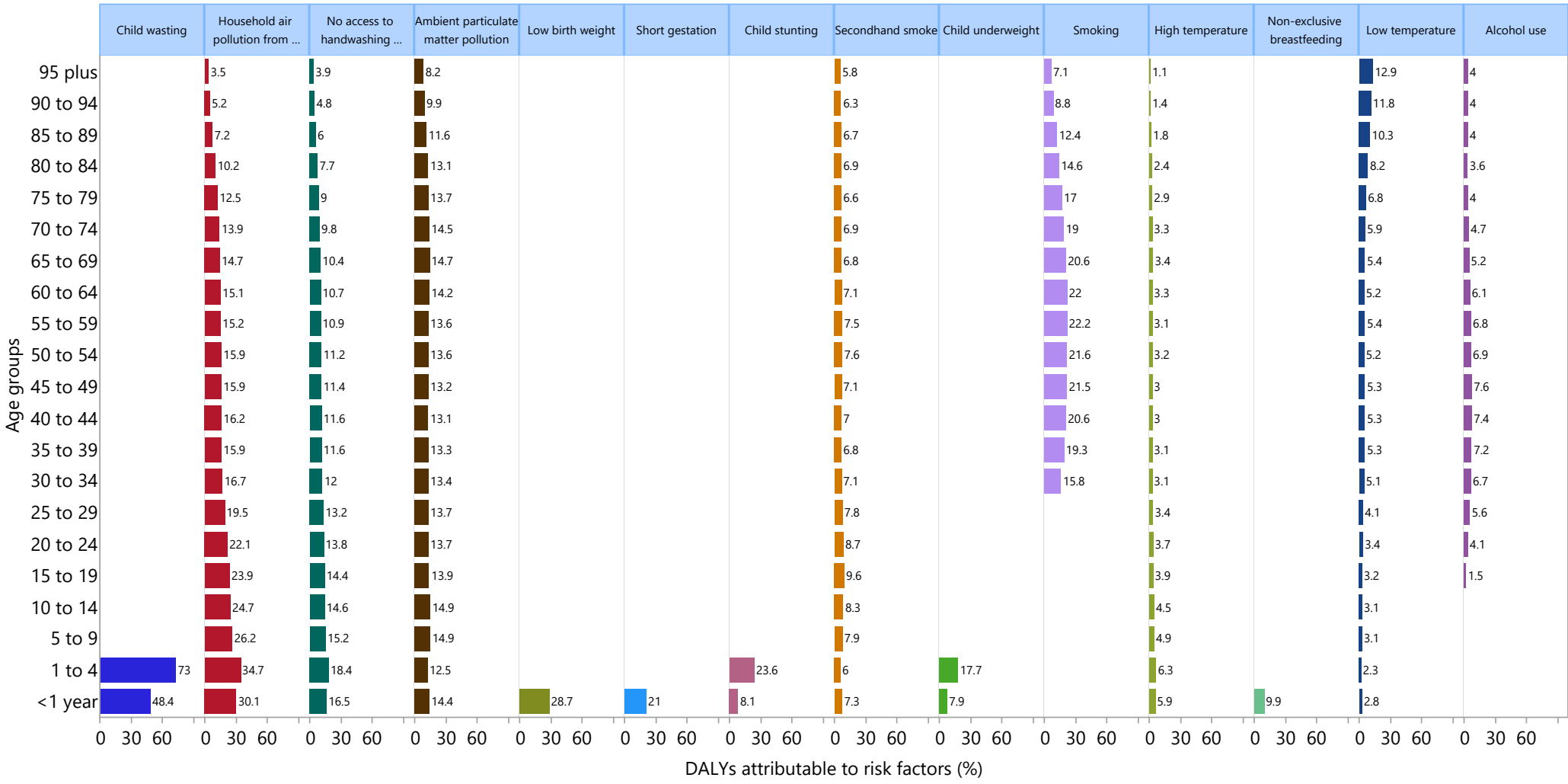

Supplement: Figure S16 — Percentage of DALYs due to lower respiratory infections attributable to each risk factor in 2019, by age. DALY=disability adjusted life years (generated from data available from http://ghdx.healthdata.org/gbd-results-tool). [file Presentation_1.PDF]

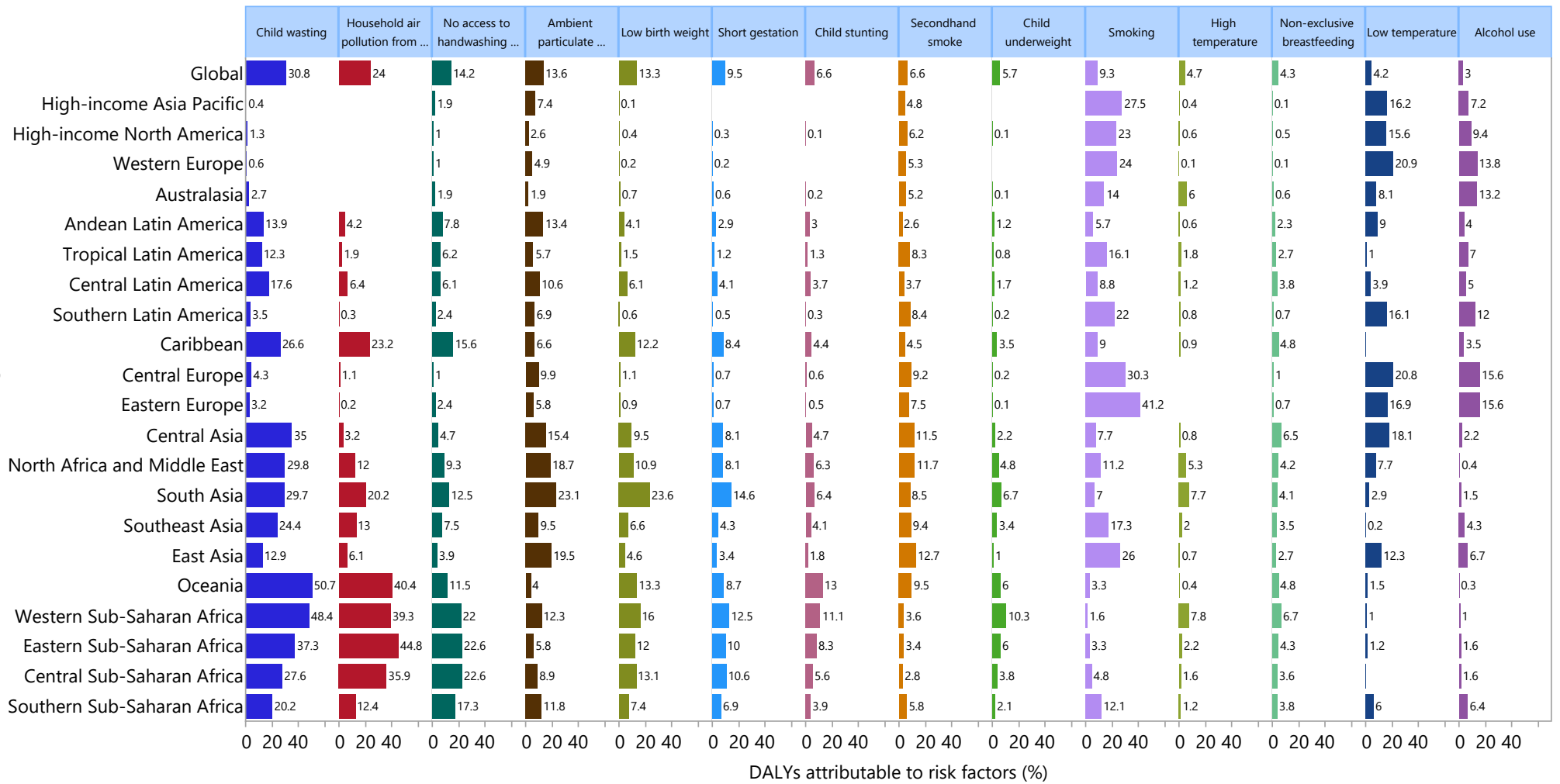

Supplement: Figure S17 — Percentage of DALYs due to lower respiratory infections attributable to each risk factor by age, for males, in 2019. DALY=disability adjusted life years (generated from data available from http://ghdx.healthdata.org/gbd-results-tool). [file Presentation_2.PDF]

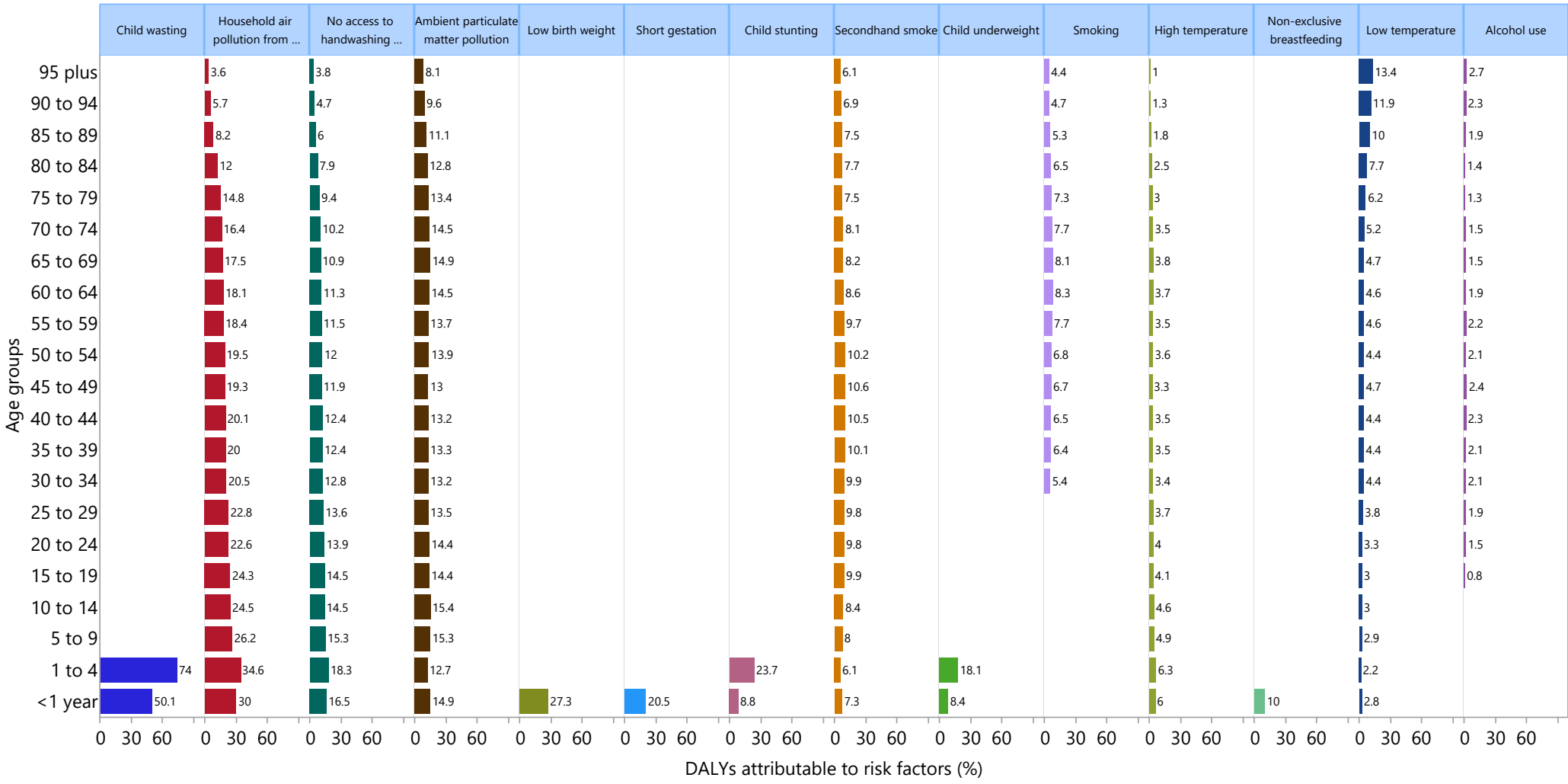

Supplement: Figure S18 — Percentage of DALYs due to lower respiratory infection attributable to each risk factor by age, for females, in 2019. DALY, disability adjusted life years (generated from data available from http://ghdx.healthdata.org/gbd-results-tool). [file Presentation_3.PDF]
