# Supplementary material for: Global burden of lower respiratory infections during the last three decades
Source: Front Public Health. 2023 Jan 9;10:1028525. doi: 10.3389/fpubh.2022.1028525 (PMC9869262; doi:10.3389/fpubh.2022.1028525)
Supplement: Figure S9 — Age-standardised DALY rate of lower respiratory infections (per 100,000 population) in 2019, by country. DALY=disability adjusted life years (generated from data available from http://ghdx.healthdata.org/gbd-results-tool). [file Image_9.PDF]

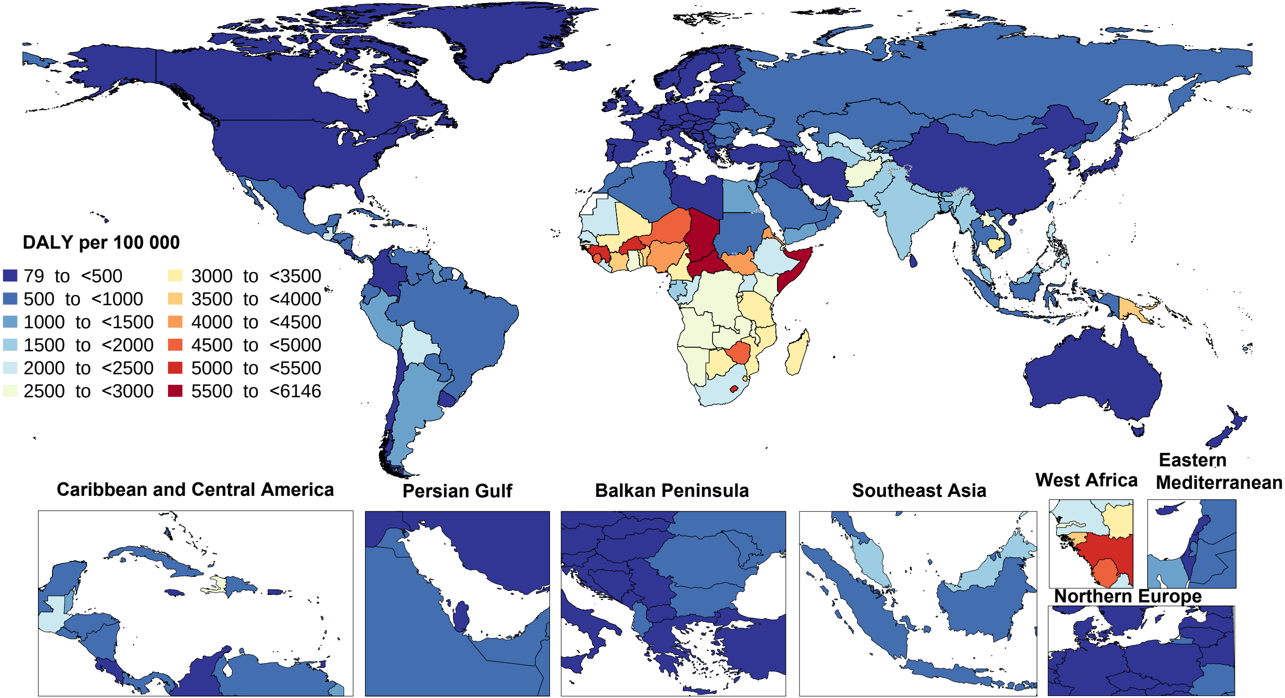
DALY per 100 000
79 to <500
500 to <1000
1000 to <1500
1500 to <2000
2000 to <2500
2500 to <3000
3000 to <3500
3500 to <4000
4000 to <4500
4500 to <5000
5000 to <5500
5500 to <6146
Caribbean and Central America
Persian Gulf
Balkan Peninsula
Southeast Asia
West Africa
Eastern Mediterranean
Northern Europe
