# Supplementary material for: Global burden of lower respiratory infections during the last three decades
Source: Front Public Health. 2023 Jan 9;10:1028525. doi: 10.3389/fpubh.2022.1028525 (PMC9869262; doi:10.3389/fpubh.2022.1028525)
Supplement: Table S1 — Sequelae for lower respiratory infections and their corresponding disability weights in the GBD 2019 study. [file Table_1.DOCX]

| **Table S1: Sequelae for lower respiratory infections and their corresponding disability weights in the GBD 2019 Study.** | | |
| --- | --- | --- |
| **Severity level** | **Lay description** | **DW**  **(95% CI)** |
| Moderate | Has a fever and aches and feels weak which causes some difficulty with daily activities. | 0.051 (0.032 to 0.074) |
| Severe | Has a high fever and pain and feels very weak, which causes great difficulty with daily activities. | 0.133 (0.088 to 0.19) |
