# Supplementary material for: Global burden of lower respiratory infections during the last three decades
Source: Front Public Health. 2023 Jan 9;10:1028525. doi: 10.3389/fpubh.2022.1028525 (PMC9869262; doi:10.3389/fpubh.2022.1028525)
Supplement: Table S2 — Incident cases of lower respiratory infections in 1990 and 2019 and the percentage change in the age-standardised rates (ASRs) per 100,000, by location (generated from data available from http://ghdx.healthdata.org/gbd-results-tool). [file Table_2.DOC]

| **Table S2: Incident cases of lower respiratory infections in 1990 and 2019 and the percentage change in the age-standardised rates (ASRs) per 100,000, by location**  **(Generated from data available from http://ghdx.healthdata.org/gbd-results-tool)** | | | | | |
| --- | --- | --- | --- | --- | --- |
|  | **1990** | | **2019** | | **Percentage change in ASRs per 100,000** |
|  | **No. (95% UI)** | **ASRs per 100,000 (95% UI)** | **No. (95% UI)** | **ASRs per 100,000 (95% UI)** |
| **Global** | **414342866 (383529625 , 449086938)** | **8276.2 (7726.7 , 8892)** | **488902504 (457572987 , 522635542)** | **6295 (5887.4 , 6737.3)** | **-23.9 (-25.4 , -22.5)** |
| **High-income North America** | **13069503 (12193475 , 14074453)** | **4314.6 (3991.6 , 4663.2)** | **14868900 (13950171 , 15845711)** | **3757.7 (3471.9 , 4059.7)** | **-12.9 (-15.2 , -10.2)** |
| **Canada** | **1118737 (1027162 , 1214552)** | **4003.6 (3640.8 , 4371.3)** | **1245672 (1145614 , 1342485)** | **3018 (2736.6 , 3306.2)** | **-24.6 (-28.2 , -21)** |
| **Greenland** | **2398 (2198 , 2604)** | **5028.8 (4620.1 , 5436.9)** | **1994 (1821 , 2161)** | **3469.6 (3160.7 , 3762.9)** | **-31 (-34.7 , -27)** |
| **United States of America** | **11948068 (11149619 , 12836115)** | **4346.8 (4027.3 , 4701.9)** | **13620998 (12780019 , 14515465)** | **3838.4 (3548.5 , 4146.6)** | **-11.7 (-14.2 , -8.7)** |
| **Australasia** | **624804 (575047 , 682935)** | **3037.9 (2781 , 3330)** | **847915 (786727 , 917942)** | **2303.3 (2113.4 , 2524.6)** | **-24.2 (-27.3 , -20.8)** |
| **Australia** | **477681 (438658 , 523175)** | **2798.9 (2559.3 , 3087)** | **650507 (601379 , 709183)** | **2100.5 (1913.4 , 2315.6)** | **-25 (-28.7 , -20.5)** |
| **New Zealand** | **147123 (134320 , 160783)** | **4199.4 (3826 , 4584.7)** | **197408 (182872 , 213165)** | **3395.2 (3115.5 , 3672.7)** | **-19.2 (-23.2 , -15)** |
| **High-income Asia Pacific** | **5210341 (4817371 , 5642319)** | **3247.8 (2967.9 , 3556.6)** | **5700982 (5328085 , 6083720)** | **2344.8 (2101.7 , 2597.7)** | **-27.8 (-30 , -25.4)** |
| **Brunei Darussalam** | **7273 (6451 , 8129)** | **3131.6 (2854.9 , 3413.8)** | **9673 (8783 , 10674)** | **2808 (2551.3 , 3068.6)** | **-10.3 (-14.3 , -6.1)** |
| **Japan** | **3996515 (3705694 , 4338037)** | **3218.7 (2923.4 , 3542.4)** | **4565314 (4273937 , 4878758)** | **2423.6 (2176.5 , 2678.4)** | **-24.7 (-27 , -22.1)** |
| **Singapore** | **113833 (104705 , 123134)** | **4500.3 (4127.7 , 4931.5)** | **155897 (145605 , 169002)** | **2895.1 (2629.5 , 3185.3)** | **-35.7 (-39.1 , -31.7)** |
| **Republic of Korea** | **1092720 (971546 , 1214388)** | **2817.9 (2533.1 , 3113.6)** | **970097 (887786 , 1056906)** | **2036.4 (1799.4 , 2292.8)** | **-27.7 (-32.2 , -23.2)** |
| **Western Europe** | **15700629 (14685297 , 16837555)** | **3426 (3163.8 , 3717)** | **16395485 (15264664 , 17666073)** | **2453.1 (2259.8 , 2678.5)** | **-28.4 (-30 , -26.8)** |
| **Andorra** | **1371 (1248 , 1494)** | **2966.2 (2710.9 , 3260.5)** | **2311 (2115 , 2521)** | **2247.2 (2045.7 , 2469.2)** | **-24.2 (-28.1 , -20.3)** |
| **Austria** | **301177 (279345 , 326275)** | **3402.7 (3130.6 , 3700)** | **286647 (261238 , 311886)** | **2467.7 (2219.8 , 2711)** | **-27.5 (-32 , -23.6)** |
| **Belgium** | **451530 (419120 , 487340)** | **3741 (3433.5 , 4077.5)** | **593850 (547785 , 649258)** | **3305.5 (3046 , 3625.8)** | **-11.6 (-15.9 , -6)** |
| **Cyprus** | **22162 (20303 , 24211)** | **3069.5 (2818.7 , 3341.8)** | **28833 (26569 , 31480)** | **1983.2 (1803.8 , 2183.7)** | **-35.4 (-38.7 , -31.6)** |
| **Denmark** | **222306 (204999 , 245728)** | **3529.9 (3239.2 , 3898.9)** | **222732 (202693 , 243227)** | **2649.5 (2412.3 , 2918.7)** | **-24.9 (-29.1 , -20.7)** |
| **Finland** | **267682 (245887 , 287643)** | **4474.6 (4109.4 , 4847)** | **174213 (159359 , 189934)** | **2346.8 (2107.3 , 2611.6)** | **-47.6 (-50.8 , -43.8)** |
| **France** | **2287440 (2117208 , 2473391)** | **3335.2 (3059.7 , 3647.6)** | **2494553 (2295599 , 2719934)** | **2435.7 (2214.7 , 2678.2)** | **-27 (-31.2 , -23)** |
| **Germany** | **3029299 (2796090 , 3275681)** | **3175 (2906 , 3481.5)** | **3057908 (2816554 , 3311570)** | **2393.7 (2182.4 , 2635.8)** | **-24.6 (-28.5 , -20.5)** |
| **Greece** | **410315 (379863 , 454082)** | **3515.4 (3217 , 3909.4)** | **451312 (413537 , 495626)** | **2715.5 (2493.6 , 2992.2)** | **-22.8 (-26.8 , -18.1)** |
| **Iceland** | **10938 (10100 , 11952)** | **4053.3 (3731.6 , 4452.9)** | **11911 (11061 , 12852)** | **2656.7 (2447.6 , 2898.6)** | **-34.5 (-37.8 , -30.9)** |
| **Ireland** | **167528 (155729 , 180741)** | **4401.9 (4096.1 , 4751.1)** | **154011 (142351 , 166763)** | **2533.3 (2332.8 , 2780.5)** | **-42.5 (-45.9 , -39)** |
| **Israel** | **138890 (126985 , 152507)** | **2852.4 (2616.8 , 3122.6)** | **237757 (217320 , 261165)** | **2223.2 (2022.9 , 2441.8)** | **-22.1 (-26.6 , -17.4)** |
| **Italy** | **1804944 (1675344 , 1956862)** | **2896.9 (2640.7 , 3207)** | **2405859 (2243730 , 2594305)** | **2329.1 (2151 , 2532.1)** | **-19.6 (-22.4 , -16.9)** |
| **Luxembourg** | **13042 (12005 , 14252)** | **3009.1 (2743.7 , 3317.2)** | **17606 (16165 , 19126)** | **2273.8 (2079.5 , 2505.4)** | **-24.4 (-28.4 , -20.4)** |
| **Malta** | **16151 (14819 , 17735)** | **4242.1 (3889.3 , 4654.8)** | **19874 (18256 , 21728)** | **3002.8 (2750.7 , 3281.4)** | **-29.2 (-33.3 , -25.2)** |
| **Monaco** | **1598 (1455 , 1762)** | **3233.6 (2970.9 , 3526.4)** | **1903 (1733 , 2088)** | **2763 (2528.5 , 3011)** | **-14.6 (-18.6 , -9.9)** |
| **Netherlands** | **468751 (427498 , 509211)** | **2774.8 (2524.4 , 3042.1)** | **526835 (482029 , 578898)** | **2090.3 (1901.9 , 2308.5)** | **-24.7 (-29.1 , -20.4)** |
| **Norway** | **289255 (268223 , 315863)** | **4907.1 (4544.7 , 5329.9)** | **215365 (199532 , 230697)** | **2945.2 (2695.5 , 3214.4)** | **-40 (-42.5 , -37.7)** |
| **Portugal** | **558362 (512750 , 600430)** | **4930.6 (4550.5 , 5328.2)** | **580939 (537604 , 632828)** | **3289.4 (3027.9 , 3587.8)** | **-33.3 (-36.9 , -29.5)** |
| **San Marino** | **757 (694 , 827)** | **2888.9 (2644.9 , 3143.5)** | **1065 (969 , 1163)** | **2293.6 (2082.1 , 2506.5)** | **-20.6 (-24.6 , -16.9)** |
| **Spain** | **1255977 (1163165 , 1360706)** | **2899.6 (2662.8 , 3150.2)** | **1495224 (1379802 , 1612441)** | **2148.5 (1964.2 , 2348.5)** | **-25.9 (-30.1 , -21.8)** |
| **Sweden** | **477610 (444766 , 515205)** | **4065.6 (3776.6 , 4418.2)** | **404640 (373812 , 440104)** | **2902.9 (2629.4 , 3208.2)** | **-28.6 (-32 , -25.4)** |
| **Switzerland** | **240164 (221634 , 261534)** | **2857.7 (2607.8 , 3136.8)** | **223306 (204917 , 241835)** | **1775.9 (1607 , 1952)** | **-37.9 (-41.9 , -34.3)** |
| **United Kingdom** | **3250343 (3042422 , 3505528)** | **4332.1 (4033.4 , 4702.6)** | **2772539 (2578804 , 2974485)** | **2714.1 (2526.3 , 2942.2)** | **-37.3 (-38.6 , -36.1)** |
| **Southern Latin America** | **2616693 (2438656 , 2828668)** | **5453.7 (5099.1 , 5883.7)** | **3432983 (3186597 , 3703188)** | **4743.1 (4394.3 , 5122)** | **-13 (-16.7 , -9.2)** |
| **Argentina** | **1528153 (1416256 , 1670584)** | **4669.3 (4343.6 , 5089.7)** | **2532306 (2343798 , 2748796)** | **5205.3 (4812.3 , 5631.7)** | **11.5 (5.4 , 18.1)** |
| **Chile** | **914573 (852800 , 987194)** | **7844.1 (7323.1 , 8448.5)** | **739963 (681473 , 804934)** | **3902.7 (3558.6 , 4274.4)** | **-50.2 (-53.4 , -47)** |
| **Uruguay** | **173861 (160966 , 189618)** | **5256.2 (4843.6 , 5722.7)** | **160540 (149254 , 172475)** | **4028.4 (3707.4 , 4381.5)** | **-23.4 (-26.8 , -19.6)** |
| **Eastern Europe** | **14335512 (13332292 , 15432084)** | **6100.3 (5635.9 , 6595.7)** | **13294778 (12428403 , 14225691)** | **5232.6 (4834.9 , 5662.9)** | **-14.2 (-16.3 , -11.8)** |
| **Belarus** | **657896 (602864 , 720511)** | **6078.5 (5542.3 , 6667)** | **499437 (462464 , 540922)** | **4438.6 (4057 , 4854.2)** | **-27 (-31 , -22.7)** |
| **Estonia** | **123940 (113235 , 135324)** | **7324.6 (6674 , 8038)** | **86586 (79602 , 94948)** | **5011.3 (4555.2 , 5543.4)** | **-31.6 (-35.6 , -27.1)** |
| **Latvia** | **229134 (211661 , 250505)** | **7835.9 (7200 , 8566.7)** | **148267 (135349 , 161696)** | **5727.8 (5209.2 , 6297.3)** | **-26.9 (-31.4 , -22.6)** |
| **Lithuania** | **274311 (250464 , 300642)** | **7229.9 (6526.1 , 7968.8)** | **236910 (217726 , 258378)** | **6215.1 (5679.5 , 6825.3)** | **-14 (-18.7 , -9.7)** |
| **Republic of Moldova** | **334752 (310670 , 362296)** | **7683.3 (7120.7 , 8315.2)** | **245355 (225414 , 267066)** | **5560.5 (5109.8 , 6045.6)** | **-27.6 (-32.3 , -23)** |
| **Russian Federation** | **9201623 (8528671 , 9888465)** | **5968.1 (5514.9 , 6444.6)** | **9100557 (8488848 , 9738991)** | **5144.8 (4755.8 , 5541.4)** | **-13.8 (-15.7 , -11.7)** |
| **Ukraine** | **3513856 (3245069 , 3840963)** | **6116.2 (5619.2 , 6679.4)** | **2977667 (2729425 , 3231105)** | **5596 (5133 , 6070.6)** | **-8.5 (-13.9 , -2.1)** |
| **Central Europe** | **6462475 (6020574 , 6979778)** | **5314.2 (4911.8 , 5784.2)** | **5132184 (4799948 , 5523102)** | **3624.9 (3328.6 , 3964.6)** | **-31.8 (-33.7 , -30.1)** |
| **Albania** | **216367 (194351 , 239747)** | **7217 (6603.6 , 7923.4)** | **109014 (100091 , 118513)** | **3708 (3379.8 , 4050.6)** | **-48.6 (-51.7 , -45.3)** |
| **Bosnia and Herzegovina** | **189143 (171081 , 209102)** | **4796.8 (4327.3 , 5351.1)** | **125089 (114101 , 137556)** | **3531.3 (3167.1 , 3970.4)** | **-26.4 (-30.2 , -22.6)** |
| **Bulgaria** | **628422 (580761 , 690676)** | **7041.8 (6481 , 7748.7)** | **332226 (305608 , 362878)** | **3801.2 (3472.2 , 4192.3)** | **-46 (-49.9 , -42.1)** |
| **Croatia** | **232174 (214378 , 251366)** | **4717.3 (4299.4 , 5159.2)** | **154349 (141330 , 168001)** | **3083 (2761.5 , 3441.8)** | **-34.6 (-38 , -31.1)** |
| **Czechia** | **503834 (461452 , 552777)** | **4638 (4204 , 5144.8)** | **492252 (455962 , 538093)** | **3522.5 (3187.6 , 3872.2)** | **-24.1 (-27.4 , -19.7)** |
| **Hungary** | **423978 (387024 , 470182)** | **4062.6 (3679.8 , 4527.5)** | **362126 (333564 , 394920)** | **3265.8 (2904.5 , 3654.5)** | **-19.6 (-23.1 , -15.5)** |
| **Montenegro** | **24891 (22482 , 27526)** | **4240.5 (3813.7 , 4707.1)** | **24441 (22323 , 26785)** | **3743.8 (3361.3 , 4165.1)** | **-11.7 (-16.1 , -7.3)** |
| **North Macedonia** | **89910 (81664 , 99166)** | **4869.7 (4432 , 5370.2)** | **75620 (68834 , 82558)** | **3694.4 (3313.9 , 4106.3)** | **-24.1 (-28.8 , -18.9)** |
| **Poland** | **1527463 (1408725 , 1668400)** | **4003.2 (3662.7 , 4398.3)** | **1607141 (1497779 , 1727976)** | **3194.9 (2930.6 , 3476.8)** | **-20.2 (-22.1 , -18.1)** |
| **Romania** | **1795745 (1662590 , 1954217)** | **7884.5 (7263.5 , 8622.7)** | **1143415 (1053104 , 1239992)** | **4778.2 (4402.8 , 5192.7)** | **-39.4 (-43 , -35.5)** |
| **Serbia** | **347105 (317012 , 377831)** | **3835 (3467 , 4212.9)** | **319100 (292573 , 350924)** | **3127.3 (2809.2 , 3472.1)** | **-18.5 (-22.6 , -14)** |
| **Slovakia** | **375846 (345489 , 405669)** | **7055.5 (6487.8 , 7663.6)** | **288920 (264413 , 315201)** | **4471.1 (4055.5 , 4944.6)** | **-36.6 (-40.1 , -32.6)** |
| **Slovenia** | **107596 (98528 , 117911)** | **5471.5 (4944.9 , 6055.9)** | **98490 (90024 , 107575)** | **3690.9 (3311.1 , 4111.2)** | **-32.5 (-35.9 , -28.9)** |
| **Central Asia** | **4857818 (4534868 , 5212463)** | **7221.1 (6798.9 , 7698.7)** | **4453344 (4193895 , 4743352)** | **5436.3 (5133.8 , 5780.7)** | **-24.7 (-27.4 , -22.3)** |
| **Armenia** | **178484 (165101 , 194538)** | **5710.9 (5295.4 , 6198.6)** | **164526 (152792 , 178323)** | **4977.8 (4602.1 , 5404.2)** | **-12.8 (-17.2 , -7.9)** |
| **Azerbaijan** | **507837 (465121 , 552184)** | **7212.8 (6643 , 7807)** | **491050 (456917 , 526383)** | **5637.7 (5253.1 , 6068.5)** | **-21.8 (-26.7 , -16.5)** |
| **Georgia** | **372364 (346134 , 403690)** | **6986.5 (6488.7 , 7589.1)** | **292487 (269224 , 316828)** | **6375.9 (5891.1 , 6879.4)** | **-8.7 (-15.1 , -1.5)** |
| **Kazakhstan** | **926182 (855941 , 1014082)** | **6105.3 (5674 , 6667.8)** | **898479 (832055 , 973945)** | **5190.3 (4817.9 , 5629.3)** | **-15 (-19.7 , -9.9)** |
| **Kyrgyzstan** | **285554 (265168 , 308219)** | **6428.6 (6017 , 6851.7)** | **229743 (212571 , 248963)** | **3923.4 (3650.3 , 4217.5)** | **-39 (-43 , -34.8)** |
| **Mongolia** | **169627 (152554 , 188660)** | **8428.9 (7781.9 , 9180.1)** | **128007 (118568 , 138780)** | **4478.6 (4168.1 , 4834.6)** | **-46.9 (-49.9 , -43.7)** |
| **Tajikistan** | **503395 (461917 , 553012)** | **9557.6 (8875.1 , 10429.3)** | **470954 (432935 , 513256)** | **6400.2 (5932.1 , 6908.4)** | **-33 (-38.3 , -27.4)** |
| **Turkmenistan** | **271292 (249401 , 296124)** | **7477.8 (6977.4 , 8065.3)** | **206664 (192288 , 221285)** | **4424.8 (4127.2 , 4733)** | **-40.8 (-44.2 , -37.2)** |
| **Uzbekistan** | **1643082 (1511088 , 1777886)** | **7890.4 (7373.2 , 8454.5)** | **1571435 (1464424 , 1680772)** | **5681.3 (5311.6 , 6072)** | **-28 (-32.2 , -23)** |
| **Central Latin America** | **11368524 (10377980 , 12436260)** | **8825.8 (8259.9 , 9488.4)** | **13417558 (12537543 , 14385830)** | **5726.2 (5340.9 , 6136.2)** | **-35.1 (-36.7 , -33.6)** |
| **Colombia** | **2115284 (1928367 , 2317516)** | **8259 (7630.1 , 8942.2)** | **2674383 (2477312 , 2901510)** | **5408.3 (4989.5 , 5895.9)** | **-34.5 (-38.8 , -30.1)** |
| **Costa Rica** | **203266 (182502 , 226868)** | **8196.6 (7514.7 , 8939.5)** | **312676 (288085 , 341648)** | **6611.1 (6049.1 , 7276.5)** | **-19.3 (-23.9 , -14.4)** |
| **El Salvador** | **481698 (436519 , 534950)** | **11231.8 (10316.8 , 12381.5)** | **521102 (482613 , 561788)** | **8554.2 (7892.1 , 9219.3)** | **-23.8 (-28.4 , -19)** |
| **Guatemala** | **1147695 (1045775 , 1262861)** | **18377 (17185.8 , 19779.3)** | **1312472 (1218381 , 1420514)** | **9655.1 (8971.9 , 10456)** | **-47.5 (-50.6 , -44.2)** |
| **Honduras** | **400132 (354228 , 450688)** | **9631.5 (8809.5 , 10434.6)** | **518922 (474751 , 567743)** | **6779 (6197.1 , 7410)** | **-29.6 (-33.9 , -24.4)** |
| **Mexico** | **5433519 (4942720 , 5997806)** | **8298.4 (7729.5 , 8991.6)** | **5767845 (5377708 , 6181249)** | **4993.8 (4657.1 , 5356.8)** | **-39.8 (-41.4 , -38.2)** |
| **Nicaragua** | **316494 (278684 , 360868)** | **8945.6 (8229.2 , 9779.9)** | **338991 (311397 , 370493)** | **6286.5 (5800.6 , 6833.3)** | **-29.7 (-33.9 , -24.8)** |
| **Panama** | **140786 (128307 , 156022)** | **7254.1 (6641.8 , 8016.2)** | **277707 (256198 , 301232)** | **6716.6 (6196.5 , 7292.9)** | **-7.4 (-13.5 , -0.6)** |
| **Venezuela (Bolivarian Republic of)** | **1129650 (1026415 , 1236723)** | **7740.4 (7199.5 , 8343.5)** | **1693459 (1567088 , 1831620)** | **6132.5 (5654.5 , 6647.9)** | **-20.8 (-25.8 , -16)** |
| **Andean Latin America** | **4049562 (3716825 , 4426418)** | **13157.8 (12242.1 , 14181.3)** | **5400792 (5034520 , 5784693)** | **9264.7 (8642.5 , 9952.4)** | **-29.6 (-32.8 , -26.5)** |
| **Bolivia (Plurinational State of)** | **715271 (648312 , 795073)** | **13162.7 (12138.2 , 14274.4)** | **886692 (813979 , 958361)** | **8679.6 (8017.5 , 9379)** | **-34.1 (-37.6 , -30)** |
| **Ecuador** | **902679 (840565 , 977930)** | **11209.6 (10450.6 , 11967.7)** | **1240810 (1165265 , 1327248)** | **7960.2 (7456.4 , 8523.9)** | **-29 (-32.7 , -25.2)** |
| **Peru** | **2431611 (2229657 , 2655802)** | **13991 (12889.8 , 15254.9)** | **3273290 (3005286 , 3551622)** | **9997.2 (9178.8 , 10859.7)** | **-28.5 (-33.5 , -23.9)** |
| **Caribbean** | **2620667 (2438155 , 2819483)** | **8542.4 (8014.1 , 9129.4)** | **3118752 (2920790 , 3345178)** | **6395 (5975.2 , 6872.2)** | **-25.1 (-27.4 , -22.9)** |
| **Antigua and Barbuda** | **4452 (4107 , 4864)** | **7733.2 (7158.5 , 8409.4)** | **5299 (4892 , 5734)** | **5962 (5513.3 , 6461)** | **-22.9 (-27.1 , -18.6)** |
| **Barbados** | **20612 (19005 , 22516)** | **7614.9 (7023.9 , 8282.2)** | **24410 (22423 , 26608)** | **6280.7 (5794.3 , 6844.9)** | **-17.5 (-22.1 , -12.8)** |
| **Belize** | **11922 (10941 , 13091)** | **8356.5 (7715.7 , 9053.9)** | **19725 (18252 , 21301)** | **6168.5 (5703.7 , 6677.1)** | **-26.2 (-30.3 , -21.9)** |
| **Bermuda** | **3676 (3384 , 3987)** | **6433.8 (5901.3 , 7007.7)** | **4097 (3750 , 4449)** | **4475.2 (4084.2 , 4867.1)** | **-30.4 (-34.1 , -26.6)** |
| **Bahamas** | **14529 (13464 , 15641)** | **7643.9 (7087.5 , 8243.9)** | **18121 (16780 , 19430)** | **5046.9 (4685.9 , 5454.1)** | **-34 (-37.7 , -30)** |
| **Cuba** | **751284 (698512 , 815323)** | **7428.5 (6895.5 , 8049.2)** | **977253 (903314 , 1071783)** | **6196.4 (5718.9 , 6780.6)** | **-16.6 (-21.8 , -11.5)** |
| **Dominica** | **5724 (5277 , 6230)** | **7819.9 (7231.2 , 8482.1)** | **4379 (4050 , 4775)** | **5617.4 (5210 , 6121.2)** | **-28.2 (-32.1 , -23.8)** |
| **Dominican Republic** | **480794 (437205 , 531410)** | **8030.8 (7405.6 , 8713.3)** | **610084 (563432 , 662271)** | **6186.1 (5711.8 , 6726.9)** | **-23 (-27.4 , -18.3)** |
| **Grenada** | **7919 (7279 , 8600)** | **9749.9 (8983.2 , 10625.1)** | **6986 (6449 , 7588)** | **6920.7 (6400.2 , 7509.3)** | **-29 (-32.8 , -24.8)** |
| **Guyana** | **44559 (41041 , 48606)** | **8312.6 (7701.2 , 9049)** | **38515 (35602 , 41780)** | **5957.7 (5511.1 , 6454.8)** | **-28.3 (-32.6 , -23.1)** |
| **Haiti** | **632608 (571756 , 698838)** | **12228.7 (11275.2 , 13314.8)** | **775940 (712088 , 839996)** | **8513.6 (7859.7 , 9239.9)** | **-30.4 (-33.5 , -26.3)** |
| **Jamaica** | **150372 (137832 , 165330)** | **7006.9 (6463.4 , 7636.8)** | **146078 (134243 , 157640)** | **5128.4 (4701.7 , 5580.6)** | **-26.8 (-31 , -22.1)** |
| **Puerto Rico** | **283435 (263583 , 308334)** | **7989.5 (7416.4 , 8672.9)** | **256907 (236242 , 281405)** | **4969.1 (4562.6 , 5423.2)** | **-37.8 (-41.3 , -33.9)** |
| **Saint Kitts and Nevis** | **3378 (3105 , 3692)** | **8893.4 (8234.3 , 9634.7)** | **3330 (3064 , 3598)** | **5792.5 (5338.9 , 6267.4)** | **-34.9 (-38.1 , -30.7)** |
| **Saint Lucia** | **8970 (8237 , 9752)** | **8131 (7514.6 , 8773.6)** | **11674 (10828 , 12520)** | **6258.1 (5785.6 , 6718.3)** | **-23 (-27 , -18.6)** |
| **Saint Vincent and the Grenadines** | **6430 (5934 , 6980)** | **7200.8 (6689.4 , 7785.7)** | **7131 (6561 , 7697)** | **5968.1 (5502 , 6438.5)** | **-17.1 (-22.3 , -12.1)** |
| **Suriname** | **21075 (19401 , 22750)** | **6687.4 (6151.7 , 7264.8)** | **29208 (26974 , 31495)** | **5151.7 (4759.4 , 5567.6)** | **-23 (-27.8 , -18.3)** |
| **Trinidad and Tobago** | **76133 (70427 , 81947)** | **7613.4 (7074.2 , 8167.9)** | **67788 (62485 , 72813)** | **4469.2 (4125.4 , 4818.4)** | **-41.3 (-44.9 , -37.3)** |
| **United States Virgin Islands** | **5495 (5005 , 6030)** | **5992.3 (5490.2 , 6551.3)** | **6181 (5635 , 6782)** | **4602.3 (4195.4 , 5030.9)** | **-23.2 (-26.8 , -19.6)** |
| **Tropical Latin America** | **14000618 (12949991 , 15155103)** | **11161.3 (10445.4 , 11929.9)** | **19310825 (18046876 , 20623868)** | **8460.9 (7903.8 , 9068.5)** | **-24.2 (-26.4 , -22.1)** |
| **Brazil** | **13710459 (12688056 , 14840851)** | **11251.4 (10524 , 12025.4)** | **18933430 (17708899 , 20223219)** | **8517.5 (7955.4 , 9131.3)** | **-24.3 (-26.5 , -22.2)** |
| **Paraguay** | **290159 (263382 , 319165)** | **7996.7 (7420.4 , 8662.4)** | **377395 (348761 , 408167)** | **6112.3 (5654 , 6607.1)** | **-23.6 (-27.9 , -18.9)** |
| **East Asia** | **74612742 (68677402 , 81354252)** | **6986.7 (6500.1 , 7556.2)** | **59443625 (55161107 , 63906555)** | **3890.1 (3564 , 4198.1)** | **-44.3 (-46.6 , -42.3)** |
| **China** | **71562012 (65892055 , 78093701)** | **6936.4 (6454.5 , 7501.7)** | **55835910 (51694091 , 60027605)** | **3789 (3471.3 , 4101.3)** | **-45.4 (-47.7 , -43.2)** |
| **Democratic People's Republic of Korea** | **1635897 (1490234 , 1801722)** | **8341.9 (7683.1 , 9044.2)** | **1574785 (1457399 , 1707012)** | **5971.7 (5521.6 , 6450.6)** | **-28.4 (-31.9 , -24.2)** |
| **Taiwan (Province of China)** | **1414833 (1300314 , 1544193)** | **8169.7 (7524.8 , 8935.2)** | **2032930 (1868304 , 2201140)** | **7140.3 (6522.2 , 7755.9)** | **-12.6 (-16.9 , -7.9)** |
| **Southeast Asia** | **36468619 (33424427 , 39872869)** | **8786.7 (8195 , 9416.6)** | **41981067 (39209801 , 44943426)** | **6822.8 (6382.8 , 7328.4)** | **-22.4 (-24.5 , -20)** |
| **Cambodia** | **1377202 (1244549 , 1536223)** | **14897.4 (13871.2 , 16050.8)** | **1299679 (1203892 , 1399581)** | **9317.7 (8639.9 , 10060.1)** | **-37.5 (-40.8 , -33.8)** |
| **Indonesia** | **13750680 (12521714 , 15147834)** | **8345.6 (7724.2 , 8979.2)** | **15385208 (14243362 , 16649636)** | **6812.2 (6324.9 , 7374.6)** | **-18.4 (-21.7 , -14.7)** |
| **Lao People's Democratic Republic** | **486839 (438914 , 542571)** | **12308 (11359.5 , 13312.3)** | **424773 (391398 , 460367)** | **7281.9 (6767.6 , 7870.5)** | **-40.8 (-43.9 , -37.4)** |
| **Malaysia** | **1303094 (1195346 , 1426485)** | **8987.5 (8317.7 , 9703.4)** | **2432943 (2258482 , 2622645)** | **8681.6 (8061.9 , 9349.2)** | **-3.4 (-8.3 , 2.4)** |
| **Maldives** | **14161 (12698 , 15993)** | **7300.5 (6750.2 , 7917.5)** | **20746 (18783 , 22983)** | **4952.6 (4542.8 , 5422.2)** | **-32.2 (-35.9 , -28.2)** |
| **Mauritius** | **75762 (69678 , 82412)** | **8127.3 (7498.3 , 8836.1)** | **68788 (63557 , 74277)** | **5138.1 (4725.3 , 5613.6)** | **-36.8 (-40.6 , -33.2)** |
| **Myanmar** | **3679904 (3351533 , 4054792)** | **10015.4 (9228.2 , 10819.2)** | **2852127 (2642209 , 3062876)** | **5801 (5399.8 , 6228.9)** | **-42.1 (-45.4 , -38.6)** |
| **Philippines** | **6759276 (6197791 , 7405259)** | **11874.3 (11115 , 12744.7)** | **8487200 (7933826 , 9057742)** | **9169.7 (8610.6 , 9782.7)** | **-22.8 (-24.9 , -20.4)** |
| **Sri Lanka** | **1141810 (1052028 , 1234757)** | **7889.5 (7316.7 , 8484.3)** | **1172757 (1084000 , 1265708)** | **5238.7 (4832.3 , 5659.4)** | **-33.6 (-37.4 , -29.7)** |
| **Seychelles** | **6433 (5930 , 6975)** | **9793.1 (9066 , 10630.8)** | **8686 (8035 , 9449)** | **8492.7 (7877.3 , 9224)** | **-13.3 (-17.6 , -8.7)** |
| **Thailand** | **3098741 (2841913 , 3366200)** | **6480.7 (5975 , 6969.2)** | **4783008 (4393263 , 5156466)** | **5834.7 (5397.8 , 6287.1)** | **-10 (-15.4 , -4.7)** |
| **Timor-Leste** | **82097 (72528 , 92327)** | **10969.8 (10136.8 , 11896.7)** | **81114 (74472 , 88260)** | **7264 (6735.5 , 7828.3)** | **-33.8 (-37.7 , -29.9)** |
| **Viet Nam** | **4644146 (4178038 , 5145738)** | **7524.9 (6900.2 , 8133.7)** | **4909038 (4531029 , 5302374)** | **5488.2 (5053.4 , 5931.1)** | **-27.1 (-31 , -23.1)** |
| **Oceania** | **548626 (499680 , 607148)** | **9638.9 (8959.3 , 10351.8)** | **890935 (810411 , 978441)** | **7737.6 (7170.1 , 8360.6)** | **-19.7 (-22.2 , -17.1)** |
| **American Samoa** | **3050 (2779 , 3336)** | **7311.1 (6756.8 , 7871.6)** | **3052 (2803 , 3305)** | **5957.7 (5484.7 , 6432.2)** | **-18.5 (-22.3 , -14.5)** |
| **Cook Islands** | **1750 (1612 , 1889)** | **10120 (9401.5 , 10866.8)** | **1366 (1272 , 1467)** | **6888.8 (6415.3 , 7422.9)** | **-31.9 (-35.9 , -27.9)** |
| **Micronesia (Federated States of)** | **9263 (8443 , 10239)** | **9980.4 (9280.1 , 10801.8)** | **6478 (5984 , 6961)** | **7352.7 (6833 , 7868.3)** | **-26.3 (-30.3 , -22)** |
| **Fiji** | **51575 (46880 , 56588)** | **7932.1 (7334.6 , 8565.4)** | **49600 (45778 , 53361)** | **6028.3 (5602.9 , 6457)** | **-24 (-27.8 , -20.1)** |
| **Guam** | **7458 (6812 , 8154)** | **6489.2 (5991.9 , 7006.6)** | **9200 (8539 , 9929)** | **5259.6 (4875.4 , 5682.7)** | **-18.9 (-23.1 , -14.8)** |
| **Kiribati** | **5800 (5265 , 6359)** | **8569.2 (7964.3 , 9237.2)** | **6920 (6341 , 7496)** | **6813.6 (6314.6 , 7334.3)** | **-20.5 (-24.8 , -16.1)** |
| **Marshall Islands** | **3795 (3459 , 4205)** | **9776.7 (9098.6 , 10545)** | **3747 (3452 , 4062)** | **7825.2 (7259.9 , 8420.3)** | **-20 (-23.8 , -16.4)** |
| **Nauru** | **804 (729 , 885)** | **8723.9 (8084 , 9403.6)** | **624 (569 , 687)** | **7193.8 (6672.5 , 7766.8)** | **-17.5 (-21.6 , -13.2)** |
| **Niue** | **186 (171 , 201)** | **7827.5 (7246.5 , 8451.9)** | **110 (102 , 119)** | **6195.8 (5727.7 , 6676.3)** | **-20.8 (-24.6 , -17.2)** |
| **Northern Mariana Islands** | **2646 (2399 , 2896)** | **7354.2 (6764.3 , 7889.7)** | **2424 (2235 , 2627)** | **5845.8 (5417.3 , 6292.1)** | **-20.5 (-24.1 , -16.2)** |
| **Palau** | **1829 (1701 , 1972)** | **13345.1 (12490.3 , 14332.5)** | **1802 (1681 , 1944)** | **10270.8 (9609.8 , 11004.8)** | **-23 (-26.8 , -18.8)** |
| **Papua New Guinea** | **353810 (317955 , 397637)** | **9720.2 (8964.9 , 10552)** | **669186 (600331 , 741417)** | **7894 (7283.3 , 8602.6)** | **-18.8 (-22.2 , -15.1)** |
| **Samoa** | **13467 (12384 , 14656)** | **9450.6 (8794.5 , 10124.8)** | **13898 (12881 , 14956)** | **7564.8 (7030.9 , 8120.3)** | **-20 (-24.2 , -15.6)** |
| **Solomon Islands** | **42182 (38758 , 46184)** | **15395.4 (14412 , 16533.2)** | **54675 (49968 , 59846)** | **10655.8 (9919.3 , 11426.9)** | **-30.8 (-34.7 , -26.7)** |
| **Tokelau** | **150 (137 , 165)** | **8759.9 (8118 , 9458.6)** | **87 (80 , 94)** | **6343.3 (5838.5 , 6799.1)** | **-27.6 (-31.2 , -24.1)** |
| **Tonga** | **7811 (7158 , 8516)** | **9537.7 (8835.9 , 10233.5)** | **6266 (5799 , 6798)** | **6632.4 (6155.8 , 7159.4)** | **-30.5 (-34.2 , -26.3)** |
| **Tuvalu** | **844 (769 , 927)** | **9467 (8701.6 , 10226)** | **697 (646 , 750)** | **6449.5 (5997.4 , 6927.4)** | **-31.9 (-35.7 , -27.9)** |
| **Vanuatu** | **11844 (10777 , 13040)** | **9045.4 (8350.9 , 9783)** | **18721 (17219 , 20393)** | **7401.7 (6858.1 , 7947)** | **-18.2 (-22.2 , -13.8)** |
| **North Africa and Middle East** | **30314474 (27395800 , 33634682)** | **9152.7 (8478 , 9875.2)** | **34197034 (31709280 , 36805894)** | **6510.2 (6063.6 , 6997.8)** | **-28.9 (-30.8 , -26.8)** |
| **Afghanistan** | **1311680 (1167335 , 1475599)** | **11025.2 (10044 , 12043.3)** | **2546222 (2292974 , 2842980)** | **8037.7 (7400.8 , 8709.2)** | **-27.1 (-31 , -23.2)** |
| **Algeria** | **1828334 (1659833 , 2026974)** | **8032 (7409.5 , 8671)** | **2165039 (1997696 , 2340118)** | **5848.9 (5413.5 , 6308.4)** | **-27.2 (-30.8 , -23.3)** |
| **Bahrain** | **27377 (23866 , 31105)** | **7025 (6318.2 , 7734.1)** | **60374 (54753 , 66400)** | **5691.8 (5145.7 , 6240.1)** | **-19 (-22.9 , -14.9)** |
| **Egypt** | **6886857 (6191167 , 7672720)** | **12276.1 (11297.1 , 13351.4)** | **6998178 (6432818 , 7588299)** | **8150.8 (7535.8 , 8783.5)** | **-33.6 (-37.4 , -29.5)** |
| **Iran (Islamic Republic of)** | **4176991 (3769247 , 4629194)** | **7877.7 (7322.9 , 8489.8)** | **3945043 (3660046 , 4245822)** | **5193 (4827.6 , 5599)** | **-34.1 (-36.1 , -32)** |
| **Iraq** | **1593080 (1416257 , 1804018)** | **8878.5 (8122.5 , 9746.4)** | **1971734 (1801984 , 2166866)** | **5533.1 (5103 , 5988.9)** | **-37.7 (-41.2 , -33.4)** |
| **Jordan** | **259966 (233475 , 291468)** | **8161 (7540.1 , 8829.6)** | **534335 (483138 , 592746)** | **5571.8 (5111.3 , 6090.9)** | **-31.7 (-35.4 , -27.7)** |
| **Kuwait** | **97420 (87288 , 108853)** | **7268.2 (6687.6 , 7916.5)** | **211005 (193582 , 228655)** | **6583.2 (6041.4 , 7204.4)** | **-9.4 (-13.9 , -4.6)** |
| **Lebanon** | **215206 (193820 , 236683)** | **6939.8 (6373.8 , 7526.1)** | **285941 (261734 , 313270)** | **5578.4 (5103 , 6122.1)** | **-19.6 (-23.6 , -15.1)** |
| **Libya** | **311803 (278717 , 349293)** | **8019.8 (7332.7 , 8723.7)** | **340992 (313182 , 368631)** | **5983.2 (5508 , 6470.7)** | **-25.4 (-29.3 , -21.4)** |
| **Morocco** | **2175758 (1968273 , 2404930)** | **9051.7 (8317.6 , 9805.9)** | **2076477 (1920514 , 2241727)** | **6390 (5915.2 , 6889.9)** | **-29.4 (-33 , -25.5)** |
| **Palestine** | **222731 (200242 , 249012)** | **10827.5 (10019.9 , 11692.6)** | **283639 (256822 , 312565)** | **6940.6 (6422 , 7502.6)** | **-35.9 (-39.2 , -31.9)** |
| **Oman** | **148099 (132084 , 167490)** | **9391.8 (8601.9 , 10218.9)** | **183191 (166881 , 200389)** | **6261.6 (5738 , 6794.3)** | **-33.3 (-37.1 , -29.3)** |
| **Qatar** | **21976 (19709 , 24550)** | **6792.8 (6211.8 , 7389.7)** | **97218 (86992 , 108186)** | **5451.1 (4951.9 , 5981.8)** | **-19.8 (-24 , -15)** |
| **Saudi Arabia** | **1291892 (1164840 , 1437455)** | **9985.5 (9195.2 , 10796.9)** | **1876500 (1710127 , 2038350)** | **7105.3 (6499.8 , 7710.9)** | **-28.8 (-33 , -24.7)** |
| **Sudan** | **1869756 (1671042 , 2119517)** | **9254.6 (8502.6 , 10070.5)** | **2026310 (1841827 , 2229585)** | **6068.4 (5584.8 , 6548.2)** | **-34.4 (-38 , -30.8)** |
| **Syrian Arab Republic** | **1059413 (941304 , 1195988)** | **8055.4 (7402.5 , 8758.6)** | **850901 (784572 , 918579)** | **6535.3 (6016.6 , 7047.6)** | **-18.9 (-23 , -14.6)** |
| **Tunisia** | **617594 (559515 , 684461)** | **7931.3 (7310.6 , 8629.5)** | **687208 (628667 , 745739)** | **6097.2 (5586.3 , 6630)** | **-23.1 (-27.8 , -18.7)** |
| **Turkey** | **4436875 (4005030 , 4936842)** | **7949.7 (7278.5 , 8662.2)** | **4701615 (4354663 , 5083915)** | **5905.6 (5446.4 , 6416.4)** | **-25.7 (-30.6 , -20.8)** |
| **United Arab Emirates** | **89826 (80250 , 100655)** | **8203.1 (7545.1 , 8902.6)** | **319942 (288108 , 353471)** | **6584.1 (6045 , 7131.7)** | **-19.7 (-24.2 , -15.1)** |
| **Yemen** | **1651451 (1456473 , 1879830)** | **11786.1 (10816.9 , 12821.9)** | **2000425 (1822340 , 2186765)** | **8044.8 (7474.8 , 8718.3)** | **-31.7 (-35.3 , -27.9)** |
| **South Asia** | **126525782 (117272787 , 137058016)** | **14039 (13106.9 , 15033)** | **174927531 (163067794 , 187409925)** | **10998.7 (10265.9 , 11794.2)** | **-21.7 (-23.5 , -19.9)** |
| **Bangladesh** | **12207626 (11151211 , 13266555)** | **13265.1 (12353.2 , 14267.8)** | **10732029 (9912615 , 11534385)** | **7388.7 (6845.9 , 7924.8)** | **-44.3 (-47.6 , -40.6)** |
| **Bhutan** | **52697 (48087 , 58232)** | **10355.1 (9550.2 , 11219.3)** | **43455 (39930 , 47058)** | **6594.2 (6060.2 , 7187.1)** | **-36.3 (-39.6 , -32.7)** |
| **India** | **102993801 (95319429 , 111537685)** | **14902.4 (13894.7 , 15979)** | **148347208 (138305022 , 159263464)** | **11862.1 (11087 , 12749)** | **-20.4 (-22.3 , -18.5)** |
| **Nepal** | **2443898 (2224099 , 2689564)** | **14506.5 (13433.5 , 15706.1)** | **2400013 (2192110 , 2604378)** | **8925.8 (8158.4 , 9668.5)** | **-38.5 (-42 , -34.8)** |
| **Pakistan** | **8827760 (8072607 , 9726324)** | **8693 (8095.9 , 9346.6)** | **13404825 (12323639 , 14638414)** | **7246.9 (6737.5 , 7769.7)** | **-16.6 (-19.2 , -14.1)** |
| **Southern Sub-Saharan Africa** | **5025953 (4640257 , 5422149)** | **11199.1 (10471.7 , 11991)** | **6511041 (6081425 , 6958181)** | **9769.2 (9146.1 , 10445.3)** | **-12.8 (-14.9 , -10.7)** |
| **Botswana** | **103332 (94727 , 112992)** | **9869.7 (9141.1 , 10578.3)** | **155531 (142162 , 168731)** | **8184.4 (7559 , 8846.3)** | **-17.1 (-21.1 , -12.7)** |
| **Lesotho** | **176524 (161950 , 193610)** | **11568.4 (10700.7 , 12502.5)** | **181191 (166869 , 196250)** | **10237.9 (9467 , 11024.1)** | **-11.5 (-15.7 , -6.7)** |
| **Namibia** | **169049 (154508 , 185780)** | **14163.3 (13126.4 , 15325.8)** | **196261 (180980 , 213731)** | **9969.2 (9234 , 10800.5)** | **-29.6 (-33.3 , -25.9)** |
| **South Africa** | **3716043 (3431711 , 4003761)** | **11495.9 (10729.4 , 12300.5)** | **4835818 (4516637 , 5157560)** | **9907.6 (9252.7 , 10601.2)** | **-13.8 (-16.6 , -11.1)** |
| **Eswatini** | **76164 (68731 , 84727)** | **11463.5 (10626.9 , 12409)** | **83692 (77230 , 90797)** | **9016.9 (8403.2 , 9710.1)** | **-21.3 (-24.8 , -17.1)** |
| **Zimbabwe** | **784839 (719284 , 858621)** | **10002.1 (9332.1 , 10709.6)** | **1058548 (975883 , 1152308)** | **9184.2 (8553.7 , 9811.3)** | **-8.2 (-12.6 , -3.6)** |
| **Western Sub-Saharan Africa** | **19208920 (17506550 , 21162860)** | **11648.9 (10887.5 , 12495.6)** | **30715183 (28374576 , 33283614)** | **9378.5 (8770.2 , 10023.3)** | **-19.5 (-21 , -18)** |
| **Benin** | **472519 (427402 , 521088)** | **11670.7 (10839.7 , 12524.4)** | **839748 (770135 , 905707)** | **9484.8 (8824.3 , 10170.6)** | **-18.7 (-23 , -14.2)** |
| **Burkina Faso** | **918978 (832130 , 1014841)** | **11684.4 (10829.5 , 12614.1)** | **1630301 (1488039 , 1777082)** | **9973.6 (9256.3 , 10824.9)** | **-14.6 (-18.9 , -9.6)** |
| **Cameroon** | **906115 (820233 , 995202)** | **10809.3 (10005.1 , 11692.4)** | **1889858 (1740099 , 2046762)** | **9360.8 (8680.9 , 10027.8)** | **-13.4 (-17.9 , -9)** |
| **Cabo Verde** | **31590 (28771 , 34359)** | **9900.9 (9137.9 , 10638.2)** | **40212 (37182 , 43326)** | **8148.6 (7556.2 , 8802.1)** | **-17.7 (-22.5 , -12.8)** |
| **Chad** | **689096 (623106 , 765051)** | **13486.7 (12469.5 , 14648.5)** | **1461343 (1329212 , 1618933)** | **12208.1 (11289.3 , 13202.5)** | **-9.5 (-13.6 , -4.4)** |
| **CÃ´te d'Ivoire** | **1106011 (1002382 , 1222004)** | **12090.9 (11198 , 13027.3)** | **1924778 (1768955 , 2099416)** | **10488.8 (9688 , 11343.8)** | **-13.3 (-17.8 , -8.6)** |
| **Gambia** | **108935 (99095 , 119942)** | **13877.7 (12902.3 , 14970.5)** | **163964 (151219 , 177957)** | **10409.7 (9626.4 , 11173.7)** | **-25 (-29.2 , -20.6)** |
| **Ghana** | **1354318 (1236621 , 1480972)** | **11853.3 (10994.9 , 12775.8)** | **2258000 (2081983 , 2439972)** | **9751.3 (9020.1 , 10535.1)** | **-17.7 (-21.9 , -12.8)** |
| **Guinea** | **760526 (685057 , 841410)** | **13745.7 (12715.9 , 14906.7)** | **1144045 (1053306 , 1240540)** | **12390.4 (11495.5 , 13332.8)** | **-9.9 (-14.6 , -5.3)** |
| **Guinea-Bissau** | **106665 (95905 , 117545)** | **13400.2 (12418.9 , 14487.1)** | **138799 (127019 , 149892)** | **10694.3 (9888.3 , 11505.3)** | **-20.2 (-24.7 , -15.7)** |
| **Liberia** | **233170 (212273 , 260385)** | **13332.3 (12374.2 , 14448.5)** | **338221 (309913 , 366589)** | **10117.8 (9370.9 , 10969)** | **-24.1 (-28.8 , -19.2)** |
| **Mali** | **672473 (607985 , 744865)** | **8953.1 (8280.7 , 9692.5)** | **1203678 (1102278 , 1315929)** | **7271.9 (6770.2 , 7833.5)** | **-18.8 (-22.6 , -14.2)** |
| **Mauritania** | **194494 (177177 , 213106)** | **11512.6 (10650.3 , 12362.8)** | **285827 (262700 , 309996)** | **9483.2 (8741.2 , 10207.2)** | **-17.6 (-22.1 , -12.7)** |
| **Niger** | **999867 (899972 , 1114947)** | **14711.2 (13617.2 , 15865.4)** | **1880787 (1717601 , 2073066)** | **11575.7 (10739.5 , 12557.6)** | **-21.3 (-25.9 , -16.6)** |
| **Nigeria** | **9076719 (8256505 , 10057317)** | **11279.4 (10524.5 , 12114.8)** | **13156661 (12127729 , 14307817)** | **8545.2 (7993 , 9141.4)** | **-24.2 (-26.2 , -22.3)** |
| **Sao Tome and Principe** | **15194 (13851 , 16569)** | **14363.8 (13327.9 , 15420)** | **17464 (16137 , 18891)** | **11245.5 (10413.7 , 12120.5)** | **-21.7 (-26.1 , -16.4)** |
| **Senegal** | **745786 (675078 , 816731)** | **11463.6 (10622.3 , 12348.1)** | **1076028 (990796 , 1162464)** | **9311.4 (8662.4 , 10005.5)** | **-18.8 (-23.3 , -14.1)** |
| **Sierra Leone** | **437335 (397360 , 477676)** | **13773.5 (12716.3 , 14917.8)** | **707131 (650380 , 769981)** | **11735.8 (10862.8 , 12711.2)** | **-14.8 (-19.1 , -9.9)** |
| **Togo** | **378486 (343404 , 418879)** | **13213.2 (12242.1 , 14205.3)** | **557909 (511871 , 605206)** | **10007.6 (9259.8 , 10826)** | **-24.3 (-28.5 , -19.9)** |
| **Eastern Sub-Saharan Africa** | **20189808 (18438990 , 22242872)** | **12213.3 (11437.9 , 13037)** | **25993854 (24041397 , 28307585)** | **8453.3 (7929.8 , 9020.5)** | **-30.8 (-32.1 , -29.4)** |
| **Burundi** | **588933 (534526 , 647521)** | **12257.4 (11394.6 , 13195.3)** | **784788 (714588 , 861926)** | **8786.3 (8082 , 9474.1)** | **-28.3 (-32.1 , -24.5)** |
| **Comoros** | **55472 (50252 , 61205)** | **13186 (12215.4 , 14189.5)** | **51088 (47311 , 55242)** | **8314.5 (7716.9 , 8951.8)** | **-36.9 (-40 , -33.5)** |
| **Djibouti** | **44075 (39538 , 49223)** | **10651.3 (9884 , 11472.4)** | **71588 (65730 , 77696)** | **7694 (7125.2 , 8274.1)** | **-27.8 (-31.4 , -23.6)** |
| **Eritrea** | **366967 (325083 , 411769)** | **14295 (13135.1 , 15514.2)** | **532837 (485031 , 587095)** | **10521.3 (9727.5 , 11440.7)** | **-26.4 (-29.8 , -22.9)** |
| **Ethiopia** | **6010904 (5467802 , 6648644)** | **13619.8 (12640.9 , 14588.7)** | **6628674 (6108786 , 7230986)** | **8313.8 (7757.7 , 8918)** | **-39 (-41 , -36.5)** |
| **Kenya** | **1920140 (1757578 , 2109186)** | **10334.1 (9672.8 , 11035.6)** | **3044843 (2818230 , 3288833)** | **8426.5 (7895.3 , 9005.5)** | **-18.5 (-19.9 , -17.1)** |
| **Madagascar** | **1495040 (1374161 , 1633721)** | **13561.4 (12673.6 , 14561.4)** | **1888566 (1730618 , 2055472)** | **9371.9 (8687.6 , 10093.6)** | **-30.9 (-35.1 , -26)** |
| **Malawi** | **969025 (874548 , 1093999)** | **11561.8 (10729.8 , 12502.9)** | **1077776 (986971 , 1181353)** | **7952.7 (7379 , 8569.6)** | **-31.2 (-34.7 , -27.4)** |
| **Mozambique** | **1208292 (1087213 , 1348830)** | **10291.5 (9473.3 , 11172.6)** | **1587812 (1450812 , 1739829)** | **7458.1 (6903.9 , 8015.8)** | **-27.5 (-31.6 , -23.4)** |
| **Rwanda** | **937922 (845507 , 1048152)** | **14262.5 (13213.7 , 15440.2)** | **839557 (769234 , 914466)** | **8643.6 (7980 , 9247.1)** | **-39.4 (-42.5 , -36.1)** |
| **Somalia** | **747615 (673118 , 835825)** | **12294.6 (11400.5 , 13309)** | **1531183 (1390485 , 1706148)** | **9849.2 (9163.4 , 10669)** | **-19.9 (-24.3 , -15.7)** |
| **South Sudan** | **569367 (513451 , 629875)** | **11393.1 (10531.2 , 12331)** | **642925 (586970 , 704914)** | **8807 (8173.2 , 9512.2)** | **-22.7 (-26.5 , -18.8)** |
| **United Republic of Tanzania** | **2938636 (2652982 , 3247971)** | **12791.8 (11891.1 , 13784.7)** | **3906504 (3565308 , 4259103)** | **8633.8 (8021.9 , 9257.3)** | **-32.5 (-35.9 , -28.5)** |
| **Uganda** | **1575470 (1420444 , 1765099)** | **10276.3 (9513.9 , 11109.5)** | **2481027 (2270761 , 2733429)** | **8161.1 (7566.6 , 8844.6)** | **-20.6 (-24.6 , -16.2)** |
| **Zambia** | **747135 (674594 , 832104)** | **10698.2 (9893.7 , 11504.7)** | **903905 (827501 , 992197)** | **6912.9 (6404.6 , 7402.7)** | **-35.4 (-38.5 , -31.7)** |
| **Central Sub-Saharan Africa** | **6530799 (5921535 , 7284306)** | **14148.7 (13165.8 , 15230.5)** | **8867737 (8139554 , 9680999)** | **9459.2 (8762.8 , 10213.9)** | **-33.1 (-35.7 , -30.4)** |
| **Angola** | **1167071 (1047457 , 1312302)** | **13259.5 (12224 , 14312.7)** | **1587322 (1465420 , 1734093)** | **7412.2 (6899 , 7945.4)** | **-44.1 (-47.3 , -40.7)** |
| **Central African Republic** | **296190 (267566 , 327228)** | **13028.7 (12067.8 , 13964.4)** | **436616 (400856 , 481358)** | **10972.8 (10124.1 , 11933.8)** | **-15.8 (-20.1 , -11.2)** |
| **Congo** | **226961 (206022 , 250082)** | **11396.8 (10505.3 , 12270.7)** | **306707 (282110 , 333334)** | **7987.2 (7378.2 , 8603.2)** | **-29.9 (-33.6 , -26.4)** |
| **Democratic Republic of the Congo** | **4688581 (4223908 , 5240967)** | **14739 (13640 , 15939.6)** | **6366713 (5806249 , 6953648)** | **10188.3 (9382.9 , 11045.9)** | **-30.9 (-34.1 , -27.2)** |
| **Equatorial Guinea** | **55634 (49942 , 62188)** | **14507.1 (13426.1 , 15732.7)** | **63117 (57815 , 68990)** | **6866.3 (6347.2 , 7381.5)** | **-52.7 (-55.8 , -49.3)** |
| **Gabon** | **96362 (87646 , 105904)** | **11453.4 (10562.3 , 12341)** | **107262 (99313 , 115601)** | **7899.9 (7320.2 , 8502.6)** | **-31 (-34.8 , -27)** |
