# Supplementary material for: Global burden of lower respiratory infections during the last three decades
Source: Front Public Health. 2023 Jan 9;10:1028525. doi: 10.3389/fpubh.2022.1028525 (PMC9869262; doi:10.3389/fpubh.2022.1028525)
Supplement: Table S3 — Deaths from lower respiratory infections in 1990 and 2019 and the percentage change in the age-standardised rates (ASRs) per 100,000, by location (generated from data available from http://ghdx.healthdata.org/gbd-results-tool). [file Table_3.DOC]

| **Table S3: Deaths from lower respiratory infections in 1990 and 2019 and the percentage change in the age-standardised rates (ASRs) per 100,000, by location**  **(Generated from data available from http://ghdx.healthdata.org/gbd-results-tool)** | | | | | |
| --- | --- | --- | --- | --- | --- |
|  | **1990** | | **2019** | | **Percentage change in ASRs per 100,000** |
|  | **No. (95% UI)** | **ASRs per 100,000 (95% UI)** | **No. (95% UI)** | **ASRs per 100,000 (95% UI)** |
| **Global** | **3320008 (3018493 , 3715057)** | **66.7 (61.4 , 72.9)** | **2493200 (2268183 , 2736184)** | **34.3 (31.1 , 37.9)** | **-48.5 (-54 , -42.9)** |
| **High-income North America** | **79307 (72163 , 83294)** | **21.9 (19.9 , 22.9)** | **91060 (79893 , 97147)** | **13.2 (11.7 , 14)** | **-39.6 (-41.5 , -37.8)** |
| **Canada** | **6576 (5899 , 6987)** | **21.8 (19.4 , 23.2)** | **9125 (7574 , 10250)** | **11.6 (9.8 , 12.9)** | **-46.9 (-51.5 , -42.2)** |
| **Greenland** | **13 (10 , 15)** | **48.9 (38.2 , 56)** | **12 (10 , 15)** | **25.3 (20.3 , 29.9)** | **-48.3 (-58.2 , -36.5)** |
| **United States of America** | **72716 (66216 , 76436)** | **21.9 (20 , 23)** | **81921 (72236 , 87404)** | **13.4 (12 , 14.2)** | **-38.9 (-40.7 , -36.9)** |
| **Australasia** | **2819 (2549 , 2992)** | **13.9 (12.4 , 14.9)** | **5164 (4271 , 5780)** | **8.9 (7.5 , 9.9)** | **-36.1 (-41.7 , -30.9)** |
| **Australia** | **1823 (1650 , 1945)** | **10.7 (9.6 , 11.5)** | **4269 (3503 , 4810)** | **8.7 (7.3 , 9.8)** | **-18.3 (-26.9 , -10.4)** |
| **New Zealand** | **996 (882 , 1084)** | **29.4 (25.8 , 32.2)** | **896 (729 , 1031)** | **9.8 (8.1 , 11.2)** | **-66.7 (-70.7 , -62.4)** |
| **High-income Asia Pacific** | **68749 (63035 , 71460)** | **42.2 (38.2 , 44.2)** | **137989 (110299 , 154096)** | **22.1 (18.3 , 24.4)** | **-47.5 (-52.7 , -44.2)** |
| **Brunei Darussalam** | **33 (28 , 40)** | **45.2 (38.8 , 61.5)** | **82 (68 , 94)** | **52.4 (44.3 , 60.1)** | **16 (-22.5 , 42)** |
| **Japan** | **62767 (57265 , 65307)** | **43.4 (39.1 , 45.4)** | **118801 (93666 , 133416)** | **22.3 (18.1 , 24.6)** | **-48.6 (-53.7 , -45.3)** |
| **Singapore** | **1292 (1196 , 1363)** | **79.4 (72 , 84.4)** | **3471 (2900 , 3822)** | **50.3 (41.8 , 55.4)** | **-36.6 (-42.4 , -31.4)** |
| **Republic of Korea** | **4657 (4312 , 5274)** | **23 (20.6 , 27.7)** | **15635 (8906 , 18031)** | **19.8 (11.4 , 22.9)** | **-13.9 (-58 , 0.1)** |
| **Western Europe** | **123637 (113224 , 128967)** | **22.1 (20.1 , 23.1)** | **159075 (136430 , 171243)** | **13.7 (11.9 , 14.7)** | **-37.8 (-40.8 , -35.2)** |
| **Andorra** | **6 (4 , 8)** | **17 (13.1 , 21.9)** | **20 (15 , 25)** | **12.3 (9.4 , 15.3)** | **-27.7 (-47.6 , -3.2)** |
| **Austria** | **1374 (1267 , 1454)** | **11.9 (10.9 , 12.6)** | **1205 (1031 , 1342)** | **5.6 (4.8 , 6.1)** | **-53.3 (-57.4 , -48.8)** |
| **Belgium** | **3460 (3145 , 3677)** | **22.9 (20.7 , 24.3)** | **6340 (5293 , 7068)** | **21.3 (18.1 , 23.5)** | **-6.9 (-15 , 1.4)** |
| **Cyprus** | **140 (114 , 161)** | **28.5 (23.5 , 32.4)** | **169 (144 , 206)** | **11.5 (9.8 , 13.9)** | **-59.5 (-66.3 , -47.3)** |
| **Denmark** | **1836 (1671 , 1955)** | **21.6 (19.7 , 23)** | **2473 (2055 , 2768)** | **18.5 (15.6 , 20.6)** | **-14.5 (-23.2 , -5.4)** |
| **Finland** | **2578 (2337 , 2737)** | **38.2 (34.4 , 40.7)** | **775 (657 , 872)** | **5.2 (4.5 , 5.8)** | **-86.4 (-87.7 , -85)** |
| **France** | **17669 (15949 , 19001)** | **20.2 (18.1 , 21.6)** | **23096 (18485 , 26342)** | **11.7 (9.6 , 13.1)** | **-42.1 (-48.5 , -36.4)** |
| **Germany** | **19090 (17388 , 20263)** | **14.9 (13.5 , 15.8)** | **25096 (21764 , 27833)** | **11 (9.7 , 12.1)** | **-26 (-32 , -19.4)** |
| **Greece** | **1893 (1743 , 2019)** | **14 (12.7 , 15)** | **5957 (5039 , 6635)** | **18.9 (16.3 , 20.8)** | **34.9 (22.3 , 48.1)** |
| **Iceland** | **101 (89 , 110)** | **33 (29 , 35.9)** | **102 (82 , 117)** | **14.6 (11.9 , 16.7)** | **-55.9 (-61.1 , -50.2)** |
| **Ireland** | **1864 (1732 , 1963)** | **52 (47.6 , 54.9)** | **1713 (1425 , 1925)** | **21.8 (18.2 , 24.5)** | **-58 (-62.8 , -53.7)** |
| **Israel** | **721 (660 , 770)** | **17.3 (15.5 , 18.5)** | **1739 (1488 , 1935)** | **13.4 (11.5 , 14.8)** | **-22.7 (-30.1 , -15.3)** |
| **Italy** | **8630 (8010 , 8992)** | **11.2 (10.3 , 11.8)** | **12393 (10346 , 13493)** | **6.6 (5.6 , 7.1)** | **-41.6 (-45.9 , -38.7)** |
| **Luxembourg** | **89 (80 , 96)** | **18.1 (16.3 , 19.6)** | **137 (111 , 159)** | **11.6 (9.5 , 13.5)** | **-35.9 (-46.3 , -25.3)** |
| **Malta** | **93 (84 , 101)** | **25.5 (22.9 , 27.6)** | **192 (158 , 220)** | **18.7 (15.6 , 21.5)** | **-26.5 (-36.6 , -15.5)** |
| **Monaco** | **15 (11 , 19)** | **18.5 (14.7 , 23.1)** | **25 (19 , 29)** | **19.5 (15.7 , 23)** | **5.4 (-21.3 , 32.8)** |
| **Netherlands** | **3966 (3479 , 4254)** | **20.1 (17.6 , 21.6)** | **6330 (5329 , 7117)** | **16.4 (13.9 , 18.4)** | **-18.5 (-26.3 , -10.3)** |
| **Norway** | **2979 (2650 , 3150)** | **39.3 (35 , 41.6)** | **2036 (1695 , 2233)** | **16.5 (13.9 , 17.9)** | **-58.2 (-60.5 , -55.5)** |
| **Portugal** | **3325 (3138 , 3502)** | **28.9 (27.2 , 30.6)** | **8279 (6926 , 9168)** | **27.5 (23.3 , 30.2)** | **-5 (-15.6 , 3.6)** |
| **San Marino** | **4 (3 , 4)** | **12.1 (10.1 , 14.3)** | **8 (5 , 10)** | **9.1 (6.4 , 12.2)** | **-24.5 (-47.6 , 4.7)** |
| **Spain** | **9015 (8154 , 9598)** | **18.4 (16.5 , 19.6)** | **14184 (11739 , 16134)** | **11 (9.4 , 12.3)** | **-40.2 (-45.8 , -34.2)** |
| **Sweden** | **4271 (3802 , 4609)** | **25.6 (22.8 , 27.5)** | **2846 (2390 , 3168)** | **10.5 (9 , 11.7)** | **-58.9 (-62.5 , -55.3)** |
| **Switzerland** | **2578 (2297 , 2760)** | **23.4 (20.7 , 25)** | **1946 (1588 , 2216)** | **8.4 (7 , 9.5)** | **-64 (-67.6 , -60.6)** |
| **United Kingdom** | **37837 (34491 , 39615)** | **42.7 (38.8 , 44.9)** | **41877 (36343 , 44864)** | **28 (24.5 , 29.9)** | **-34.5 (-37 , -31)** |
| **Southern Latin America** | **15892 (15042 , 16488)** | **38.3 (35.8 , 39.8)** | **42564 (37971 , 45844)** | **50.2 (45 , 54.1)** | **31.1 (22.4 , 40.1)** |
| **Argentina** | **8684 (8185 , 9085)** | **29.8 (27.8 , 31.3)** | **36264 (32278 , 39330)** | **65.7 (58.7 , 71.2)** | **120.5 (104 , 137.5)** |
| **Chile** | **6387 (6026 , 6661)** | **75.2 (69.6 , 78.7)** | **4703 (4035 , 5180)** | **20.4 (17.5 , 22.5)** | **-72.8 (-75.6 , -70.3)** |
| **Uruguay** | **820 (753 , 870)** | **23.3 (21.3 , 24.7)** | **1595 (1388 , 1761)** | **25.2 (22.1 , 27.6)** | **8.1 (-1.6 , 19.3)** |
| **Eastern Europe** | **31879 (30878 , 32910)** | **15.4 (14.9 , 16)** | **44838 (40209 , 49911)** | **15.6 (14 , 17.2)** | **1 (-9.5 , 11.9)** |
| **Belarus** | **1298 (1211 , 1386)** | **13 (12 , 14)** | **1155 (928 , 1436)** | **8.2 (6.5 , 10.2)** | **-37.2 (-49.6 , -21.2)** |
| **Estonia** | **219 (206 , 232)** | **13.1 (12.3 , 13.9)** | **224 (177 , 272)** | **9.3 (7.4 , 11.3)** | **-28.8 (-43.6 , -12.4)** |
| **Latvia** | **383 (362 , 404)** | **12.8 (12.1 , 13.5)** | **350 (295 , 418)** | **10.4 (8.8 , 12.4)** | **-18.6 (-32 , -3.1)** |
| **Lithuania** | **376 (351 , 397)** | **9.5 (8.8 , 10)** | **531 (443 , 637)** | **10.5 (8.7 , 12.4)** | **10.7 (-8 , 32.6)** |
| **Republic of Moldova** | **1213 (1118 , 1323)** | **30.8 (28.3 , 33.6)** | **872 (761 , 985)** | **19.2 (16.9 , 21.6)** | **-37.6 (-46.9 , -27.3)** |
| **Russian Federation** | **22422 (21708 , 23195)** | **16.7 (16.1 , 17.4)** | **32972 (28742 , 37603)** | **16.5 (14.4 , 18.7)** | **-1.3 (-13.5 , 12)** |
| **Ukraine** | **5968 (5670 , 6261)** | **11.4 (10.7 , 12.1)** | **8734 (7349 , 10415)** | **14.8 (12.6 , 17.6)** | **30 (8.4 , 56.6)** |
| **Central Europe** | **32736 (31158 , 33894)** | **28.6 (27.1 , 29.8)** | **30679 (27109 , 33943)** | **15.5 (13.7 , 17.1)** | **-45.8 (-51.9 , -39.8)** |
| **Albania** | **2140 (1543 , 2420)** | **74.8 (47.3 , 83.6)** | **457 (358 , 578)** | **14 (11.3 , 17.3)** | **-81.3 (-85.4 , -67.4)** |
| **Bosnia and Herzegovina** | **482 (419 , 519)** | **15.4 (13.2 , 16.7)** | **414 (326 , 542)** | **8.1 (6.4 , 10.4)** | **-47.7 (-59 , -24.9)** |
| **Bulgaria** | **4140 (3936 , 4344)** | **46.5 (44.1 , 49)** | **1990 (1601 , 2421)** | **16.7 (13.6 , 20.3)** | **-64.1 (-70.9 , -56.3)** |
| **Croatia** | **876 (812 , 933)** | **16.8 (15.5 , 17.9)** | **580 (464 , 709)** | **6.5 (5.3 , 8)** | **-61.2 (-68.5 , -53.1)** |
| **Czechia** | **2324 (2201 , 2431)** | **19.1 (18 , 20)** | **3386 (2788 , 4038)** | **16 (13.2 , 19.1)** | **-16.3 (-29.6 , -0.5)** |
| **Hungary** | **1301 (1237 , 1356)** | **11.8 (11.2 , 12.4)** | **1217 (1008 , 1449)** | **6.6 (5.5 , 7.9)** | **-44.3 (-53 , -33.7)** |
| **Montenegro** | **57 (50 , 65)** | **10.5 (9.3 , 11.9)** | **69 (57 , 84)** | **8.4 (7 , 10.2)** | **-20.4 (-35.1 , -3.7)** |
| **North Macedonia** | **354 (311 , 403)** | **21.6 (19.1 , 24.6)** | **181 (143 , 248)** | **8.5 (6.9 , 11)** | **-60.6 (-69.6 , -46.4)** |
| **Poland** | **6912 (6571 , 7148)** | **18.4 (17.3 , 19.1)** | **11910 (10014 , 13789)** | **16.9 (14.4 , 19.7)** | **-7.8 (-20.6 , 6.2)** |
| **Romania** | **9536 (9154 , 10079)** | **48.3 (46.1 , 51.6)** | **6362 (5323 , 7512)** | **21.5 (18.3 , 25.2)** | **-55.4 (-62.3 , -47.3)** |
| **Serbia** | **1305 (1163 , 1518)** | **15.6 (13.7 , 18.3)** | **1571 (1253 , 1925)** | **11.4 (9.1 , 13.7)** | **-27 (-43.7 , -7.7)** |
| **Slovakia** | **2785 (2012 , 3049)** | **51.3 (37.5 , 55.9)** | **1850 (1465 , 2261)** | **21.7 (17.2 , 26.5)** | **-57.7 (-67 , -42.6)** |
| **Slovenia** | **523 (418 , 641)** | **24 (19.3 , 29)** | **693 (537 , 867)** | **13 (10.2 , 16.3)** | **-45.8 (-59.7 , -28.8)** |
| **Central Asia** | **55735 (51043 , 61081)** | **65.6 (60.5 , 71.3)** | **24430 (21283 , 28467)** | **31.1 (27.5 , 35.6)** | **-52.6 (-59.1 , -44.9)** |
| **Armenia** | **1147 (1019 , 1284)** | **33.8 (30.4 , 37.6)** | **546 (466 , 629)** | **16.9 (14.5 , 19.4)** | **-50.1 (-58.4 , -39.8)** |
| **Azerbaijan** | **8617 (7447 , 9980)** | **99.5 (86.6 , 114.8)** | **2454 (1984 , 3008)** | **35.6 (29.2 , 43.2)** | **-64.3 (-71.8 , -55.1)** |
| **Georgia** | **1925 (1735 , 2140)** | **42.6 (38.1 , 47.6)** | **765 (643 , 888)** | **13.6 (11.7 , 15.7)** | **-68 (-72.7 , -62.5)** |
| **Kazakhstan** | **6313 (5758 , 6938)** | **39.4 (36.2 , 43)** | **4013 (3533 , 4554)** | **24.8 (22 , 28)** | **-37 (-44.8 , -27.1)** |
| **Kyrgyzstan** | **3332 (2997 , 3718)** | **60 (54.5 , 66.5)** | **772 (665 , 868)** | **13.8 (12.1 , 15.4)** | **-77.1 (-80.3 , -73.4)** |
| **Mongolia** | **3127 (2587 , 3626)** | **109.3 (88 , 124.3)** | **530 (401 , 678)** | **18.5 (14.8 , 23)** | **-83.1 (-86.9 , -75.5)** |
| **Tajikistan** | **7987 (6877 , 9079)** | **101.9 (90.6 , 113.7)** | **3279 (2586 , 4281)** | **45.3 (37.5 , 58.5)** | **-55.5 (-64.7 , -41.1)** |
| **Turkmenistan** | **4943 (4364 , 5596)** | **91.6 (81.7 , 102.2)** | **1284 (1032 , 1620)** | **26 (21.2 , 32.4)** | **-71.6 (-78.1 , -63.6)** |
| **Uzbekistan** | **18343 (16288 , 20519)** | **61.5 (55.4 , 67.7)** | **10787 (9095 , 12944)** | **40.1 (34.5 , 46.8)** | **-34.8 (-45.8 , -21.7)** |
| **Central Latin America** | **60268 (55958 , 64193)** | **47.9 (44.5 , 50.2)** | **51851 (44387 , 60174)** | **23 (19.7 , 26.8)** | **-51.8 (-58.8 , -44.1)** |
| **Colombia** | **7757 (7025 , 8500)** | **32 (29.5 , 34)** | **7899 (6078 , 9998)** | **15.1 (11.5 , 19.2)** | **-52.8 (-64.1 , -39.8)** |
| **Costa Rica** | **433 (390 , 484)** | **21.7 (19.4 , 23.6)** | **676 (525 , 843)** | **13.2 (10.2 , 16.4)** | **-39.2 (-52.2 , -23.1)** |
| **El Salvador** | **1906 (1690 , 2153)** | **45.5 (40.6 , 49.7)** | **2304 (1735 , 2908)** | **35.5 (26.8 , 45.2)** | **-21.8 (-41.4 , -0.3)** |
| **Guatemala** | **11129 (10110 , 12195)** | **213.8 (195.8 , 231.9)** | **9875 (7836 , 12296)** | **90 (72.5 , 109.3)** | **-57.9 (-66.1 , -48.3)** |
| **Honduras** | **1533 (1285 , 1825)** | **33.7 (28.3 , 38.7)** | **1121 (904 , 1553)** | **20 (16.3 , 26.8)** | **-40.6 (-52.2 , -18.6)** |
| **Mexico** | **31114 (27795 , 34039)** | **47.6 (44 , 50.5)** | **22592 (19670 , 25395)** | **21 (18.2 , 23.6)** | **-55.8 (-61.4 , -49.9)** |
| **Nicaragua** | **1915 (1596 , 2279)** | **41.8 (36.6 , 47.2)** | **1011 (854 , 1208)** | **23.9 (20.4 , 28.8)** | **-42.7 (-53 , -29.9)** |
| **Panama** | **379 (339 , 422)** | **21.8 (19.5 , 23.8)** | **818 (630 , 1026)** | **19.3 (14.8 , 24.2)** | **-11.7 (-30.9 , 11.5)** |
| **Venezuela (Bolivarian Republic of)** | **4104 (3836 , 4381)** | **33.4 (30.5 , 35.5)** | **5555 (4275 , 7110)** | **20.7 (15.9 , 26.5)** | **-38.1 (-52.3 , -21.2)** |
| **Andean Latin America** | **35375 (32137 , 39128)** | **110.7 (100.6 , 120.4)** | **30185 (24350 , 36816)** | **55.6 (44.9 , 67.5)** | **-49.8 (-59.2 , -39.3)** |
| **Bolivia (Plurinational State of)** | **10239 (8526 , 12302)** | **161.3 (141 , 184)** | **6570 (5405 , 7915)** | **80.5 (65.7 , 98.1)** | **-50.1 (-60.5 , -38.6)** |
| **Ecuador** | **4568 (4172 , 4984)** | **66.4 (60.5 , 71)** | **5436 (4436 , 6763)** | **41.9 (34.5 , 52)** | **-36.9 (-47.8 , -22.1)** |
| **Peru** | **20568 (18251 , 23216)** | **114.1 (101.5 , 126.7)** | **18179 (13604 , 23354)** | **55.1 (41.3 , 71)** | **-51.7 (-63.3 , -37.6)** |
| **Caribbean** | **17594 (15835 , 19592)** | **57.1 (52.4 , 62.3)** | **19182 (16476 , 22128)** | **39.3 (33.6 , 45.6)** | **-31.3 (-41.7 , -19.9)** |
| **Antigua and Barbuda** | **22 (19 , 24)** | **37 (33.1 , 40.8)** | **30 (25 , 35)** | **36.7 (31.2 , 42.6)** | **-0.8 (-14.6 , 16.3)** |
| **Barbados** | **87 (78 , 95)** | **30.2 (27.3 , 32.7)** | **164 (137 , 192)** | **35.5 (29.5 , 41.6)** | **17.5 (-1.7 , 38.4)** |
| **Belize** | **70 (63 , 78)** | **53.2 (47.9 , 58.3)** | **112 (96 , 129)** | **43.4 (37.1 , 50)** | **-18.5 (-30.1 , -4.4)** |
| **Bermuda** | **12 (11 , 14)** | **23.6 (21.2 , 25.9)** | **18 (15 , 22)** | **12.9 (10.6 , 15.8)** | **-45.3 (-54.1 , -33.3)** |
| **Bahamas** | **63 (57 , 69)** | **42.4 (38.2 , 46.5)** | **94 (78 , 112)** | **28.9 (24.2 , 34.4)** | **-31.8 (-43.3 , -18)** |
| **Cuba** | **3281 (3023 , 3452)** | **35 (31.8 , 36.9)** | **7125 (5818 , 8459)** | **35 (28.8 , 41.5)** | **0.1 (-16.7 , 18)** |
| **Dominica** | **28 (25 , 31)** | **38 (33.7 , 42.1)** | **30 (24 , 36)** | **35.4 (28.7 , 42.4)** | **-6.7 (-24.1 , 13.9)** |
| **Dominican Republic** | **2175 (1826 , 2577)** | **32.8 (28.8 , 37.5)** | **2010 (1585 , 2501)** | **22.6 (18 , 28.1)** | **-31.1 (-46.1 , -12.2)** |
| **Grenada** | **58 (52 , 64)** | **68.7 (61.8 , 75.8)** | **47 (41 , 53)** | **53.4 (46.7 , 59.8)** | **-22.3 (-33 , -11.2)** |
| **Guyana** | **274 (240 , 306)** | **65.8 (58.4 , 73.1)** | **262 (205 , 325)** | **51.3 (40.9 , 62.2)** | **-22 (-40 , -2.4)** |
| **Haiti** | **8644 (7152 , 10358)** | **132.9 (109.5 , 159.8)** | **6323 (4815 , 8027)** | **75.8 (56.7 , 97.6)** | **-42.9 (-56.1 , -28.4)** |
| **Jamaica** | **476 (434 , 518)** | **23.8 (21.6 , 25.8)** | **475 (378 , 578)** | **14.5 (11.5 , 17.9)** | **-39.1 (-50.4 , -25.4)** |
| **Puerto Rico** | **1269 (1167 , 1342)** | **38.3 (34.9 , 40.6)** | **1267 (988 , 1563)** | **16.1 (12.7 , 20.1)** | **-58 (-66.3 , -48.1)** |
| **Saint Kitts and Nevis** | **23 (21 , 25)** | **67.5 (61.4 , 73.7)** | **20 (17 , 23)** | **41 (35.2 , 47.3)** | **-39.2 (-48.7 , -28.3)** |
| **Saint Lucia** | **36 (33 , 38)** | **43.6 (39.9 , 47.1)** | **54 (45 , 63)** | **28.7 (24 , 33.6)** | **-34.1 (-44.3 , -22.5)** |
| **Saint Vincent and the Grenadines** | **30 (27 , 33)** | **41.1 (37.1 , 45)** | **39 (34 , 45)** | **34.5 (29.9 , 39.6)** | **-16.1 (-27.9 , -2.1)** |
| **Suriname** | **109 (96 , 121)** | **38.5 (34.4 , 42.2)** | **163 (134 , 196)** | **30.8 (25.2 , 36.8)** | **-20 (-33.6 , -3.7)** |
| **Trinidad and Tobago** | **339 (319 , 360)** | **45.5 (42.3 , 48.5)** | **274 (212 , 347)** | **17 (13.2 , 21.7)** | **-62.6 (-71 , -52.5)** |
| **United States Virgin Islands** | **13 (11 , 15)** | **19.5 (16.5 , 22.7)** | **25 (22 , 29)** | **16.5 (14.1 , 18.9)** | **-15.3 (-30.5 , 3.5)** |
| **Tropical Latin America** | **68759 (63375 , 76944)** | **64 (59.4 , 69.6)** | **90114 (79540 , 96039)** | **40.8 (35.8 , 43.5)** | **-36.4 (-42.9 , -31.2)** |
| **Brazil** | **67705 (62281 , 75840)** | **65.2 (60.4 , 70.9)** | **88641 (78255 , 94446)** | **41.1 (36.1 , 43.8)** | **-37 (-43.5 , -31.8)** |
| **Paraguay** | **1054 (921 , 1187)** | **32.4 (28.7 , 36.1)** | **1474 (1129 , 1852)** | **27.3 (20.8 , 34.4)** | **-15.8 (-35.1 , 5.9)** |
| **East Asia** | **560877 (487984 , 626965)** | **61.5 (53.8 , 67.2)** | **205330 (180262 , 238102)** | **14.6 (12.9 , 16.8)** | **-76.2 (-79.5 , -69.3)** |
| **China** | **543901 (472378 , 606600)** | **62 (54 , 67.6)** | **185264 (160993 , 216218)** | **13.8 (12.1 , 16)** | **-77.7 (-80.8 , -70.8)** |
| **Democratic People's Republic of Korea** | **13165 (10079 , 17850)** | **60.8 (49.8 , 74.9)** | **6998 (5763 , 8397)** | **28.1 (23.1 , 34)** | **-53.7 (-64.6 , -42.4)** |
| **Taiwan (Province of China)** | **3811 (3597 , 3990)** | **32.9 (30.3 , 34.7)** | **13067 (10457 , 16178)** | **32.2 (25.9 , 40.1)** | **-2 (-20.1 , 21.7)** |
| **Southeast Asia** | **273820 (234414 , 330878)** | **70 (62.2 , 79)** | **219724 (180485 , 245478)** | **45.1 (36.6 , 50.4)** | **-35.6 (-47.3 , -25)** |
| **Cambodia** | **24828 (20861 , 29260)** | **217.7 (190.5 , 244.3)** | **12066 (10168 , 13852)** | **115.5 (97.1 , 130.3)** | **-46.9 (-55.2 , -38.5)** |
| **Indonesia** | **91569 (76742 , 112994)** | **58 (49.2 , 66.5)** | **44317 (38124 , 49776)** | **28.4 (24.4 , 31.8)** | **-51 (-58.8 , -41.3)** |
| **Lao People's Democratic Republic** | **9915 (7762 , 12917)** | **192.3 (158.3 , 234.5)** | **3392 (2598 , 4276)** | **76.1 (60.1 , 93)** | **-60.4 (-68.9 , -50.4)** |
| **Malaysia** | **5213 (4654 , 6539)** | **56.5 (49.8 , 73.3)** | **21711 (13543 , 27250)** | **100.7 (62.7 , 125.7)** | **78.1 (-15.7 , 131)** |
| **Maldives** | **42 (31 , 57)** | **29.1 (24.5 , 34.2)** | **35 (28 , 41)** | **13.6 (10.9 , 16.1)** | **-53.1 (-63.3 , -41.3)** |
| **Mauritius** | **320 (300 , 337)** | **51.9 (48 , 54.9)** | **289 (240 , 347)** | **19.8 (16.3 , 23.8)** | **-61.8 (-68.4 , -54.4)** |
| **Myanmar** | **56280 (39028 , 82602)** | **131.2 (100.7 , 174.9)** | **21453 (17187 , 26131)** | **52.1 (42.4 , 62.4)** | **-60.3 (-71.1 , -45.9)** |
| **Philippines** | **42008 (36781 , 48722)** | **94.5 (86.4 , 104.4)** | **58413 (47516 , 66873)** | **92.3 (69.5 , 106.1)** | **-2.3 (-29.4 , 14.1)** |
| **Sri Lanka** | **5212 (4377 , 5761)** | **58.7 (48.8 , 64.9)** | **4699 (3557 , 6197)** | **22.7 (17.3 , 30.2)** | **-61.3 (-71.3 , -44.8)** |
| **Seychelles** | **49 (44 , 55)** | **86.3 (77.2 , 95.9)** | **77 (63 , 88)** | **83.2 (68.8 , 94.2)** | **-3.6 (-18.8 , 10)** |
| **Thailand** | **10745 (8976 , 14893)** | **30.1 (25.2 , 44.4)** | **31037 (19153 , 40632)** | **33.2 (21.1 , 43.2)** | **10.4 (-53.1 , 53)** |
| **Timor-Leste** | **1399 (1099 , 1745)** | **135 (111.2 , 159.9)** | **601 (468 , 737)** | **75 (58.9 , 92.8)** | **-44.4 (-56.6 , -29)** |
| **Viet Nam** | **25878 (21657 , 31090)** | **46.9 (39.3 , 57.3)** | **21345 (17761 , 25715)** | **28.7 (23.9 , 34.8)** | **-38.9 (-49.7 , -24.3)** |
| **Oceania** | **5723 (4819 , 6770)** | **97 (84.4 , 115.2)** | **7771 (5889 , 10105)** | **69.8 (55.7 , 91.7)** | **-28 (-41.4 , -11.9)** |
| **American Samoa** | **10 (8 , 11)** | **39.1 (34 , 44.1)** | **12 (10 , 14)** | **28.9 (24 , 34.1)** | **-25.9 (-39 , -11.4)** |
| **Cook Islands** | **11 (9 , 13)** | **85.4 (74.7 , 97.9)** | **10 (8 , 12)** | **43.4 (35.9 , 52)** | **-49.1 (-59.2 , -37)** |
| **Micronesia (Federated States of)** | **60 (48 , 74)** | **90.9 (74.2 , 111.4)** | **38 (29 , 49)** | **66 (52.3 , 83.1)** | **-27.4 (-44.3 , -7.4)** |
| **Fiji** | **205 (171 , 244)** | **49.8 (42.1 , 58.7)** | **216 (168 , 271)** | **35.1 (28 , 43)** | **-29.5 (-46.7 , -7.6)** |
| **Guam** | **22 (19 , 24)** | **34.9 (30.1 , 39.4)** | **34 (28 , 41)** | **19.1 (15.8 , 22.7)** | **-45.4 (-55.2 , -34.4)** |
| **Kiribati** | **41 (33 , 51)** | **75.5 (62.8 , 89.1)** | **35 (28 , 43)** | **54 (44.4 , 64.4)** | **-28.4 (-42.1 , -10.5)** |
| **Marshall Islands** | **21 (17 , 25)** | **82.2 (68.3 , 100.6)** | **20 (15 , 26)** | **64.2 (50.9 , 81.3)** | **-21.8 (-38.2 , -2.7)** |
| **Nauru** | **6 (5 , 7)** | **91.1 (75.4 , 109.8)** | **4 (3 , 5)** | **71.4 (58.9 , 88.8)** | **-21.7 (-33.6 , -8.2)** |
| **Niue** | **1 (1 , 2)** | **60 (50.1 , 73.2)** | **1 (1 , 1)** | **43.5 (35.3 , 54)** | **-27.6 (-41.6 , -8.6)** |
| **Northern Mariana Islands** | **8 (6 , 10)** | **44.3 (36.2 , 52.5)** | **11 (9 , 12)** | **30.1 (25.4 , 35.1)** | **-32.2 (-44.1 , -18.7)** |
| **Palau** | **16 (13 , 20)** | **163.7 (131.9 , 198.4)** | **18 (14 , 22)** | **119.3 (96.9 , 142.4)** | **-27.1 (-43.8 , -6.7)** |
| **Papua New Guinea** | **4378 (3578 , 5300)** | **100.4 (83.8 , 130.2)** | **6311 (4677 , 8397)** | **71.4 (54.8 , 101.9)** | **-28.9 (-44.9 , -9.6)** |
| **Samoa** | **67 (53 , 86)** | **68.8 (56.8 , 85.7)** | **63 (51 , 81)** | **46.9 (38 , 58.9)** | **-31.9 (-47.9 , -13.2)** |
| **Solomon Islands** | **447 (350 , 547)** | **249.5 (181.2 , 298)** | **479 (392 , 570)** | **151.5 (127.7 , 174.2)** | **-39.3 (-51.1 , -17.4)** |
| **Tokelau** | **1 (1 , 1)** | **66.4 (55.3 , 81.3)** | **0 (0 , 1)** | **39.4 (31.5 , 48.9)** | **-40.7 (-53.6 , -25.3)** |
| **Tonga** | **31 (27 , 36)** | **54.5 (46.6 , 62.5)** | **34 (27 , 41)** | **42.5 (34.3 , 51.6)** | **-21.9 (-37.2 , -2.3)** |
| **Tuvalu** | **10 (8 , 13)** | **113.1 (92.9 , 136.3)** | **5 (4 , 6)** | **53.8 (42.6 , 70.2)** | **-52.4 (-64.2 , -37.1)** |
| **Vanuatu** | **71 (55 , 90)** | **73.9 (56.8 , 94.4)** | **113 (88 , 147)** | **63.1 (48.9 , 81.7)** | **-14.6 (-33.3 , 10.2)** |
| **North Africa and Middle East** | **197703 (168472 , 245401)** | **54.9 (49.2 , 62.5)** | **107742 (94479 , 122048)** | **26.4 (23.2 , 29.6)** | **-51.9 (-59.2 , -45.2)** |
| **Afghanistan** | **23741 (17931 , 32333)** | **145.2 (115.4 , 183.3)** | **18697 (14418 , 23718)** | **61.9 (52.1 , 72.6)** | **-57.3 (-68.4 , -46.3)** |
| **Algeria** | **8125 (6021 , 11240)** | **50.8 (41.1 , 61.5)** | **5786 (4697 , 7112)** | **23.3 (18.9 , 29)** | **-54.2 (-64 , -43.6)** |
| **Bahrain** | **38 (33 , 43)** | **30.2 (26 , 34.5)** | **83 (67 , 101)** | **21.6 (16.4 , 25.6)** | **-28.4 (-44.3 , -11.6)** |
| **Egypt** | **56449 (49213 , 65790)** | **86.9 (78 , 97.8)** | **21371 (16332 , 27730)** | **33.7 (25.8 , 43.9)** | **-61.3 (-70.7 , -48.9)** |
| **Iran (Islamic Republic of)** | **15595 (12752 , 20079)** | **33 (29.4 , 37.7)** | **10219 (9150 , 10998)** | **16.4 (14.5 , 17.8)** | **-50.2 (-57.6 , -43.8)** |
| **Iraq** | **7337 (5768 , 9459)** | **35.1 (29.4 , 41.9)** | **3178 (2605 , 3866)** | **12.8 (10.7 , 15.9)** | **-63.6 (-72.2 , -51.3)** |
| **Jordan** | **780 (643 , 955)** | **33.3 (28.4 , 38.7)** | **1054 (878 , 1275)** | **18.5 (15.5 , 22)** | **-44.3 (-54.4 , -31.5)** |
| **Kuwait** | **191 (175 , 209)** | **29.4 (25.4 , 32.3)** | **668 (551 , 789)** | **35.7 (28.7 , 42.6)** | **21.4 (1.2 , 44.6)** |
| **Lebanon** | **647 (543 , 765)** | **28 (24 , 33)** | **910 (737 , 1293)** | **18.1 (14.7 , 25.7)** | **-35.4 (-49.3 , -6.7)** |
| **Libya** | **840 (660 , 1068)** | **26.9 (21.8 , 32.8)** | **837 (665 , 1038)** | **18.6 (14.8 , 23.1)** | **-30.8 (-46.8 , -10.4)** |
| **Morocco** | **12330 (9986 , 15047)** | **49.5 (41.2 , 58.5)** | **6248 (4884 , 7760)** | **24.8 (19.4 , 30.9)** | **-49.8 (-59.9 , -38.3)** |
| **Palestine** | **487 (384 , 606)** | **38 (30.4 , 51)** | **500 (425 , 646)** | **25.9 (21.7 , 34.1)** | **-31.8 (-45.2 , -14.7)** |
| **Oman** | **438 (345 , 545)** | **57.2 (44.4 , 69.4)** | **407 (344 , 466)** | **40.9 (32.8 , 47.9)** | **-28.5 (-42.8 , -2.7)** |
| **Qatar** | **26 (21 , 34)** | **27 (21.9 , 37.1)** | **70 (54 , 93)** | **24.8 (20 , 31)** | **-8 (-31.7 , 19.3)** |
| **Saudi Arabia** | **2812 (2242 , 3445)** | **43.6 (33.9 , 54.3)** | **4699 (3758 , 5858)** | **32.2 (26.9 , 38.9)** | **-26.1 (-42.5 , 1.9)** |
| **Sudan** | **17665 (10866 , 29396)** | **70.5 (51.8 , 98.4)** | **7026 (5141 , 9198)** | **29.4 (22.1 , 37.2)** | **-58.3 (-72.5 , -43)** |
| **Syrian Arab Republic** | **3592 (2780 , 4713)** | **28.6 (23.8 , 35.3)** | **2284 (1763 , 2908)** | **23.9 (18.9 , 30)** | **-16.5 (-37 , 11)** |
| **Tunisia** | **2348 (1858 , 2975)** | **34.1 (29 , 40.5)** | **1838 (1388 , 2424)** | **17.5 (13.2 , 22.9)** | **-48.7 (-62.2 , -32.3)** |
| **Turkey** | **32062 (24608 , 42458)** | **52.3 (42.9 , 65.5)** | **14868 (11499 , 17816)** | **19 (14.8 , 22.6)** | **-63.6 (-72.3 , -52.7)** |
| **United Arab Emirates** | **177 (144 , 212)** | **83.6 (42 , 100)** | **599 (462 , 760)** | **50.7 (27 , 61.9)** | **-39.3 (-52.4 , -14.4)** |
| **Yemen** | **11890 (7907 , 19469)** | **69.4 (52.1 , 90.9)** | **6289 (4504 , 8359)** | **35.8 (26 , 49.7)** | **-48.4 (-66.1 , -27.5)** |
| **South Asia** | **914842 (809582 , 1025253)** | **85.5 (77 , 94.4)** | **548077 (486877 , 615225)** | **42.4 (37.8 , 47.8)** | **-50.4 (-56.7 , -43.3)** |
| **Bangladesh** | **113930 (97749 , 130771)** | **90.5 (79.8 , 101.5)** | **37302 (30252 , 44245)** | **31.9 (25.8 , 37.7)** | **-64.7 (-71.9 , -56.5)** |
| **Bhutan** | **482 (239 , 744)** | **69.2 (42.3 , 100.3)** | **161 (107 , 224)** | **31.6 (20.6 , 43.8)** | **-54.4 (-70 , -24.9)** |
| **India** | **690913 (595972 , 788320)** | **90.6 (80.5 , 101.1)** | **433661 (381633 , 489555)** | **43.5 (38.3 , 49.3)** | **-52 (-58.8 , -44.5)** |
| **Nepal** | **28603 (23772 , 34001)** | **108.6 (91.6 , 129.3)** | **8760 (6895 , 10669)** | **41.7 (32 , 51.4)** | **-61.6 (-72.2 , -48.9)** |
| **Pakistan** | **80914 (66342 , 95462)** | **55.4 (46.3 , 65.2)** | **68193 (53170 , 85263)** | **37.1 (28.9 , 46.2)** | **-33 (-45.5 , -18.1)** |
| **Southern Sub-Saharan Africa** | **41031 (36908 , 45055)** | **102.6 (92.6 , 112.6)** | **47489 (42559 , 52794)** | **85.4 (77.1 , 93.7)** | **-16.7 (-25.2 , -8.1)** |
| **Botswana** | **868 (662 , 1136)** | **127 (95.5 , 171.3)** | **1528 (1143 , 2040)** | **111.3 (85.1 , 146.1)** | **-12.4 (-34.1 , 16.3)** |
| **Lesotho** | **1736 (1475 , 2035)** | **122.5 (103.9 , 144.1)** | **1941 (1538 , 2388)** | **142.5 (114 , 175)** | **16.3 (-9.2 , 47.1)** |
| **Namibia** | **1218 (936 , 1554)** | **133.5 (103.4 , 173.9)** | **1461 (1090 , 1936)** | **100.4 (76 , 131.7)** | **-24.8 (-41.1 , -2)** |
| **South Africa** | **28202 (25395 , 31156)** | **95.1 (86 , 104.9)** | **28941 (26436 , 31525)** | **69.9 (63.7 , 75.9)** | **-26.5 (-33.2 , -19.2)** |
| **Eswatini** | **691 (542 , 848)** | **120.5 (98.6 , 144.3)** | **721 (537 , 939)** | **105.5 (78.7 , 136.3)** | **-12.4 (-33.4 , 15.2)** |
| **Zimbabwe** | **8317 (6844 , 9773)** | **138.3 (115.1 , 155.8)** | **12897 (10374 , 15611)** | **153.9 (117.4 , 186.7)** | **11.3 (-11 , 38.4)** |
| **Western Sub-Saharan Africa** | **334326 (272594 , 417186)** | **156.7 (135.5 , 181.7)** | **353643 (289548 , 424330)** | **106.3 (91.4 , 122.4)** | **-32.2 (-42.2 , -20.7)** |
| **Benin** | **8732 (6972 , 10640)** | **174.1 (150 , 200.7)** | **9053 (6678 , 12136)** | **111.3 (87.9 , 139.9)** | **-36.1 (-49.9 , -19.7)** |
| **Burkina Faso** | **18692 (14898 , 23409)** | **193.8 (163 , 227.9)** | **24392 (18477 , 30991)** | **138 (115.3 , 162.3)** | **-28.8 (-41.5 , -13.2)** |
| **Cameroon** | **11009 (8590 , 13919)** | **136.5 (109.6 , 164.2)** | **17015 (12717 , 22730)** | **108.2 (82.4 , 141.7)** | **-20.7 (-37.1 , 1)** |
| **Cabo Verde** | **198 (170 , 232)** | **62.2 (54.6 , 70.9)** | **294 (252 , 334)** | **68.9 (59.5 , 78.6)** | **10.6 (-6.6 , 32)** |
| **Chad** | **12639 (9700 , 15936)** | **187.5 (151.6 , 233.3)** | **19447 (15088 , 24999)** | **148.1 (123.8 , 176.4)** | **-21 (-37.5 , -0.9)** |
| **CÃ´te d'Ivoire** | **17023 (13087 , 21205)** | **168.7 (139.9 , 198.9)** | **16980 (12760 , 21673)** | **113.6 (90 , 141.4)** | **-32.6 (-45 , -17.3)** |
| **Gambia** | **1140 (866 , 1443)** | **148.8 (120.1 , 181.3)** | **1223 (981 , 1488)** | **118.2 (93.9 , 142.9)** | **-20.6 (-38.7 , 2.3)** |
| **Ghana** | **12302 (9761 , 15454)** | **132.7 (109 , 161.9)** | **16306 (13384 , 19490)** | **99.6 (84.5 , 115.7)** | **-25 (-42.2 , -5.3)** |
| **Guinea** | **16968 (13553 , 21019)** | **218.5 (182.8 , 258.6)** | **13549 (10224 , 17549)** | **152.8 (120.1 , 191.7)** | **-30.1 (-45.1 , -10.7)** |
| **Guinea-Bissau** | **1656 (1278 , 2145)** | **211.1 (171 , 262.3)** | **1121 (903 , 1394)** | **138.3 (110.8 , 169.8)** | **-34.5 (-49.2 , -17)** |
| **Liberia** | **4269 (3357 , 5241)** | **193.3 (163.4 , 227.4)** | **1940 (1493 , 2476)** | **86.4 (68.8 , 107.5)** | **-55.3 (-64.7 , -43.3)** |
| **Mali** | **10470 (8212 , 13126)** | **103.5 (85.9 , 121.4)** | **15184 (11087 , 20100)** | **71.2 (54.8 , 90.5)** | **-31.2 (-46.8 , -10.4)** |
| **Mauritania** | **2261 (1838 , 2740)** | **141.6 (120.2 , 165.5)** | **1797 (1369 , 2357)** | **82.8 (66 , 103.1)** | **-41.5 (-53.3 , -26.4)** |
| **Niger** | **25919 (19292 , 35069)** | **244.8 (196.9 , 299.1)** | **23642 (17521 , 31295)** | **128.6 (102.9 , 159.8)** | **-47.5 (-59.4 , -31)** |
| **Nigeria** | **169472 (129311 , 224324)** | **151 (121.6 , 187.6)** | **172978 (139565 , 213485)** | **97.4 (82.3 , 115.3)** | **-35.5 (-49.5 , -17.1)** |
| **Sao Tome and Principe** | **147 (122 , 175)** | **146.8 (124.9 , 167.6)** | **109 (88 , 133)** | **111.4 (90.9 , 136.3)** | **-24.2 (-39.2 , -3)** |
| **Senegal** | **8555 (6634 , 10401)** | **124.3 (102.6 , 144.9)** | **6793 (5237 , 8372)** | **77.8 (62.6 , 94.9)** | **-37.4 (-49.9 , -21.6)** |
| **Sierra Leone** | **8971 (6811 , 11166)** | **216.5 (173.3 , 257.9)** | **7460 (5490 , 9758)** | **132.6 (104.4 , 164.3)** | **-38.8 (-52.5 , -21.5)** |
| **Togo** | **3891 (3067 , 4936)** | **148.5 (125.3 , 173.1)** | **4356 (3449 , 5447)** | **111.6 (91.2 , 135.6)** | **-24.9 (-38.6 , -8)** |
| **Eastern Sub-Saharan Africa** | **312749 (262240 , 375937)** | **172.5 (151.6 , 194.4)** | **210094 (180426 , 248234)** | **95.7 (85.2 , 106.9)** | **-44.5 (-51.3 , -37)** |
| **Burundi** | **8583 (6548 , 10701)** | **176.9 (144.1 , 214.6)** | **6462 (4737 , 8693)** | **110 (85.3 , 136.7)** | **-37.8 (-52.2 , -19)** |
| **Comoros** | **696 (424 , 937)** | **150.1 (101.9 , 191.3)** | **450 (355 , 551)** | **91.6 (72.9 , 111.4)** | **-39 (-52 , -10.6)** |
| **Djibouti** | **467 (337 , 626)** | **125 (101.7 , 151.2)** | **593 (451 , 774)** | **93.4 (75.7 , 117.5)** | **-25.2 (-41.2 , -4.7)** |
| **Eritrea** | **5267 (3733 , 7287)** | **208 (144.7 , 318.8)** | **4429 (2899 , 6856)** | **149 (96 , 243.7)** | **-28.3 (-49.6 , 0.6)** |
| **Ethiopia** | **108437 (87669 , 132758)** | **223 (184.7 , 264.3)** | **46301 (39516 , 54642)** | **86.4 (75.4 , 97.7)** | **-61.2 (-68.9 , -51.9)** |
| **Kenya** | **22170 (18737 , 25835)** | **122.9 (106.1 , 144.2)** | **21504 (17976 , 25341)** | **95.9 (80 , 115.7)** | **-22 (-31.6 , -11.1)** |
| **Madagascar** | **18027 (15747 , 20485)** | **151 (135.3 , 167.9)** | **13674 (10800 , 17088)** | **99.1 (78.1 , 122.5)** | **-34.4 (-48 , -18.5)** |
| **Malawi** | **14694 (11759 , 18155)** | **162.6 (138.4 , 190.9)** | **9206 (7575 , 11253)** | **95.1 (80.8 , 110.2)** | **-41.5 (-52 , -28.7)** |
| **Mozambique** | **20901 (14596 , 30440)** | **150.7 (118.7 , 191.1)** | **15524 (12078 , 19370)** | **99.8 (80.7 , 122.4)** | **-33.8 (-49.3 , -12.9)** |
| **Rwanda** | **12357 (9434 , 15601)** | **182 (148.6 , 222.4)** | **5641 (4519 , 6981)** | **85.9 (67.1 , 103)** | **-52.8 (-62.2 , -41.5)** |
| **Somalia** | **14287 (9865 , 19299)** | **204.6 (158.4 , 258.6)** | **20751 (14809 , 28148)** | **144.9 (111.3 , 188.6)** | **-29.2 (-45.6 , -8.3)** |
| **South Sudan** | **10826 (8157 , 14319)** | **171.2 (137.6 , 211)** | **7714 (5824 , 9951)** | **105.8 (85.9 , 129.5)** | **-38.2 (-50.6 , -23.9)** |
| **United Republic of Tanzania** | **45027 (35938 , 55281)** | **172.3 (148 , 198.5)** | **32990 (26686 , 40566)** | **92.1 (78.9 , 107.2)** | **-46.6 (-55.4 , -36)** |
| **Uganda** | **16687 (12114 , 22206)** | **113.1 (89.8 , 139.4)** | **16497 (13088 , 20506)** | **87.2 (71.4 , 102.1)** | **-22.9 (-39.1 , -2.6)** |
| **Zambia** | **14094 (11351 , 17515)** | **175.4 (151 , 201.1)** | **8190 (6645 , 10043)** | **92 (77.7 , 110.4)** | **-47.5 (-58.1 , -34.2)** |
| **Central Sub-Saharan Africa** | **86187 (67712 , 109357)** | **175.6 (144.6 , 214.9)** | **66199 (52715 , 81678)** | **107.7 (85.6 , 139.1)** | **-38.7 (-49.4 , -26.2)** |
| **Angola** | **19616 (12933 , 28945)** | **178.6 (134.6 , 230.4)** | **12783 (10069 , 16121)** | **90.5 (75.9 , 109.3)** | **-49.3 (-63.6 , -27.7)** |
| **Central African Republic** | **4986 (3685 , 6784)** | **202.6 (162.6 , 252.6)** | **5677 (4049 , 7863)** | **161.5 (122.4 , 213.4)** | **-20.3 (-39.3 , 2.5)** |
| **Congo** | **2044 (1551 , 2644)** | **137.3 (107.6 , 169.3)** | **2033 (1588 , 2549)** | **88.8 (71.1 , 108.7)** | **-35.3 (-48 , -19.9)** |
| **Democratic Republic of the Congo** | **57941 (43790 , 73311)** | **177.7 (136.4 , 230.9)** | **44646 (33897 , 57129)** | **111.8 (83.6 , 152.6)** | **-37.1 (-49.6 , -22.6)** |
| **Equatorial Guinea** | **766 (537 , 1124)** | **169.4 (128 , 216.4)** | **363 (261 , 501)** | **78.9 (60.2 , 102.3)** | **-53.4 (-67.1 , -33.6)** |
| **Gabon** | **834 (660 , 1022)** | **116.1 (93 , 138.3)** | **697 (525 , 862)** | **76.3 (59.3 , 93.8)** | **-34.3 (-47.1 , -20.6)** |
