# Supplementary material for: Global burden of lower respiratory infections during the last three decades
Source: Front Public Health. 2023 Jan 9;10:1028525. doi: 10.3389/fpubh.2022.1028525 (PMC9869262; doi:10.3389/fpubh.2022.1028525)
Supplement: Table S4 — DALYs from lower respiratory infections in 1990 and 2019 and the percentage change in the age-standardised rates (ASRs) per 100,000, by location (generated from data available from http://ghdx.healthdata.org/gbd-results-tool). [file Table_4.DOC]

| **Table S4: DALYs from lower respiratory infections in 1990 and 2019 and the percentage change in the age-standardised rates (ASRs) per 100,000, by location**  **(Generated from data available from http://ghdx.healthdata.org/gbd-results-tool)** | | | | | |
| --- | --- | --- | --- | --- | --- |
|  | **1990** | | **2019** | | **Percentage change in ASRs per 100,000** |
|  | **No. (95% UI)** | **ASRs per 100,000 (95% UI)** | **No. (95% UI)** | **ASRs per 100,000 (95% UI)** |
| **Global** | **224389277 (198909049 , 258856776)** | **3700.4 (3302.9 , 4235.2)** | **97189708 (84871207 , 113082891)** | **1386.1 (1203.5 , 1622.8)** | **-62.5 (-69 , -54.9)** |
| **High-income North America** | **1339748 (1268188 , 1381334)** | **404.5 (386 , 416)** | **1339935 (1240713 , 1397495)** | **239.7 (225.9 , 248.3)** | **-40.7 (-42.3 , -39.1)** |
| **Canada** | **99801 (93439 , 104468)** | **333.3 (311.6 , 348.7)** | **110971 (98261 , 121348)** | **171.6 (154.3 , 185.5)** | **-48.5 (-52.7 , -44.6)** |
| **Greenland** | **458 (382 , 556)** | **1115.5 (933 , 1306.4)** | **248 (196 , 304)** | **432 (342.1 , 531.9)** | **-61.3 (-70.7 , -50.5)** |
| **United States of America** | **1239459 (1173924 , 1278061)** | **412.3 (393.8 , 424.2)** | **1228694 (1142113 , 1282287)** | **247.6 (233.8 , 256.1)** | **-40 (-41.6 , -38.3)** |
| **Australasia** | **49244 (46377 , 51407)** | **242.1 (227.3 , 253.5)** | **59638 (52027 , 65001)** | **127.6 (113.6 , 138.1)** | **-47.3 (-51.7 , -43.3)** |
| **Australia** | **33548 (31686 , 35223)** | **196.7 (185.2 , 207.5)** | **49955 (43426 , 54965)** | **126.7 (112.7 , 137.8)** | **-35.6 (-41.6 , -29.6)** |
| **New Zealand** | **15695 (14575 , 16662)** | **454.2 (421.1 , 482.3)** | **9683 (8454 , 10753)** | **132 (116.5 , 145.4)** | **-70.9 (-73.7 , -68)** |
| **High-income Asia Pacific** | **1122131 (1058846 , 1155657)** | **662.3 (622.1 , 686.5)** | **1499688 (1268092 , 1638181)** | **305.3 (267.8 , 327.1)** | **-53.9 (-57.4 , -51.6)** |
| **Brunei Darussalam** | **1218 (1066 , 1415)** | **846.2 (739.4 , 1053.8)** | **2128 (1737 , 2484)** | **861.9 (725.6 , 995.6)** | **1.9 (-29.4 , 24.2)** |
| **Japan** | **942365 (886697 , 969941)** | **638.4 (599.6 , 658.1)** | **1248382 (1032784 , 1368420)** | **313.6 (274.5 , 335.7)** | **-50.9 (-54.2 , -48.5)** |
| **Singapore** | **27993 (26731 , 29407)** | **1348.9 (1270.4 , 1425.2)** | **48695 (42552 , 53019)** | **701.7 (611.9 , 766.1)** | **-48 (-52.6 , -43.4)** |
| **Republic of Korea** | **150556 (139406 , 164376)** | **501.2 (464.3 , 550.4)** | **200483 (118361 , 226965)** | **251.7 (156 , 283.8)** | **-49.8 (-71.3 , -42.4)** |
| **Western Europe** | **1803816 (1705225 , 1858531)** | **353.1 (334.9 , 364.5)** | **1802175 (1609136 , 1909902)** | **192.7 (176 , 202.7)** | **-45.4 (-47.9 , -43.4)** |
| **Andorra** | **118 (89 , 158)** | **283.4 (219.1 , 364.5)** | **236 (183 , 295)** | **171 (132.8 , 214.6)** | **-39.7 (-57.2 , -17.5)** |
| **Austria** | **23012 (21745 , 24180)** | **237 (222.8 , 251.4)** | **14968 (13299 , 16357)** | **88.5 (80.4 , 96)** | **-62.6 (-65.9 , -59)** |
| **Belgium** | **49628 (46457 , 52185)** | **349.4 (327.6 , 365.9)** | **70300 (60988 , 76933)** | **283.6 (252.5 , 308.1)** | **-18.8 (-25.2 , -11.8)** |
| **Cyprus** | **2705 (2237 , 3101)** | **426.7 (352.2 , 489.3)** | **2350 (2040 , 2752)** | **145.4 (126.7 , 170.2)** | **-65.9 (-72.5 , -56.5)** |
| **Denmark** | **24808 (23209 , 26153)** | **328 (308.6 , 347)** | **27156 (23733 , 29944)** | **229.5 (203.2 , 251.1)** | **-30 (-36.8 , -23)** |
| **Finland** | **37374 (34958 , 39292)** | **559.5 (522 , 586.3)** | **9408 (8363 , 10387)** | **79.1 (71.7 , 86.5)** | **-85.9 (-87.1 , -84.6)** |
| **France** | **238887 (221673 , 251609)** | **300 (280.3 , 314.3)** | **234856 (198363 , 261674)** | **153.2 (134.9 , 168.6)** | **-48.9 (-53.3 , -44.7)** |
| **Germany** | **299424 (281596 , 312822)** | **262.8 (248.7 , 275.3)** | **317690 (286739 , 346479)** | **169.2 (154.5 , 182.8)** | **-35.6 (-40.6 , -30.2)** |
| **Greece** | **31510 (29629 , 33794)** | **251.3 (232.3 , 282)** | **68121 (59822 , 74633)** | **281.2 (253.5 , 306.9)** | **11.9 (-1.6 , 24)** |
| **Iceland** | **1399 (1278 , 1499)** | **484.2 (444.5 , 519.3)** | **1153 (972 , 1315)** | **203.5 (174.7 , 233.2)** | **-58 (-63.1 , -52.1)** |
| **Ireland** | **27163 (25727 , 28332)** | **708.2 (665.4 , 738.6)** | **18916 (16421 , 20913)** | **253.9 (220.8 , 280.3)** | **-64.2 (-67.7 , -60.6)** |
| **Israel** | **14580 (13635 , 15690)** | **308 (287.9 , 330.7)** | **22912 (20492 , 25049)** | **194.5 (175.9 , 211.8)** | **-36.9 (-43 , -30.4)** |
| **Italy** | **146898 (140738 , 152003)** | **231.9 (220.6 , 246.6)** | **139312 (121142 , 148959)** | **98.6 (89.5 , 104.1)** | **-57.5 (-60.7 , -55)** |
| **Luxembourg** | **1428 (1323 , 1529)** | **304.7 (283.2 , 325.8)** | **1622 (1357 , 1870)** | **160.6 (135.4 , 185.3)** | **-47.3 (-55.3 , -38.4)** |
| **Malta** | **1657 (1533 , 1771)** | **437.2 (404.3 , 469.7)** | **2317 (1983 , 2663)** | **275.8 (237.9 , 319.6)** | **-36.9 (-46.1 , -26.4)** |
| **Monaco** | **195 (154 , 241)** | **331.8 (270.1 , 400.4)** | **266 (215 , 314)** | **272.8 (218.9 , 323.7)** | **-17.8 (-35.9 , 2.6)** |
| **Netherlands** | **51233 (46862 , 54249)** | **271.4 (249.6 , 286.9)** | **69853 (61244 , 77348)** | **202.6 (180 , 222.8)** | **-25.3 (-31.9 , -18.7)** |
| **Norway** | **35626 (32710 , 37201)** | **503.3 (464.2 , 524.9)** | **20003 (17307 , 21650)** | **184.9 (163.4 , 198.8)** | **-63.3 (-65.2 , -61)** |
| **Portugal** | **66963 (63790 , 70489)** | **641.8 (604.9 , 689.8)** | **95138 (82957 , 103759)** | **387.3 (349 , 418.6)** | **-39.7 (-46.5 , -33.7)** |
| **San Marino** | **55 (47 , 65)** | **197.8 (168.1 , 232.3)** | **87 (61 , 117)** | **131.1 (91.3 , 179.2)** | **-33.7 (-54.9 , -6.3)** |
| **Spain** | **150705 (141090 , 157956)** | **338.3 (317.9 , 358)** | **157776 (137114 , 174633)** | **158.2 (140.4 , 171.9)** | **-53.2 (-57.4 , -49.2)** |
| **Sweden** | **56172 (51766 , 59717)** | **377.9 (351.8 , 398.5)** | **30929 (27036 , 34024)** | **140.3 (125.4 , 152.8)** | **-62.9 (-65.8 , -59.9)** |
| **Switzerland** | **35097 (32487 , 37086)** | **367.5 (340.9 , 390.1)** | **20510 (17408 , 22810)** | **111.2 (97.4 , 122.3)** | **-69.7 (-72.5 , -66.7)** |
| **United Kingdom** | **505679 (474990 , 523027)** | **606 (570.3 , 626.6)** | **474725 (427844 , 500657)** | **373.3 (342.6 , 390.4)** | **-38.4 (-40.7 , -35.6)** |
| **Southern Latin America** | **466684 (447088 , 486683)** | **990.1 (949.1 , 1031.4)** | **692430 (638457 , 742965)** | **883.9 (816.9 , 950.3)** | **-10.7 (-17.4 , -3.3)** |
| **Argentina** | **263469 (249155 , 279155)** | **819.5 (776.2 , 867.7)** | **601861 (552608 , 647818)** | **1166.8 (1076.4 , 1257.1)** | **42.4 (30.4 , 56.3)** |
| **Chile** | **182957 (173961 , 192352)** | **1631.1 (1559.6 , 1705.7)** | **67800 (60785 , 73679)** | **317.3 (281.4 , 348)** | **-80.5 (-82.6 , -78.5)** |
| **Uruguay** | **20238 (18740 , 21608)** | **628.1 (577.5 , 674.1)** | **22734 (20264 , 24870)** | **468.3 (417.2 , 523)** | **-25.4 (-34.4 , -14.8)** |
| **Eastern Europe** | **1428212 (1377780 , 1496943)** | **769 (739.5 , 814.2)** | **1423084 (1271089 , 1588785)** | **581.2 (519.6 , 643.4)** | **-24.4 (-33.1 , -15.7)** |
| **Belarus** | **54642 (49366 , 60542)** | **632.6 (562.5 , 713.6)** | **30832 (24172 , 39001)** | **263.1 (206.1 , 332.9)** | **-58.4 (-67.8 , -45.9)** |
| **Estonia** | **7916 (7373 , 8506)** | **534.8 (491.9 , 582.1)** | **4934 (3950 , 6058)** | **276.2 (223.2 , 338.6)** | **-48.3 (-59 , -34.9)** |
| **Latvia** | **13225 (12527 , 13948)** | **507.6 (476 , 540.5)** | **8536 (7146 , 10245)** | **335.7 (277 , 404.6)** | **-33.9 (-45.5 , -19.9)** |
| **Lithuania** | **11815 (10949 , 12730)** | **335.7 (308.2 , 367.3)** | **12352 (10192 , 14728)** | **327.9 (275.3 , 388)** | **-2.3 (-20.4 , 19.2)** |
| **Republic of Moldova** | **74907 (66929 , 84471)** | **1856.4 (1652.8 , 2104.2)** | **30056 (26288 , 33982)** | **855 (735.6 , 987.8)** | **-53.9 (-62.1 , -43.5)** |
| **Russian Federation** | **1045025 (1006545 , 1098347)** | **855.4 (821.6 , 907.5)** | **1038284 (898756 , 1184244)** | **603.5 (527.2 , 684.2)** | **-29.5 (-38.8 , -19.7)** |
| **Ukraine** | **220683 (204600 , 239201)** | **481 (436.3 , 533)** | **298091 (247273 , 361627)** | **587.5 (493.4 , 700.1)** | **22.1 (-0.5 , 50.8)** |
| **Central Europe** | **1265433 (1211816 , 1332512)** | **1243 (1181.1 , 1322.5)** | **599841 (528609 , 667838)** | **403.1 (354.3 , 453.5)** | **-67.6 (-72.2 , -62.9)** |
| **Albania** | **143331 (115628 , 165425)** | **3810.4 (2966.5 , 4357.3)** | **12405 (10106 , 15174)** | **537.3 (434.7 , 655.3)** | **-85.9 (-88.9 , -80.2)** |
| **Bosnia and Herzegovina** | **14191 (12868 , 15485)** | **383.3 (343.7 , 418.8)** | **7386 (5825 , 9717)** | **164.8 (132.7 , 212)** | **-57 (-66.3 , -43.6)** |
| **Bulgaria** | **133589 (126952 , 142347)** | **1756.3 (1660 , 1922.7)** | **45372 (36427 , 56327)** | **551 (441 , 681.6)** | **-68.6 (-75.3 , -60.5)** |
| **Croatia** | **19228 (18017 , 20382)** | **380.4 (355.9 , 406.3)** | **8779 (7093 , 10790)** | **117.9 (94.1 , 145.6)** | **-69 (-75.4 , -61.9)** |
| **Czechia** | **49889 (47754 , 52072)** | **452 (430.6 , 476.9)** | **53159 (43629 , 64083)** | **294.8 (241.3 , 356.4)** | **-34.8 (-46.3 , -20.6)** |
| **Hungary** | **40544 (38461 , 42689)** | **444.3 (413.8 , 476)** | **22092 (18260 , 26725)** | **161.3 (131.9 , 197)** | **-63.7 (-70.2 , -55.3)** |
| **Montenegro** | **2325 (1994 , 2722)** | **426.7 (361 , 504.7)** | **1417 (1181 , 1704)** | **189.8 (157.8 , 227.5)** | **-55.5 (-66 , -43.8)** |
| **North Macedonia** | **21623 (17909 , 26067)** | **1246.7 (1033.8 , 1506.3)** | **4399 (3484 , 5577)** | **221.5 (176.4 , 272.5)** | **-82.2 (-87 , -75.3)** |
| **Poland** | **196201 (188195 , 208158)** | **558.9 (532.5 , 602)** | **206880 (175318 , 242716)** | **352 (298.8 , 410.3)** | **-37 (-47.6 , -26.2)** |
| **Romania** | **515015 (489479 , 562751)** | **2871.1 (2709.2 , 3171.5)** | **167694 (142382 , 196662)** | **843.5 (728.2 , 977.5)** | **-70.6 (-75.4 , -65.3)** |
| **Serbia** | **51450 (42503 , 66462)** | **677.8 (540 , 917.2)** | **28683 (22268 , 35165)** | **225.5 (179 , 275.6)** | **-66.7 (-77.6 , -54)** |
| **Slovakia** | **68678 (56078 , 73702)** | **1322.4 (1108.5 , 1418)** | **33547 (26194 , 41615)** | **456.9 (358.1 , 567.1)** | **-65.5 (-73.5 , -54.6)** |
| **Slovenia** | **9371 (7563 , 11478)** | **445.4 (367.6 , 533.1)** | **8029 (6342 , 10123)** | **178.8 (143 , 224)** | **-59.8 (-69.6 , -47.4)** |
| **Central Asia** | **4556881 (4152040 , 5023048)** | **4988.5 (4558.5 , 5482.3)** | **1507867 (1262441 , 1821204)** | **1668.9 (1405 , 2008.9)** | **-66.5 (-72.5 , -59.1)** |
| **Armenia** | **89821 (78654 , 101637)** | **2450.5 (2152.8 , 2767)** | **17490 (14693 , 20776)** | **692.7 (566.5 , 851.8)** | **-71.7 (-77.6 , -63.6)** |
| **Azerbaijan** | **728872 (626549 , 846948)** | **8148.9 (7016.4 , 9461.1)** | **153765 (116962 , 198846)** | **2058.3 (1555.3 , 2673.9)** | **-74.7 (-81.3 , -66)** |
| **Georgia** | **134606 (118168 , 153035)** | **3060.5 (2674.9 , 3497)** | **18401 (15887 , 21208)** | **439.7 (374.8 , 516.3)** | **-85.6 (-88.1 , -82.5)** |
| **Kazakhstan** | **469553 (421235 , 524531)** | **2708.9 (2438 , 3014.8)** | **151364 (129610 , 175932)** | **840.8 (722 , 973.1)** | **-69 (-73.9 , -62.9)** |
| **Kyrgyzstan** | **271281 (241503 , 305302)** | **4563.3 (4076.7 , 5124.5)** | **48753 (40599 , 55948)** | **716.5 (602.6 , 818.5)** | **-84.3 (-87 , -81.2)** |
| **Mongolia** | **258482 (214777 , 302529)** | **7925 (6567.4 , 9182.4)** | **34750 (25838 , 45596)** | **959.8 (717.2 , 1247.9)** | **-87.9 (-91.1 , -82.8)** |
| **Tajikistan** | **653498 (556530 , 748372)** | **7349.4 (6327.9 , 8342.9)** | **224864 (168308 , 299533)** | **2159.8 (1683 , 2832.3)** | **-70.6 (-78.1 , -59.8)** |
| **Turkmenistan** | **417106 (366800 , 473601)** | **7133.3 (6288.4 , 8073.6)** | **94557 (73551 , 123575)** | **1768.2 (1385.9 , 2296.8)** | **-75.2 (-81.6 , -66.6)** |
| **Uzbekistan** | **1533661 (1353578 , 1725544)** | **4712.4 (4182.8 , 5278.1)** | **763923 (634184 , 938076)** | **2291.9 (1914.4 , 2796.2)** | **-51.4 (-61 , -39.3)** |
| **Central Latin America** | **3809976 (3450707 , 4124062)** | **1990.7 (1833.1 , 2128.2)** | **1642711 (1352551 , 2011184)** | **717.4 (586.1 , 882)** | **-64 (-71 , -55.1)** |
| **Colombia** | **471218 (411887 , 535521)** | **1306 (1169.9 , 1451.1)** | **225558 (168552 , 296859)** | **493.6 (363 , 656.8)** | **-62.2 (-73.5 , -48.6)** |
| **Costa Rica** | **18502 (15894 , 22294)** | **619.9 (551.7 , 715)** | **14697 (11425 , 18689)** | **313.5 (240.7 , 402.3)** | **-49.4 (-62 , -33.2)** |
| **El Salvador** | **105149 (88580 , 125928)** | **1723.2 (1503.5 , 1994.3)** | **50577 (37692 , 66515)** | **838.9 (623.7 , 1106.8)** | **-51.3 (-66.1 , -33.6)** |
| **Guatemala** | **674079 (601494 , 747457)** | **7040.2 (6397.3 , 7715)** | **388779 (295765 , 511035)** | **2470.2 (1916.8 , 3182.5)** | **-64.9 (-73.2 , -53.9)** |
| **Honduras** | **110939 (91120 , 136400)** | **1641.5 (1383.5 , 1947.9)** | **40990 (28685 , 58475)** | **503.9 (385.9 , 706.3)** | **-69.3 (-76.7 , -54.5)** |
| **Mexico** | **2042052 (1759877 , 2293247)** | **2041.6 (1800.1 , 2257.5)** | **686548 (598217 , 790003)** | **608.8 (528.5 , 707.3)** | **-70.2 (-75.1 , -63.9)** |
| **Nicaragua** | **153442 (124037 , 185231)** | **2501.3 (2085.9 , 2971.3)** | **45170 (36753 , 54666)** | **794.7 (657.5 , 953.9)** | **-68.2 (-75.8 , -58.2)** |
| **Panama** | **17169 (14681 , 20431)** | **733.6 (641.3 , 851.5)** | **25181 (18943 , 32557)** | **632.1 (472.7 , 821.8)** | **-13.8 (-36.2 , 13.9)** |
| **Venezuela (Bolivarian Republic of)** | **217425 (199478 , 237707)** | **1107 (1034.2 , 1186.8)** | **165212 (123261 , 214866)** | **630.8 (468 , 826.4)** | **-43 (-58 , -24.9)** |
| **Andean Latin America** | **2280916 (2011698 , 2582056)** | **4804.4 (4320.8 , 5356.3)** | **784845 (624452 , 977491)** | **1326.9 (1060.4 , 1654)** | **-72.4 (-78.9 , -65)** |
| **Bolivia (Plurinational State of)** | **751205 (600678 , 922955)** | **7971.6 (6618.6 , 9633.2)** | **241541 (189569 , 304624)** | **2083.4 (1668.1 , 2539.7)** | **-73.9 (-80.6 , -66)** |
| **Ecuador** | **254446 (222723 , 286204)** | **2273.6 (2044.2 , 2506.2)** | **150505 (119477 , 187949)** | **961.5 (767.9 , 1196.3)** | **-57.7 (-66.5 , -46.2)** |
| **Peru** | **1275265 (1093405 , 1479350)** | **4869.1 (4269.2 , 5550.9)** | **392799 (289510 , 522859)** | **1212.2 (893.1 , 1616.5)** | **-75.1 (-82.4 , -65.6)** |
| **Caribbean** | **1000011 (858471 , 1171319)** | **2587.1 (2244.7 , 2987.2)** | **621803 (508359 , 753074)** | **1418.8 (1149.9 , 1728.7)** | **-45.2 (-57.9 , -29.6)** |
| **Antigua and Barbuda** | **465 (419 , 512)** | **789.6 (716.5 , 866.2)** | **578 (486 , 689)** | **712.6 (590.3 , 864.3)** | **-9.8 (-26.2 , 9.9)** |
| **Barbados** | **1859 (1709 , 2027)** | **710.1 (648.9 , 779.3)** | **2564 (2082 , 3041)** | **657.8 (527.8 , 800.9)** | **-7.4 (-27.3 , 14.3)** |
| **Belize** | **3561 (3050 , 4133)** | **1700.8 (1505.1 , 1908.6)** | **3285 (2800 , 3808)** | **1021.5 (878.9 , 1178.4)** | **-39.9 (-50.5 , -27.6)** |
| **Bermuda** | **253 (232 , 274)** | **456.9 (416.8 , 497)** | **241 (202 , 293)** | **224.1 (186.9 , 274.8)** | **-51 (-59.7 , -39.9)** |
| **Bahamas** | **2285 (2030 , 2582)** | **1129.5 (1014.1 , 1256.9)** | **2293 (1859 , 2785)** | **691.7 (561.4 , 834.1)** | **-38.8 (-50.8 , -23.4)** |
| **Cuba** | **72622 (69042 , 76155)** | **739.4 (700.5 , 775)** | **103766 (85129 , 124364)** | **606.2 (499.7 , 724.2)** | **-18 (-32.8 , -3.3)** |
| **Dominica** | **660 (581 , 752)** | **872.4 (771.8 , 994.4)** | **604 (481 , 747)** | **885.4 (681.9 , 1129.4)** | **1.5 (-22.6 , 32.4)** |
| **Dominican Republic** | **156661 (126331 , 190436)** | **1627 (1353.6 , 1939.3)** | **63642 (47655 , 83188)** | **628.2 (477 , 811.8)** | **-61.4 (-72.1 , -48.1)** |
| **Grenada** | **1465 (1255 , 1688)** | **1715.5 (1484.2 , 1965.8)** | **1064 (929 , 1203)** | **1136.2 (983.9 , 1311.9)** | **-33.8 (-44.9 , -20.2)** |
| **Guyana** | **12545 (10713 , 14495)** | **1839.5 (1605.2 , 2075.2)** | **8097 (6007 , 10540)** | **1252.3 (951.4 , 1599.1)** | **-31.9 (-49.5 , -10.9)** |
| **Haiti** | **648923 (522126 , 808659)** | **6912.6 (5706.3 , 8270.1)** | **372275 (285326 , 470427)** | **2919.6 (2249 , 3679.2)** | **-57.8 (-69.2 , -43.2)** |
| **Jamaica** | **16818 (14805 , 19395)** | **710.7 (633 , 804.3)** | **9417 (7401 , 11860)** | **347.7 (265.2 , 445.3)** | **-51.1 (-62.4 , -37.4)** |
| **Puerto Rico** | **29166 (27779 , 30603)** | **839.4 (799.4 , 881.2)** | **19576 (15341 , 24568)** | **353.2 (277.6 , 446.7)** | **-57.9 (-67.1 , -46.6)** |
| **Saint Kitts and Nevis** | **586 (529 , 645)** | **1518.4 (1380.8 , 1663.1)** | **452 (358 , 553)** | **849.9 (682.8 , 1024.4)** | **-44 (-54.9 , -31.1)** |
| **Saint Lucia** | **1146 (1013 , 1297)** | **963.8 (877.9 , 1059.3)** | **1049 (867 , 1256)** | **604.5 (490.2 , 740)** | **-37.3 (-49.8 , -22.7)** |
| **Saint Vincent and the Grenadines** | **1045 (891 , 1227)** | **1062.2 (935.2 , 1206.7)** | **825 (701 , 979)** | **737.2 (617 , 888.1)** | **-30.6 (-43.9 , -13.4)** |
| **Suriname** | **4625 (3769 , 5423)** | **1263.1 (1058.7 , 1449.7)** | **4282 (3446 , 5289)** | **813.8 (649.4 , 1018.5)** | **-35.6 (-50.5 , -15.4)** |
| **Trinidad and Tobago** | **11606 (10643 , 12708)** | **1154.7 (1071 , 1248.2)** | **6270 (4712 , 8141)** | **452.4 (336.1 , 596.4)** | **-60.8 (-71 , -47.6)** |
| **United States Virgin Islands** | **408 (339 , 481)** | **452.2 (380 , 526.6)** | **458 (386 , 533)** | **301.2 (251.6 , 353.3)** | **-33.4 (-46.2 , -16.4)** |
| **Tropical Latin America** | **4102114 (3657480 , 4790880)** | **2718.7 (2454.4 , 3119)** | **2033803 (1884705 , 2176918)** | **956.6 (876.5 , 1039.6)** | **-64.8 (-70.6 , -59.8)** |
| **Brazil** | **4040744 (3595360 , 4725563)** | **2769.8 (2494.9 , 3185.9)** | **1994790 (1851233 , 2127571)** | **965.1 (886 , 1047.2)** | **-65.2 (-70.8 , -60.2)** |
| **Paraguay** | **61370 (51782 , 71668)** | **1246 (1083.1 , 1422)** | **39013 (29110 , 51178)** | **658.5 (492.5 , 862.1)** | **-47.2 (-62.3 , -29.9)** |
| **East Asia** | **39317540 (33666908 , 45248978)** | **3408.4 (2930.6 , 3899.4)** | **4372837 (3904840 , 4951861)** | **355.3 (316.7 , 404.3)** | **-89.6 (-91.4 , -86.8)** |
| **China** | **38278504 (32813977 , 43962340)** | **3454 (2967.5 , 3941.7)** | **4020676 (3555790 , 4589775)** | **343.1 (304.6 , 391.6)** | **-90.1 (-91.8 , -87.3)** |
| **Democratic People's Republic of Korea** | **943770 (679102 , 1350316)** | **3191.2 (2371.6 , 4431.7)** | **171289 (137173 , 210660)** | **722.8 (574.2 , 898.4)** | **-77.4 (-84.4 , -68.7)** |
| **Taiwan (Province of China)** | **95266 (91018 , 99323)** | **640 (608.5 , 668.2)** | **180872 (144242 , 229563)** | **500 (399.5 , 634.4)** | **-21.9 (-37 , -2.1)** |
| **Southeast Asia** | **19270947 (15879164 , 24528371)** | **3602.2 (3038.9 , 4463.8)** | **6925100 (6023294 , 7827538)** | **1246.9 (1080.2 , 1414)** | **-65.4 (-72.5 , -57.3)** |
| **Cambodia** | **1932247 (1590319 , 2318512)** | **11439.2 (9669.5 , 13426.5)** | **496631 (406720 , 610042)** | **3385.7 (2827.5 , 4037)** | **-70.4 (-76.2 , -63.4)** |
| **Indonesia** | **6778510 (5551947 , 8797469)** | **3261.4 (2706.4 , 4118.9)** | **1570882 (1337040 , 1851398)** | **821.6 (698.9 , 967.2)** | **-74.8 (-81 , -68.1)** |
| **Lao People's Democratic Republic** | **795054 (609990 , 1056725)** | **11916.3 (9347 , 15513.8)** | **180605 (130654 , 240781)** | **2665.9 (1978.1 , 3467.6)** | **-77.6 (-83.7 , -70.1)** |
| **Malaysia** | **195111 (168292 , 231384)** | **1412.8 (1261.1 , 1732)** | **463891 (305537 , 581980)** | **1808 (1180.8 , 2259.8)** | **28 (-32.6 , 64.4)** |
| **Maldives** | **2801 (1961 , 4072)** | **1012.2 (791.5 , 1341.5)** | **984 (810 , 1203)** | **288 (235.6 , 345.3)** | **-71.5 (-78.8 , -62)** |
| **Mauritius** | **9948 (9353 , 10569)** | **1185.2 (1117.4 , 1251.2)** | **6077 (4981 , 7449)** | **457.2 (371.6 , 564)** | **-61.4 (-69 , -52.4)** |
| **Myanmar** | **4293106 (2793195 , 6588153)** | **7788.7 (5289.8 , 11645.2)** | **930003 (683450 , 1237535)** | **1919.8 (1430.7 , 2528.9)** | **-75.4 (-84.2 , -61)** |
| **Philippines** | **2793036 (2361509 , 3378380)** | **3675.2 (3217.1 , 4284.1)** | **1943794 (1667975 , 2229636)** | **2102.5 (1802.9 , 2388.2)** | **-42.8 (-53.7 , -31.4)** |
| **Sri Lanka** | **178563 (151789 , 203601)** | **1351 (1151.9 , 1513.1)** | **109053 (82530 , 143567)** | **493.1 (376.1 , 643.2)** | **-63.5 (-73.3 , -51.1)** |
| **Seychelles** | **1231 (1108 , 1348)** | **1982.9 (1785 , 2176.2)** | **1756 (1462 , 2011)** | **1743.6 (1468.9 , 1984.6)** | **-12.1 (-25.2 , 1.4)** |
| **Thailand** | **554821 (445252 , 718960)** | **1162.2 (940 , 1504.3)** | **603773 (387121 , 790271)** | **724.6 (511.7 , 929.5)** | **-37.7 (-64.2 , -12.9)** |
| **Timor-Leste** | **116067 (90349 , 146190)** | **8220.4 (6499.7 , 10117.1)** | **28203 (19597 , 36042)** | **2131.1 (1582.1 , 2645.1)** | **-74.1 (-81.3 , -65)** |
| **Viet Nam** | **1594836 (1298561 , 1988278)** | **2072.8 (1709.3 , 2522.4)** | **580374 (482935 , 707619)** | **742.6 (618.5 , 911.2)** | **-64.2 (-72.2 , -53.6)** |
| **Oceania** | **426916 (346321 , 513410)** | **4947 (4170.5 , 5844.2)** | **546681 (406465 , 721967)** | **3353.4 (2528.9 , 4353)** | **-32.2 (-48.4 , -11.6)** |
| **American Samoa** | **493 (403 , 600)** | **1093.2 (946.7 , 1267.3)** | **371 (293 , 466)** | **758.6 (602.1 , 943.5)** | **-30.6 (-45.4 , -11.7)** |
| **Cook Islands** | **457 (378 , 562)** | **2586.8 (2187 , 3121.2)** | **198 (159 , 243)** | **924 (719.1 , 1143.3)** | **-64.3 (-73.8 , -53)** |
| **Micronesia (Federated States of)** | **3633 (2817 , 4558)** | **3314.3 (2636.6 , 4069)** | **1477 (1037 , 1898)** | **1782.4 (1324.9 , 2232.6)** | **-46.2 (-60.6 , -30.3)** |
| **Fiji** | **10840 (8761 , 13220)** | **1620.9 (1347.4 , 1936.8)** | **9340 (7008 , 12018)** | **1148.1 (874.7 , 1454.3)** | **-29.2 (-49.5 , -1.6)** |
| **Guam** | **974 (845 , 1110)** | **860.6 (761.3 , 960.7)** | **1083 (869 , 1309)** | **632.8 (506.2 , 766)** | **-26.5 (-41.5 , -9.3)** |
| **Kiribati** | **2607 (2001 , 3372)** | **3078.1 (2466.4 , 3791.2)** | **1651 (1305 , 2062)** | **1616.2 (1301.5 , 2001.1)** | **-47.5 (-60.5 , -31.8)** |
| **Marshall Islands** | **1249 (1026 , 1502)** | **2789.4 (2339.7 , 3322.1)** | **931 (703 , 1194)** | **1942 (1501.7 , 2475.3)** | **-30.4 (-47 , -11.2)** |
| **Nauru** | **381 (297 , 495)** | **3254.5 (2611.6 , 4080.2)** | **221 (171 , 294)** | **2321.1 (1865.1 , 3005.1)** | **-28.7 (-40.2 , -14.7)** |
| **Niue** | **48 (37 , 60)** | **1955 (1538.8 , 2446.6)** | **22 (18 , 28)** | **1333.8 (1028.5 , 1724.9)** | **-31.8 (-48.1 , -11.4)** |
| **Northern Mariana Islands** | **395 (304 , 489)** | **1147 (934.2 , 1378.4)** | **307 (255 , 368)** | **765.2 (637.1 , 918.2)** | **-33.3 (-45.1 , -19.5)** |
| **Palau** | **740 (564 , 936)** | **5318.5 (4132.4 , 6657)** | **543 (429 , 675)** | **3237.4 (2607.4 , 3949.4)** | **-39.1 (-53.1 , -22.1)** |
| **Papua New Guinea** | **347025 (277264 , 424542)** | **5778 (4712 , 7035.1)** | **474045 (344527 , 630539)** | **3625.4 (2691.7 , 4821.5)** | **-37.3 (-54.1 , -15.9)** |
| **Samoa** | **3291 (2440 , 4365)** | **2327.3 (1781.8 , 3030.5)** | **1986 (1471 , 2611)** | **1183 (898.8 , 1537.1)** | **-49.2 (-64.8 , -29)** |
| **Solomon Islands** | **24851 (19332 , 31813)** | **7915.2 (6102.6 , 9651.4)** | **22168 (17681 , 26851)** | **4239.4 (3490.7 , 5013.9)** | **-46.4 (-57.7 , -28.8)** |
| **Tokelau** | **45 (35 , 57)** | **2212.2 (1761.2 , 2761.4)** | **13 (10 , 17)** | **964.3 (739.6 , 1252.2)** | **-56.4 (-67.9 , -41.7)** |
| **Tonga** | **1357 (1139 , 1621)** | **1558.8 (1341.7 , 1800)** | **955 (737 , 1228)** | **1040.5 (814.1 , 1317.9)** | **-33.2 (-49.1 , -13.6)** |
| **Tuvalu** | **627 (482 , 865)** | **5405 (4281.1 , 7181.7)** | **154 (117 , 209)** | **1470.9 (1118.2 , 1991.5)** | **-72.8 (-81.5 , -61.9)** |
| **Vanuatu** | **4278 (3251 , 5487)** | **2554.9 (1981.6 , 3242.7)** | **5398 (4057 , 7010)** | **2006.5 (1541.1 , 2586.8)** | **-21.5 (-40.3 , 4.1)** |
| **North Africa and Middle East** | **15176430 (12652761 , 19535942)** | **3101.8 (2636.4 , 3870.7)** | **4716300 (3993317 , 5473257)** | **888.5 (761.1 , 1019.9)** | **-71.4 (-77.8 , -65.1)** |
| **Afghanistan** | **1898139 (1407505 , 2647037)** | **9261.1 (6995.7 , 12599.5)** | **1402790 (1028351 , 1838486)** | **2642.7 (2076.3 , 3280.2)** | **-71.5 (-80 , -60.7)** |
| **Algeria** | **555621 (369541 , 827957)** | **1897 (1393.6 , 2644.1)** | **177301 (145455 , 217772)** | **505.6 (417.7 , 618.5)** | **-73.3 (-82 , -62.8)** |
| **Bahrain** | **1662 (1388 , 2011)** | **582.5 (509.2 , 660)** | **2177 (1812 , 2589)** | **321.9 (263 , 379.6)** | **-44.7 (-55.7 , -32.2)** |
| **Egypt** | **4477548 (3814309 , 5302156)** | **5535.6 (4817.8 , 6467)** | **1023972 (755472 , 1353435)** | **1166.1 (884.6 , 1523)** | **-78.9 (-85 , -70.8)** |
| **Iran (Islamic Republic of)** | **1161357 (912538 , 1571576)** | **1563.8 (1280.6 , 2009.3)** | **255439 (232950 , 277268)** | **359 (326.5 , 390.5)** | **-77 (-82.8 , -71)** |
| **Iraq** | **572297 (439327 , 756419)** | **2007.8 (1573.9 , 2578.4)** | **159655 (123473 , 204684)** | **429.9 (342.4 , 532.3)** | **-78.6 (-85.2 , -70.3)** |
| **Jordan** | **53643 (41703 , 68430)** | **1172.3 (979.2 , 1417.3)** | **47065 (37082 , 60195)** | **508.6 (412.8 , 629.6)** | **-56.6 (-67.4 , -43.9)** |
| **Kuwait** | **9951 (8776 , 11429)** | **758.7 (690.7 , 832.5)** | **14477 (12161 , 17029)** | **596.5 (502 , 705.2)** | **-21.4 (-34.6 , -5.5)** |
| **Lebanon** | **29841 (23393 , 37990)** | **816 (671.7 , 984.4)** | **18592 (15416 , 23512)** | **360.1 (298.7 , 454.6)** | **-55.9 (-65.7 , -43.6)** |
| **Libya** | **52540 (38277 , 71721)** | **998.1 (783.7 , 1279.2)** | **22863 (18058 , 28781)** | **440 (349 , 547.3)** | **-55.9 (-68.3 , -39.3)** |
| **Morocco** | **929403 (728952 , 1163357)** | **2773.3 (2225 , 3417.2)** | **204076 (148367 , 271649)** | **686.5 (497.9 , 917.8)** | **-75.2 (-82.9 , -65.2)** |
| **Palestine** | **27414 (20309 , 37375)** | **1086 (861.8 , 1350.7)** | **16501 (13892 , 20029)** | **512.8 (436.6 , 653.4)** | **-52.8 (-63.2 , -40.7)** |
| **Oman** | **24077 (17932 , 32730)** | **1466.5 (1168.2 , 1799.6)** | **12839 (11233 , 14564)** | **692.8 (578 , 789.6)** | **-52.8 (-62.1 , -40.4)** |
| **Qatar** | **1440 (1090 , 1884)** | **579 (467.6 , 764)** | **2726 (2111 , 3636)** | **357.8 (288 , 448.1)** | **-38.2 (-52.9 , -20.6)** |
| **Saudi Arabia** | **125219 (97367 , 164175)** | **1076.6 (859.2 , 1312.7)** | **156031 (122635 , 199355)** | **662.3 (541.1 , 805.5)** | **-38.5 (-51.5 , -18.4)** |
| **Sudan** | **1390550 (797599 , 2402354)** | **3979 (2496.7 , 6601.5)** | **380930 (256106 , 552654)** | **951.9 (691.8 , 1271.9)** | **-76.1 (-85.7 , -62.8)** |
| **Syrian Arab Republic** | **266767 (196026 , 368054)** | **1385.3 (1075.7 , 1801.9)** | **79826 (60138 , 102141)** | **659.7 (507.6 , 835.4)** | **-52.4 (-65.8 , -34.2)** |
| **Tunisia** | **159741 (117655 , 217650)** | **1592.5 (1227.6 , 2090.4)** | **41698 (31643 , 54752)** | **386.1 (294.2 , 503.8)** | **-75.8 (-84.1 , -64.3)** |
| **Turkey** | **2455229 (1774867 , 3365227)** | **3325.7 (2466.3 , 4501)** | **309439 (248357 , 367147)** | **415.3 (339.1 , 488.7)** | **-87.5 (-91.2 , -83.1)** |
| **United Arab Emirates** | **7565 (6054 , 9503)** | **1316.6 (880.7 , 1565.6)** | **19275 (14480 , 26407)** | **728.2 (496.9 , 888.8)** | **-44.7 (-56.2 , -25.9)** |
| **Yemen** | **966218 (619526 , 1646844)** | **3777.3 (2581.7 , 5947)** | **363836 (243983 , 529232)** | **1175 (834.6 , 1563.3)** | **-68.9 (-80.5 , -53.3)** |
| **South Asia** | **69580660 (60124976 , 78956684)** | **4710.3 (4152.7 , 5290.5)** | **25525882 (22096613 , 29761535)** | **1652.7 (1444.1 , 1917)** | **-64.9 (-71.1 , -57.1)** |
| **Bangladesh** | **9088163 (7767356 , 10549179)** | **5492.6 (4732.3 , 6307.9)** | **1707249 (1327348 , 2123380)** | **1291.4 (1002.5 , 1609.4)** | **-76.5 (-83.1 , -69)** |
| **Bhutan** | **39409 (18522 , 61770)** | **4279.9 (2129.6 , 6599.6)** | **6681 (4526 , 9856)** | **1087.6 (749.8 , 1582.3)** | **-74.6 (-84.7 , -49.8)** |
| **India** | **51748742 (43678752 , 59742772)** | **4776.9 (4105.4 , 5454.2)** | **18526957 (16025321 , 21645113)** | **1631.6 (1411.2 , 1903.5)** | **-65.8 (-72.3 , -57.6)** |
| **Nepal** | **2342909 (1907638 , 2824569)** | **7036.6 (5830.7 , 8385.7)** | **424444 (336453 , 524167)** | **1570.6 (1263.3 , 1911.1)** | **-77.7 (-83 , -71.2)** |
| **Pakistan** | **6361438 (5117464 , 7613126)** | **3285.5 (2673.3 , 3896.4)** | **4860551 (3722331 , 6196827)** | **1809.4 (1408.1 , 2265.8)** | **-44.9 (-58.7 , -27.3)** |
| **Southern Sub-Saharan Africa** | **2460650 (2147640 , 2786864)** | **4347.7 (3903 , 4807.9)** | **1961107 (1693462 , 2282721)** | **2779 (2435.9 , 3193.4)** | **-36.1 (-44.9 , -24.8)** |
| **Botswana** | **42699 (32350 , 54880)** | **3886.2 (2945.1 , 5086.9)** | **65838 (49737 , 87600)** | **3417.8 (2587.8 , 4496.4)** | **-12.1 (-34.3 , 15)** |
| **Lesotho** | **104169 (86137 , 125363)** | **4966.1 (4200.6 , 5833.2)** | **93485 (72464 , 115884)** | **5125 (4013.7 , 6309)** | **3.2 (-19.7 , 31.3)** |
| **Namibia** | **63900 (46885 , 82034)** | **4436.3 (3340.5 , 5658.1)** | **56322 (39220 , 78442)** | **2791.1 (2028.8 , 3751.9)** | **-37.1 (-54.4 , -11.4)** |
| **South Africa** | **1707831 (1482501 , 1965072)** | **4382.8 (3882.8 , 4926.9)** | **1046453 (925575 , 1183552)** | **2104.9 (1880.6 , 2373.1)** | **-52 (-58.7 , -43.9)** |
| **Eswatini** | **46465 (34957 , 59423)** | **4704.3 (3733.9 , 5722.3)** | **37636 (27783 , 49660)** | **3665.5 (2726.5 , 4799.3)** | **-22.1 (-43 , 4.1)** |
| **Zimbabwe** | **495585 (382377 , 609369)** | **4513.8 (3735.9 , 5265)** | **661372 (516109 , 825453)** | **4900.8 (3948.2 , 5947.1)** | **8.6 (-15.4 , 40)** |
| **Western Sub-Saharan Africa** | **25345092 (20152056 , 32347726)** | **7949.4 (6500.8 , 9933.6)** | **23847090 (18927582 , 29481882)** | **4198.1 (3432.3 , 5041.9)** | **-47.2 (-58.6 , -32)** |
| **Benin** | **638371 (493534 , 803576)** | **7858.2 (6313.3 , 9520.6)** | **568687 (392855 , 777865)** | **3753.3 (2781 , 5007.6)** | **-52.2 (-65.6 , -34.7)** |
| **Burkina Faso** | **1372118 (1057383 , 1773907)** | **8720.1 (6987.2 , 10890.3)** | **1682103 (1210784 , 2226464)** | **5419.7 (4124.6 , 6853.1)** | **-37.8 (-53.5 , -18.1)** |
| **Cameroon** | **736834 (554009 , 970457)** | **5110.6 (4011.8 , 6422.2)** | **961413 (681180 , 1302705)** | **3438 (2554.9 , 4603.9)** | **-32.7 (-50.7 , -8.2)** |
| **Cabo Verde** | **10009 (7871 , 12702)** | **2355.5 (1959.6 , 2834.3)** | **6535 (5514 , 7626)** | **1421 (1205.8 , 1636)** | **-39.7 (-51.7 , -24)** |
| **Chad** | **921550 (690587 , 1178163)** | **8700.2 (6667.3 , 10952.1)** | **1389316 (1015611 , 1846949)** | **5716.6 (4533.9 , 7167.4)** | **-34.3 (-50.4 , -12.9)** |
| **CÃ´te d'Ivoire** | **1282544 (952692 , 1626628)** | **6999.8 (5485.5 , 8573.5)** | **1029358 (728271 , 1368035)** | **3744.2 (2809.9 , 4754.2)** | **-46.5 (-59.8 , -30.1)** |
| **Gambia** | **80480 (59049 , 105049)** | **5591.7 (4255.3 , 7088.2)** | **49388 (37418 , 63748)** | **2882 (2276.2 , 3520.6)** | **-48.5 (-62.2 , -30.3)** |
| **Ghana** | **731152 (536597 , 1004527)** | **4359.6 (3503.5 , 5431.7)** | **676289 (514311 , 867834)** | **2693.6 (2164.4 , 3264.4)** | **-38.2 (-53.4 , -20.1)** |
| **Guinea** | **1274509 (982537 , 1621943)** | **11481.8 (9132.8 , 14337.8)** | **819951 (591700 , 1110870)** | **5441.4 (4062.8 , 7128.1)** | **-52.6 (-65.8 , -35.4)** |
| **Guinea-Bissau** | **111387 (81977 , 148959)** | **8326.4 (6510.2 , 10767.5)** | **52613 (39907 , 68843)** | **3685.6 (2949.1 , 4567.2)** | **-55.7 (-67.6 , -40.4)** |
| **Liberia** | **314277 (236878 , 399063)** | **10242.1 (8010.2 , 12666)** | **89875 (64537 , 122218)** | **2323.2 (1750.9 , 3002.3)** | **-77.3 (-83.5 , -69.3)** |
| **Mali** | **794236 (596987 , 1020579)** | **5226.7 (4112 , 6510.9)** | **1134823 (810754 , 1528934)** | **3326.5 (2440 , 4358)** | **-36.4 (-54.2 , -12.1)** |
| **Mauritania** | **137542 (103171 , 176026)** | **5067.3 (4145 , 6124.4)** | **72471 (49664 , 104497)** | **2141.4 (1561 , 2934.6)** | **-57.7 (-70.2 , -43.5)** |
| **Niger** | **2108948 (1542558 , 2908954)** | **13770.9 (10371.9 , 18281.4)** | **1721123 (1225366 , 2325014)** | **4889.9 (3694.1 , 6365.3)** | **-64.5 (-75.5 , -49.3)** |
| **Nigeria** | **13285237 (9922209 , 17946869)** | **8646.6 (6571.2 , 11507.4)** | **12584302 (9714819 , 16081342)** | **4406.3 (3545.2 , 5424.4)** | **-49 (-62.5 , -30.1)** |
| **Sao Tome and Principe** | **9246 (7253 , 11435)** | **5760.7 (4692.7 , 6913.4)** | **3778 (2911 , 4840)** | **2623.7 (2075.5 , 3252.7)** | **-54.5 (-66 , -40.6)** |
| **Senegal** | **605484 (457029 , 760957)** | **5287.5 (4107.8 , 6400.8)** | **328420 (235960 , 429316)** | **2292.9 (1723.4 , 2882.6)** | **-56.6 (-68.9 , -41)** |
| **Sierra Leone** | **659733 (484258 , 849499)** | **11204.2 (8429.9 , 14066)** | **466274 (321426 , 636387)** | **4958.3 (3593.5 , 6532.5)** | **-55.7 (-68.2 , -39.1)** |
| **Togo** | **270586 (201165 , 359139)** | **5520.6 (4423.4 , 6850.8)** | **210039 (155475 , 281881)** | **3111.9 (2452 , 3914.5)** | **-43.6 (-57.2 , -27)** |
| **Eastern Sub-Saharan Africa** | **23277065 (19071654 , 28561040)** | **7849.7 (6650.3 , 9360.1)** | **11897920 (9658892 , 14747300)** | **2908.2 (2509.2 , 3423.6)** | **-63 (-69.9 , -54.3)** |
| **Burundi** | **591279 (428098 , 759835)** | **7289.2 (5641.6 , 9045.9)** | **354828 (239992 , 525174)** | **3125.3 (2321.2 , 4113.2)** | **-57.1 (-69.4 , -40.8)** |
| **Comoros** | **50092 (29133 , 69124)** | **6972.6 (4063.8 , 9371.1)** | **18765 (13860 , 24309)** | **2883.2 (2183.9 , 3626.5)** | **-58.6 (-69.4 , -28.3)** |
| **Djibouti** | **35692 (24742 , 49592)** | **5074.6 (3842.9 , 6640.5)** | **31935 (22779 , 44168)** | **2850.2 (2182.2 , 3742.6)** | **-43.8 (-59.5 , -21.5)** |
| **Eritrea** | **395253 (270533 , 553027)** | **9400.9 (6817 , 13059.3)** | **223926 (149397 , 327081)** | **4196 (2749.1 , 6479.9)** | **-55.4 (-72.9 , -32.9)** |
| **Ethiopia** | **8079440 (6365665 , 10099314)** | **10207.5 (8364.5 , 12222.7)** | **2454551 (1945280 , 3129937)** | **2415.7 (2067.8 , 2845.7)** | **-76.3 (-82.3 , -68.9)** |
| **Kenya** | **1575141 (1266622 , 1905221)** | **4747.7 (4056.7 , 5483.2)** | **982590 (805071 , 1179412)** | **2512.6 (2103.4 , 2970.6)** | **-47.1 (-56.9 , -35.6)** |
| **Madagascar** | **1331706 (1141227 , 1531857)** | **7217.9 (6304.2 , 8185.8)** | **782027 (595285 , 1006808)** | **3082.6 (2451 , 3838.7)** | **-57.3 (-67.7 , -45.6)** |
| **Malawi** | **1092944 (850871 , 1385011)** | **7279.3 (5875.6 , 8888.6)** | **512598 (389380 , 671003)** | **2986.5 (2443.7 , 3665.3)** | **-59 (-68 , -46.8)** |
| **Mozambique** | **1594383 (1055326 , 2416411)** | **7545.5 (5324.3 , 10863.2)** | **903597 (661962 , 1200559)** | **3006.2 (2364.8 , 3718.1)** | **-60.2 (-73.7 , -42)** |
| **Rwanda** | **910170 (673956 , 1180370)** | **8427.6 (6495.6 , 10535.1)** | **285257 (215725 , 383980)** | **2528.1 (2005.2 , 3185.1)** | **-70 (-78.2 , -59.2)** |
| **Somalia** | **1105913 (730214 , 1518835)** | **10089.2 (7221 , 13573.5)** | **1489120 (1013233 , 2073389)** | **5596.5 (4096.1 , 7508.7)** | **-44.5 (-59.2 , -23.3)** |
| **South Sudan** | **837468 (611627 , 1138943)** | **9033.3 (6844.4 , 11842.4)** | **540784 (381433 , 729004)** | **4331.1 (3285.9 , 5571.5)** | **-52.1 (-64.4 , -36.6)** |
| **United Republic of Tanzania** | **3381863 (2615757 , 4281757)** | **7994.6 (6456.7 , 9731.5)** | **1945837 (1456428 , 2534535)** | **3021.4 (2440.1 , 3717)** | **-62.2 (-70.5 , -50.6)** |
| **Uganda** | **1192674 (807930 , 1643844)** | **4336.8 (3230.8 , 5610.1)** | **900886 (664927 , 1216277)** | **2355.5 (1884.7 , 2883.2)** | **-45.7 (-58.9 , -26.3)** |
| **Zambia** | **1085965 (853208 , 1381503)** | **8347.4 (6847 , 10258.3)** | **461709 (354014 , 606099)** | **2715.3 (2209.6 , 3328.6)** | **-67.5 (-75.3 , -57.3)** |
| **Central Sub-Saharan Africa** | **6308809 (4788682 , 8112399)** | **7412.7 (5908.5 , 9091.4)** | **3388970 (2622033 , 4286609)** | **2964.6 (2364.4 , 3664.5)** | **-60 (-69 , -49.1)** |
| **Angola** | **1528800 (963554 , 2341219)** | **8929.1 (6069.9 , 12836.7)** | **724671 (535955 , 941524)** | **2555.8 (2031.4 , 3197.9)** | **-71.4 (-81.9 , -54.6)** |
| **Central African Republic** | **359232 (249394 , 513852)** | **8945.4 (6824.6 , 11923.4)** | **372860 (255939 , 529494)** | **6145.4 (4444 , 8428.8)** | **-31.3 (-50.9 , -5)** |
| **Congo** | **120467 (88387 , 162664)** | **4496.5 (3449.9 , 5794.5)** | **77707 (59882 , 98750)** | **2063.1 (1615.7 , 2571.7)** | **-54.1 (-65 , -40.9)** |
| **Democratic Republic of the Congo** | **4199123 (3108253 , 5426611)** | **7156.5 (5481.9 , 8997)** | **2176778 (1589940 , 2888666)** | **2988 (2268.1 , 3832.8)** | **-58.2 (-68.4 , -45.9)** |
| **Equatorial Guinea** | **56189 (36762 , 85827)** | **7695.1 (5467 , 10936.1)** | **14515 (9383 , 21985)** | **1697.3 (1203.6 , 2364)** | **-77.9 (-86.4 , -66.2)** |
| **Gabon** | **45000 (34481 , 57561)** | **3853.7 (3024.5 , 4776.9)** | **22439 (16774 , 28574)** | **1731.2 (1304.4 , 2161.4)** | **-55.1 (-65.7 , -42.4)** |
